# The box C/D snoRNP assembly factor Bcd1 interacts with the histone chaperone Rtt106 and controls its transcription dependent activity

**DOI:** 10.1038/s41467-021-22077-4

**Published:** 2021-03-25

**Authors:** Benoît Bragantini, Christophe Charron, Maxime Bourguet, Arnaud Paul, Decebal Tiotiu, Benjamin Rothé, Hélène Marty, Guillaume Terral, Steve Hessmann, Laurence Decourty, Marie-Eve Chagot, Jean-Marc Strub, Séverine Massenet, Edouard Bertrand, Marc Quinternet, Cosmin Saveanu, Sarah Cianférani, Stéphane Labialle, Xavier Manival, Bruno Charpentier

**Affiliations:** 1 Université de Lorraine, CNRS, IMoPA, Nancy, France; 2 Laboratoire de Spectrométrie de Masse BioOrganique, Université de Strasbourg, CNRS IPHC UMR 7178, Strasbourg, France; 3 Génétique des Interactions Macromoléculaires, Département de Génomes et Génétique, Institut Pasteur (UMR3525-CNRS), Paris, France; 4 IGH, CNRS, Université de Montpellier, Montpellier, France; 5 Université de Lorraine, CNRS, INSERM, IBSLor, Nancy, France; 6 Present Address: Department of Biochemistry and Molecular Biology, Mayo Clinic, Rochester, MN USA; 7 Present Address: Ecole polytechnique fédérale de Lausanne (EPFL) SV ISREC, Station 19, Lausanne, Switzerland

**Keywords:** Chromatin remodelling, Small RNAs, Transcription, Structural biology

## Abstract

Biogenesis of eukaryotic box C/D small nucleolar ribonucleoproteins initiates co-transcriptionally and requires the action of the assembly machinery including the Hsp90/R2TP complex, the Rsa1p:Hit1p heterodimer and the Bcd1 protein. We present genetic interactions between the Rsa1p-encoding gene and genes involved in chromatin organization including *RTT106* that codes for the H3-H4 histone chaperone Rtt106p controlling H3K56ac deposition. We show that Bcd1p binds Rtt106p and controls its transcription-dependent recruitment by reducing its association with RNA polymerase II, modulating H3K56ac levels at gene body. We reveal the 3D structures of the free and Rtt106p-bound forms of Bcd1p using nuclear magnetic resonance and X-ray crystallography. The interaction is also studied by a combination of biophysical and proteomic techniques. Bcd1p interacts with a region that is distinct from the interaction interface between the histone chaperone and histone H3. Our results are evidence for a protein interaction interface for Rtt106p that controls its transcription-associated activity.

## Abstract (web summary)

Biogenesis of small nucleolar RNAs ribonucleoproteins (snoRNPs) requires dedicated assembly machinery. Here, the authors show that a subset of snoRNP assembly factors interacts, genetically or directly, with factors modulating chromatin architecture, suggesting a link between ribosome formation and chromatin functions.

## Introduction

Small nucleolar RNAs (snoRNAs) form a large abundant family of noncoding RNAs predominantly localized in the nucleolus^1^. Based on conserved sequence motifs, snoRNAs fall into one of two classes—box C/D snoRNAs or box H/ACA snoRNAs. Each snoRNA associates with a set of class-specific and well-characterized core proteins to form ribonucleoprotein (RNP) complexes referred to as small nucleolar ribonucleoproteins, i.e., box C/D snoRNPs and box H/ACA snoRNPs. These particles primarily catalyze post-transcriptional modifications in ribosomal RNAs^2^ (rRNAs). In these reactions, the snoRNA functions as a guide by base-pairing with a target sequence and selecting the precise nucleotide that will be modified by the catalytic activity of the snoRNP^3^. A few other snoRNPs, e.g., U3, are involved in endo-ribonucleolytic processing of the original pre-rRNA transcript^4,5^.

Eukaryotic snoRNP biogenesis is a complex process that involves coordinated assembly, RNA processing, and localization factors^6^. In yeast, box C/D snoRNPs are formed by assembling core proteins Snu13, Nop56, Nop58, and 2’-*O*-methyl transferase Nop1 on box C/D snoRNAs. This process requires assembly machinery including the protein heterodimer Rsa1:Hit1^7^ and the R2TP chaperone complex, which is composed of proteins Pih1, Tah1, and AAA+ ATPases Rvb1 and Rvb2 (Rvb1/2)^8,9^. In addition, the Bcd1 protein is essential for both cell viability and box C/D snoRNA steady-state stability^10,11^, and was shown to control the loading of Nop58p to pre-snoRNPs^12^. A conserved motif in the N-terminal region of Bcd1p was proposed as a determinant for binding with Snu13p and snoRNA^13^. This tripartite R2TP/Rsa1p:Hit1p/Bcd1p machinery is conserved in metazoans and plants where it is also used for snoRNP biogenesis^8,14–18^. The human ortholog of R2TP associates with an additional prefoldin-like (PFDL) module to form a larger chaperone complex called the PAQosome^19^. This chaperone is involved in the folding of critical macrocomplexes, e.g., RNPs involved in fundamental cellular processes, and RNA polymerases.

Here we show that the snoRNP assembly factor gene *RSA1* genetically interacts with genes involved in chromatin structure and nucleosome assembly including histone chaperones such as Rtt106p (Regulator of Ty transposition), as well as with genes involved in transcription and RNA processing. We also demonstrate that the box C/D snoRNP assembly factor Bcd1p binds Rtt106p directly. This chaperone cooperates with the H3-H4 histone chaperone CAF-1 (chromatin assembly factor 1) and with the heterodimeric chaperone FACT (facilitates chromatin transcriptions, composed of the Spt16p:Pob3p heterodimer) to facilitate DNA replication-coupled (RC) nucleosome assembly^20,21^. During this process, Rtt106p participates in the deposition of newly synthesized H3K56ac-carrying H3:H4 complex on replicating DNA^22–26^. In addition, Rtt106p is involved in replication-independent pathways including heterochromatin silencing^20,27^, HIR-dependent control of histone gene expression^28^, regulation of transcription-dependent histone H3 deposition during RNA polymerase elongation^29^, and maintenance of promoter fidelity in cooperation with HIR (histone regulatory) and Asf1 (anti-silencing factor 1) proteins^30^.

Our data reveal a Bcd1p-dependent association of Rtt106p on box C/D pre-snoRNAs, with no discernible effect on snoRNP assembly. On the contrary, we observe that the association of Rtt106p with transcriptionally active loci correlates with its association with RNA polymerase II, and that these associations are negatively modulated by Bcd1p. In coherence with these observations, we show that H3K56ac levels are increased at several gene bodies upon Bcd1p depletion. We characterize this important Bcd1p:Rtt106p interaction for the transcription-dependent Rtt106p function at the molecular and atomic levels. A combination of ITC, MS approaches, NMR, and X-ray show that Bcd1p binds with the PH1 (Pleckstrin-Homology 1) domain of Rtt106p. These data support a model in which the binding of Bcd1p to Rtt106p can inhibit the transcription-related activities of this histone chaperone.

## Results

### Genetic Interaction Mapping (GIM) screens of the C/D snoRNP assembly factor gene *RSA1*

**Fig. 1:**
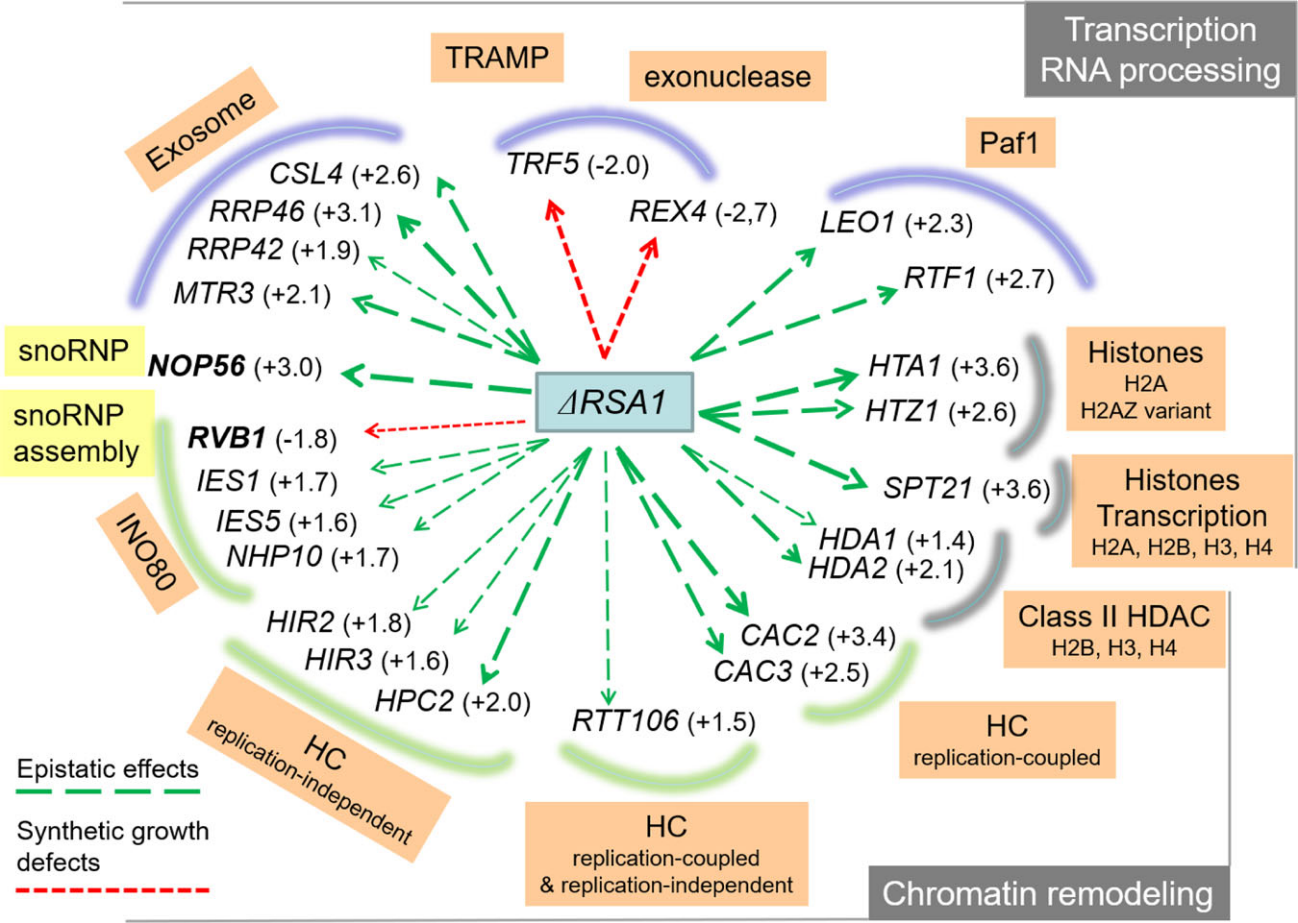
Data from the GIM screen. Two independent screens were performed, one with a mutant strain in which the *RSA1* gene was deleted (*rsa1*Δ*::Nat*^*R*^*)* and the other with a mutant used as reference strain. Two independent experiments were performed for each of these screens. The query strain and the reference strain were mated with a pool of strains containing all the viable strains from the haploid gene deletion collection. After selection of heterozygous diploids, sporulation and selection of the haploid, double mutants were grown for ~18 generations in rich liquid medium (YPD). Microarrays were used to measure the relative abundance of double mutants with query versus the reference populations. Normalized results are expressed as log2(Q/R) (see Methods section). Negative values indicate a synthetic growth defect. Positive values reveal either epistatic (buffering) or suppressive (alleviating) interactions between *RSA1* and the selected genes. The genetic interactions with *RSA1* are indicated by green arrows for the positive log2(Q/R) values and by red arrows for the negative values. The exact values are given in parentheses. HC heterochromatin.

To identify potential genetic interactions with the snoRNP assembly machinery, we performed a high-throughput genetic screen. We used deletion of *RSA1* (*rsa1*Δ::*prMFα2Nat*^*R*^) as the query mutation and combined it in a single pool by mating it with individual mutants of the yeast systematic deletion library of nonessential genes^31^ and with a collection of DAmP (decreased abundance by mRNA perturbation) mutants for essential genes^32,33^. Double mutants were selected from the pool after sporulation and grown for ~18 generations in rich liquid medium. In parallel, a similar screen was performed with other mutant strains for use as a control population. The relative growth rates of double mutant strains were estimated by measuring the relative abundance of cells in the query versus reference populations using DNA barcodes and microarrays. Normalized results are expressed as log2(Q/R) where Q represents the signal intensities of the tag marking a given mutant when combined with the query mutation, and R is the signal from the same mutant when introduced in the reference population. In agreement with previous GIM screens, we obtained a relatively large number of both negative and positive log2(Q/R) values, representing aggravating and buffering (or alleviating) effects, respectively (Source data file). In agreement with the contribution of Rsa1p to snoRNP biogenesis and validating the approach, we observed strong genetic links between *RSA1* and *RVB1*, encoding one of the R2TP components, and *NOP56*, encoding one of the box C/D core proteins (Fig. 1). However, whereas a negative value (log2(Q/R) = −1.8) was obtained for the *rvb1-DAMP* mutation pointing to a synthetic growth defect, combining *rsa1*Δ mutation with the *nop56-DAMP* mutation had an epistatic effect with a positive value (log2(Q/R) = +3.0).

We used g:Profiler [https://biit.cs.ut.ee/gprofiler/gost] to check if specific annotations were enriched among our results. The list of the top 50 mutants showing an epistatic or suppressor effect in combination with *rsa1*Δ was enriched in two broad gene ontology (GO) categories: mRNA metabolic process (GO:0016071) and chromatin organization (GO:0006325). For a more detailed view of which specific processes among RNA metabolism and chromatin organization were affected by *RSA1* deletion, we used a curated list of 518 yeast complexes^34^ and searched for gene set enrichment in the GIM screen data using GAGE^35^. Among the top results, we found genes, e.g., *SPT21*, encoding proteins implicated in transcriptional silencing and required for normal transcription at other loci including *HTA2-HTB2* and *HHF2-HHT2* that encode histone proteins. Deletion of genes involved in termination and processing of the 3’ end of pre-snoRNAs rescued growth of *rsa1*Δ cells, including the exosome components *MTR3*, *RRP42*, *RRP46*, and *CSL4*, as well as the *LEO1* and *RTF1* subunits of the PAF complex, which is involved in transcription elongation (Fig. 1). It is conceivable that impairing these RNA processing events compensates for the imperfect box C/D snoRNP assembly resulting from the *rsa1*Δ disruption. Assembly of snoRNP starts early on nascent snoRNAs and this process has been proposed to be coupled to 3’ processing^36,37^. One possibility is that RNP assembly is delayed in the *rsa1*Δ mutant and becomes kinetically uncoupled from 3’ processing. Slower transcription termination and 3’ processing would then compensate the slower RNP assembly.

In addition, deletion of *RSA1* had multiple epistatic effects when combined with deletion of genes encoding components of the chromatin remodeling complexes HIR (*HIR2*, *HIR3*, *HPC2*), INO80 (*IES1*, *IES5*, *NHP10*), and CAF-1 (*CAC2* and *CAC3*), as well as genes *HDA1* and *HDA2* encoding members of the HDA1 histone deacetylase complex and genes encoding histone variants (*HTZ1* and *HTA1*; Fig. 1). Interestingly, *rsa1*Δ had a positive log2(Q/R) value (+1.5) in combination with the deletion of *RTT106*, which is connected to the HIR and CAF-1 complexes^27,28^. We focused on the Rtt106 protein as it is linked to several fundamental functions such as DNA replication, stability, and transcription, but not yet to snoRNP biogenesis and function, except for the observation based on modified chromatin immunopurification^38^ (mChIP) suggesting that it can associate with complexes containing Rvb1/2 proteins in the vicinity of chromatin.

### Rtt106p binds Bcd1p

**Fig. 2:**
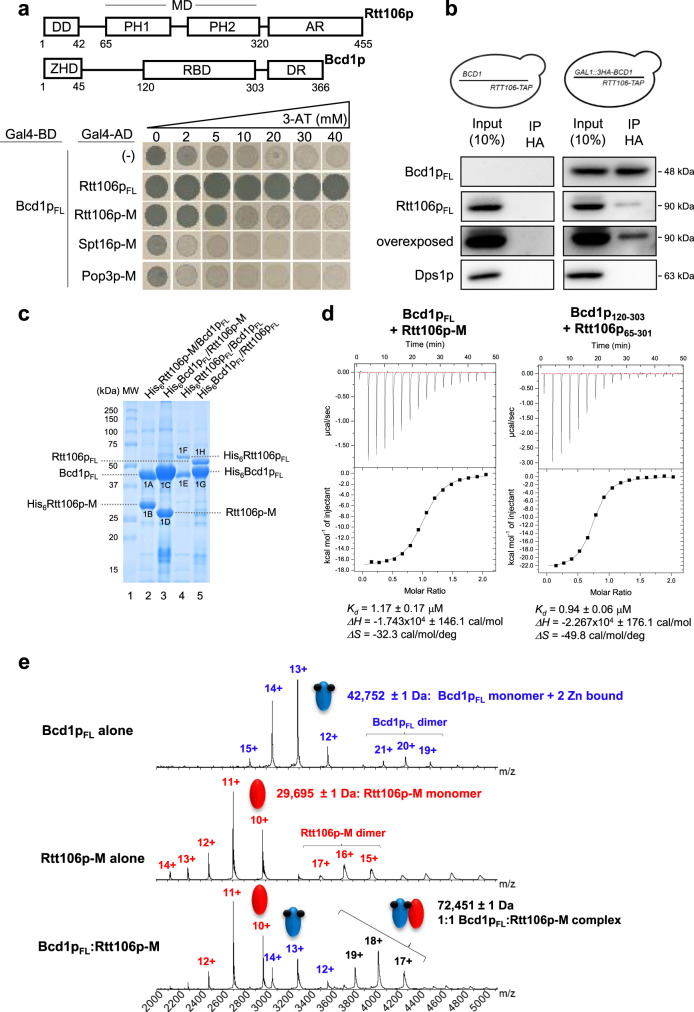
Bcd1p and histone chaperone Rtt106p form a stable heterodimer. **a** Schematic representation of the Rtt106p and Bcd1p domain organization. DD dimeric domain, PH Plekstrin homology, PH1 + PH2 = MD middle domain, AR acidic region, ZHD zinc finger HIT domain, RBD Rtt106 binding domain, DR disorder region. Y2H assay: full-length Bcd1p (Bcd1p_FL_) fused to the Gal4 binding domain (BD) interacts with the full-length Rtt106p (Rtt106p_FL_) or fragment spanning amino acids 65–320 (Rtt106p-M) fused to the Gal4 activation domain (AD) as evidenced by growth on a His deprived medium supplemented with increased concentrations of 3-amino-1, 2, 4-triazol (3-AT). No interaction was observed with homologous fragments of FACT chaperone components Spt16p and Pob3p (Spt16p-M and Pob3p-M). **b** Interaction of Bcd1p_FL_ with Rtt106p_FL_ in yeast. Co-immunoprecipitation (co-IP) was performed on *GAL1::3HA-BCD1* × *RTT106-TAP* cells expressing 3xHA-tagged Bcd1p_FL_ and TAP-tagged Rtt106p_FL_. Cells expressing the nontagged Bcd1p were used as negative control. Extracts were incubated with anti-HA beads. The co-immunoselected proteins were analyzed by SDS-PAGE and western blotting. 10% of total proteins used per assay were loaded in the input lane. Tagged proteins were detected with PAP antibodies for Rtt106p and anti-HA antibodies for Bcd1p. The Dps1 protein used to control protein loading was detected using specific anti-Dps1p antibodies. The two panels correspond to a cropping of two sections of the same membrane. The full-length membrane is presented in the Source data file. The experiment was independently repeated three times with similar results. **c** Interaction of recombinant Bcd1p and Rtt106p in *E. coli*. His_6_-tagged full-length or M domain of Rtt106p were co-expressed with Bcd1p_FL_. His_6_-tagged Bcd1p_FL_ was co-expressed with Rtt106p_FL_ or Rtt106p-M. Complexes were selected from crude extract by immobilized metal ion affinity chromatography (IMAC), fractionated by SDS-PAGE and revealed by Coomassie blue staining. The results correspond to co-expression with high salt (400 mM) buffer. Molecular weight markers (MW) in kilo Dalton (kDa) were loaded on the left. The experiment was repeated twice with similar results. The identity of the proteins in bands 1 A, 1B, 1 C, 1D, 1E, 1 F, 1 G, and 1H was confirmed by in-gel digestion of gel slices and mass spectrometry (MS) analysis of the peptide extract (Supplementary Table 1). **d** Bcd1p and Rtt106p interacting domains. ITC data for the interaction of Bcd1p_FL_ with Rtt106p-M (on the left) and Bcd1p_120-303_ with Rtt106p_65-301_ (on the right) recorded at 293 K in buffer containing 10 mM NaPi at pH 7.5, 150 mM NaCl and 0.5 mM TCEP. The calculated affinities *K*d, and thermodynamic parameters as variations in enthalpy (Δ*H*) and entropy (Δ*S*) are indicated. **e** Nondenaturing MS characterization of the complex formed upon incubation of recombinant Bcd1p_FL_ with fragment Rtt106p-M. NanoESI mass spectra performed under nondenaturing conditions confirmed the presence of a 1:1 binding stoichiometry of Bcd1p_FL_:Rtt106p-M complex (Da = Dalton). Source data for panel **b** are provided as a Source Data file.

To investigate interactions between Rtt106p and the proteins known to be involved in snoRNP biogenesis, we performed systematic pairwise yeast two-hybrid (Y2H) assays. This revealed a strong in vivo interaction between Rtt106p and Bcd1p, which resisted high concentrations of 3-AT (30–40 mM; Fig. 2a). The Y2H interaction derived from the middle domain of Rtt106p encompassing the tandem PH1 and PH2 domains. Indeed, full-length Bcd1p (Bcd1p_FL_) interacted with the fragment of Rtt106p spanning amino acids 65–320 (Rtt106p_65-320_ also named Rtt106p-M, Fig. 2a). This interaction was specific as it was not detected with the homologous PH domains from histone chaperones Pob3p and Spt16p (Fig. 2a) that are members of the FACT chromatin remodeling complex^22,39,40^.

To validate the Bcd1p:Rtt106p interaction, we used pull-down assays in yeast cells engineered to express the tagged proteins 3xHA-Bcd1p and Rtt106-TAP from their genome (Fig. 2b). Retention of 3xHA-Bcd1p on beads occurred with the co-purification of Rtt106p-TAP. To determine whether this interaction is direct, we performed co-purification assays in *Escherichia coli* upon ectopic co-expression of different combinations of truncated or full-length His_6_-tagged and untagged proteins (Fig. 2c). The co-expressed proteins in the bacterial extract were co-purified by immobilized metal ion affinity chromatography (IMAC), fractionated by SDS-PAGE and analyzed by mass spectrometry (MS) (Supplementary Table 1). This confirmed the formation of complexes Bcd1p_FL_:Rtt106p_FL_ and Bcd1p_FL_:Rtt106p-M. Recombinant Bcd1p_FL_ and the fragment Rtt106p-M were also independently purified and isothermal titration calorimetry (ITC) revealed direct and endothermic in vitro binding with a 1.17 ± 0.17 μM dissociation constant (*K*_d_; Fig. 2d, left panel).

We completed these data by native MS analysis of the co-expressed complex Bcd1p_FL_:Rtt106p-M and its individual subunits (Fig. 2e). Bcd1p_FL_ was mainly detected as a monomer in interaction with two zinc ions (42,752 ± 1 Da, Fig. 2e upper spectrum), as already reported^41^. For Rtt106p-M, monomeric species were also mainly detected (29,695 ± 1 Da, Fig. 2e middle spectrum). Finally, native MS revealed the formation of a 1:1 Bcd1p_FL_:Rtt106p-M heterodimer with a mass of 72,451 ± 1 Da. These data demonstrated that Bcd1p_FL_ binds the 65–320 (M) domain of Rtt106p with 1:1 stoichiometry.

We then monitored the Bcd1p_FL_:Rtt106p-M heterodimer formation by ion mobility coupled with mass spectrometry (IM-MS). This approach makes it possible to determine collision cross section (^TW^CCS_N2_) by measuring the drift time of ions through a gas-filled IM cell. The arrival drift time (ADT) is closely linked to the charge state and to the shape of the ions in the gas phase^42^. IM-MS data and ^TW^CCS_N2_ measured for each individual subunit Bcd1p_FL_ and Rtt106p-M and for the Bcd1p_FL_:Rtt106p-M heterodimer (Supplementary Fig. 1) revealed that a significant conformational change occurs in one or both proteins upon heterodimer formation. The IM-MS measured ^TW^CCS_N2_ (44.4 ± 1.4 nm^2^) was larger than the value expected from mass and 15% smaller than the one predicted from a juxtaposition of the two subunits. We concluded that the heterodimer is not formed merely by simple juxtaposition of the two partners.

### Rtt106p binds pre-snoRNPs but does not appear to contribute to or interfere with snoRNP biogenesis

**Fig. 3:**
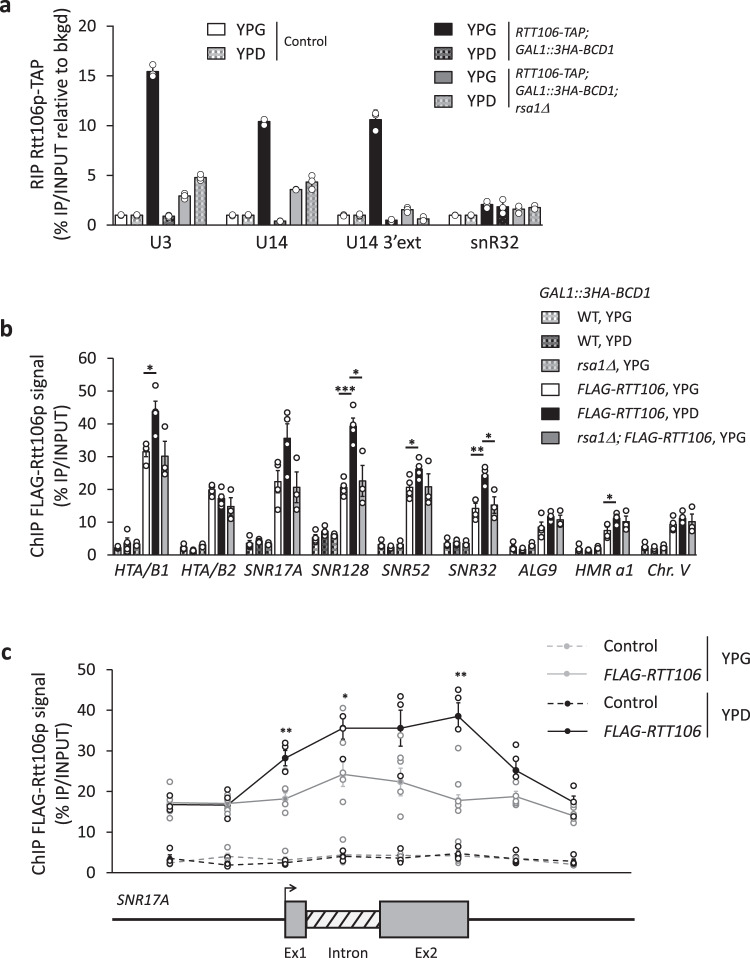
Interaction of Rtt106p with selected transcripts and loci. **a** RNA immunoprecipitation (RIP) assays were performed on extracts prepared from *RTT106-TAP*; *GAL1:3HA-BCD1* cells disrupted (*rsa1*Δ) or not of the *RSA1* ORF. The cells were cultivated in the presence of galactose (YPG) or shifted to a glucose-containing medium (YPD) for 6 h before preparation of the extract. IP was performed using IgG-sepharose beads. RNAs retained on beads were quantified by RT-qPCR as described in the Methods section. The snoRNAs and pre-snoRNAs analyzed are indicated. **b** Chromatin immunoprecipitation (ChIP) assays were performed on yeast *GAL1:3HA-BCD1* cells transformed with recombinant plasmid *FLAG-RTT106*; with (*rsa1*Δ) or without the disruption of the *RSA1* ORF. Cells were cultivated in YPG or shifted to YPD medium before ChIP assays. Cells expressing nontagged Rtt106p (*GAL1:3HA-BCD1* and *GAL1:3HA-BCD1; rsa1*Δ) were used as controls and IP was performed using anti-FLAG antibody. The loci analyzed by qPCR analysis are indicated. **c** Chip assays performed as in panel **b** on *GAL1:3HA-BCD1* cells transformed with recombinant plasmid *FLAG-RTT106*. Primers were used for qPCR analysis on both side and across the U3 encoding gene (*SNR17A*). Data reported in the panels a and c are mean values plus standard error of the mean of three biological replicates (*n* = 3). Data reported in panel **b** are mean values plus standard error of the mean of three (for *GAL1::3HA-BCD1, rsa1*Δ + p413 (−) and *GAL1::3HA-BCD1, rsa1*Δ + p413::*FLAG-RTT106*; *n* = 3) or four (for *GAL1::3HA-BCD1* + p413 (−) and *GAL1::3HA-BCD1* + p413::*FLAG-RTT106*; *n* = 4) biological replicates. Two-tailed *t*-tests: *P < 0.05, **P < 0.01, ***P < 0.001. In panel **b**, for *FLAG-RTT106* YPG versus YPD: * = 0.012 for *HTA/B1*, *** = 0.0006 for *SNR128* (U14), * = 0.045 for *SNR52*, ** = 0.002 for *SNR32*, * = 0.032 for *HMR a1*. Note that *SNR17A* (U3) is close to significance: *P* = 0,056. For *FLAG-RTT106* YPG versus *rsa1*Δ; *FLAG-RTT106* YPG: * = 0.02 for *SNR128* (U14) and * = 0.017 for *SNR32*. In panel **c**, for *FLAG-RTT106* YPG versus YPD: ** = 0.006, * = 0.031, and ** = 0.001 from the left to the right. Source data for all these panels are provided as Source Data files.

To investigate if Rtt106p contributes to snoRNP biogenesis, we used RNA immunoprecipitation (RIP) assays. We observed that Rtt106p-TAP was highly enriched on box C/D snoRNAs U3, U14 and the precursor form of U14 (Fig. 3a). Moreover, using *GAL1::3HA-BCD1* cells grown in galactose-containing (YPG) medium for expression of Bcd1p and in glucose-containing (YPD) medium for depletion of Bcd1p^12^, we showed that the association of Rtt106p with these snoRNAs is Bcd1p dependent. Using the *rsa1*Δ mutant cells, we showed that binding of Rtt106p with RNPs containing Bcd1p is also Rsa1p-dependent, which could be explained by the fact that a detectable association of Bcd1p with snoRNA species is Rsa1p-dependent (Supplementary Fig. 2a). Although these data revealed the presence of Rtt106p on box C/D pre-snoRNPs, the hypothesis of a role for Rtt106p in facilitating or modulating the function of Bcd1p in snoRNP biogenesis seemed unlikely. Indeed, the steady-state levels of box C/D pre-snoRNAs and their mature forms did not change in absence of Rtt106p expression, at least in yeast cells grown in standard culture conditions (Supplementary Fig. 2b). Accordingly, enrichments of Bcd1p to box C/D snoRNAs (Supplementary Fig. 2c) and to snoRNA gene loci did not change appreciably in the absence of Rtt106p (Supplementary Fig. 2d).

### Bcd1p regulates the presence of Rtt106p at transcriptionally active loci

To investigate a potential regulatory function of Bcd1p in Rtt106p activity, we checked whether the recruitment of Rtt106p to DNA loci could be Bcd1p dependent. Using ChIP, we analyzed the presence of FLAG-tagged Rtt106p to the HIR-dependent histone genes *HTA1*-*HTB1* and to the *HMR* locus where Rtt106p has been shown to be enriched^26,30,43,44^. Enrichment in *GAL1::3HA-BCD1* cells at box C/D snoRNA-encoding genes *SNR17A* (U3), *SNR128* (U14), *SNR52* (snR52), and *SNR32* encoding box H/ACA snoRNA snR32, as well as at the protein encoding gene *ALG9* was tested for the purpose of comparison (Fig. 3b). Enrichment of Rtt106p at either C/D box or H/ACA box snoRNA gene loci was equivalent to enrichment at *HTA1*-*HTB1* locus. Therefore, as described for coding genes that are actively transcribed^29,45^, Rtt106p likely acts as a histone chaperone at snoRNA gene loci. Disruption of the *RSA1* gene has no effect on the presence of Rtt106p to these loci. However, we observed that this enrichment increased significantly in the absence of *BCD1* gene expression, i.e., in YPD medium, at actively transcribed loci, but not at a chromosome V region devoid of transcription (Fig. 3b). To confirm that Bcd1p affects the transcription-related recruitment of Rtt106p, we solved the pattern of association at the U3 actively transcribed loci (Fig. 3c). Rtt106p enrichment increased in the body of the gene compared to the flanking intergenic regions, and the association was sensitive to the level of expression of Bcd1p only in the transcribed region. To test the possibility that association to chromatin relied on RNA, we performed ChIP analyses in the presence of RNase treatment (Supplementary Fig. 2e). Interestingly, we observed a slight reduction of ChIP signals at both transcribed and untranscribed loci, suggesting that Rtt106p associated moderately via undetermined chromatin-associated RNA species. Importantly, the effect of RNase treatment was similar in cells expressing or not Bcd1p, excluding the possibility that it occurs in a Bcd1p-dependent way. Therefore, the data suggest that Rtt106p interacts with chromatin by several, independent ways with at least one involving RNAs present at the vicinity of chromatin (including at untranscribed loci), and a second one that is independent from RNA but dependent on Bcd1p expression level.

### Bcd1p controls the association of Rtt106p with RNA polymerase II and H3K56ac levels at gene bodies

**Fig. 4:**
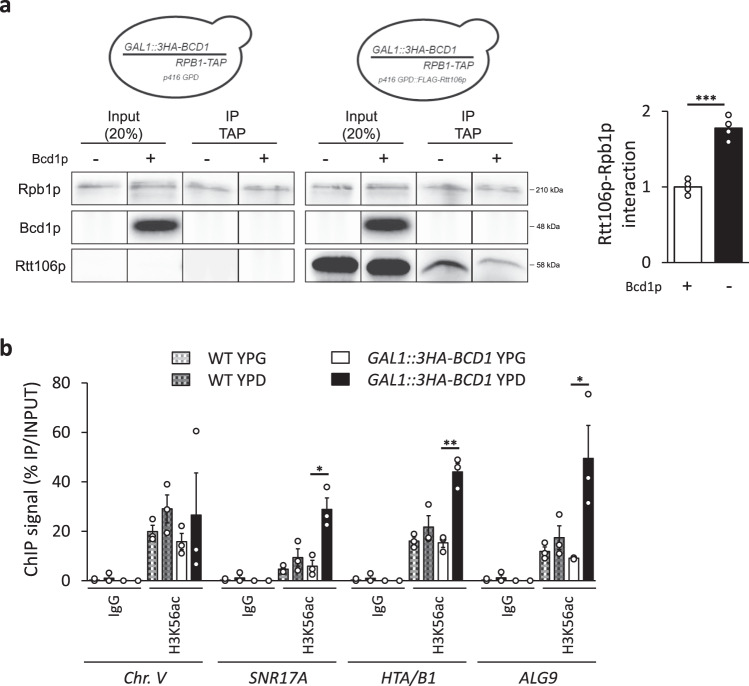
Bcd1p controls transcription-dependent activity of Rtt106p. **a** Interaction of Bcd1p with the RNA polymerase II large subunit Rpb1p in yeast. Co-immunoprecipitation (co-IP) was performed on *GAL1::3HA-BCD1* × *RPB1-TAP* cells transformed with p416GDP::FLAG-Rtt106p expressing 3xHA-tagged Bcd1p, TAP-tagged Rpb1p and FLAG-Rtt106p. Cells were transformed with empty vector p416GDP as a control. Cells were grown in YPG (+Bcd1p) or YPD (−Bcd1p). Procedure was as described in Fig. 2b but with the IP performed with anti-TAP antibodies. The image corresponds to a cropping of different sections of the same membrane. The full-length membrane incubated with different antibodies is presented in the Source data file. Histogram on the right presents quantification of Western blots obtained from six independent co-IP experiments. Quantification was performed using Fusion Solo (Vilber Lourmat) and Fusion-Capt Advance Solo 4 software. The FLAG signal (Rtt106p) was normalized to the TAP signal (Rpb1p). The data represent mean values plus standard error to the mean of four biological replicates. **b** Depletion of Bcd1p affects H3K56ac levels at several RNA polymerase II-dependent genes. ChIP H3K56ac enrichment (IP/INPUT) for parental BY4741 strain and the *GAL1::3HA-BCD1* strain grown in YPG (+Bcd1p) or YPD (−Bcd1p) is presented. Signal specificity was controlled with IgG antibodies. The histogram represents mean values plus standard error to the mean of three biological replicates. Two-tailed *t*-tests: *P < 0.05, **P < 0.01, ***P < 0.001. In panel **a**, *** = 0.0001; in panel **b**, * = 0.011 for *SNR17A* (U3), ** = 0.002 for *HTA/B1*, * = 0.039 for *ALG9*. Source data for these panels are provided as Source Data files.

As it has been proposed that Rtt106p acts in the wake of the transcriptional machinery to promote new histone deposition^29^, we first tested whether an interaction between Rtt106p and RNA polymerase II was detectable. Using co-IP, we indeed observed that Rtt106p was associated with Rpb1p, the largest RNA polymerase II subunit (Fig. 4a). Interestingly and in agreement with an effect of Bcd1p on transcription-related Rtt106p function, this association increased in the absence of *BCD1* gene expression. Taken together, these data suggest that direct binding of Bcd1p with Rtt106p precludes association of the histone chaperone with the RNA polymerase II, and therefore reduces its presence at transcriptionally active loci. In agreement with this proposal, the presence of histone H3 acetylated at lysine 56 (H3K56ac) was found to be higher in these transcribed regions upon Bcd1p depletion (Fig. 4b). We concluded that Bcd1p negatively regulates the histone chaperone activity of Rtt106p during transcription.

### Identification of the Rtt106p binding domain in Bcd1p

Sequence analysis of Bcd1p did not enable us to predict the existence of known domain(s) except the zf-HIT domain (ZHD) in the N-terminal part of the protein^41^ (Fig. 2a). To delineate the structural domain that engages Rtt106p, we performed partial proteolysis followed by native MS analysis of the co-purified Bcd1p:His_6_Rtt106p-M complex. We were able to identify several fragments of Bcd1p that maintain binding with His_6_Rtt106p-M, with most fragments spanning amino acids 113–366 (Supplementary Fig. 3). Guided by these data, we tested the potential interaction of Rtt106p-M with various fragments of Bcd1p by ITC (Fig. 2d right panel and Supplementary Fig. 4). In these studies, the shortest fragment of Bcd1p that conserved the interaction with Rtt106p without decreasing the affinity encompassed amino acids 120–303 (Fig. 2a). We measured a *K*_d_ of 0.94 ± 0.06 μM for Bcd1p_120–303_ vs Rtt106p_65–301_, equivalent to the *K*_d_ measured with the full-length Bcd1p for Rtt106p_65-320_ (Fig. 2d left panel). We therefore concluded that the central region of Bcd1p is necessary and sufficient for interaction with the tandem PH domains of Rtt106p. Then, we confirmed the direct effect of Bcd1p on Rtt106p-chromatin association by generating the strain *BCD1*_*1–115*_ harboring an endogenous *BCD1* gene deleted from the coding region necessary for Bcd1p:Rtt106p binding (Supplementary Fig. 2 f). Compared to WT cells, Rtt106p was better associated with chromatin in *BCD1*_*1–115*_ cells even if the effect was milder compared to the effect obtained by inducing transient Bcd1p depletion (Fig. 3b, c).

### NMR solution structure of Bcd1p_120–303_

**Fig. 5:**
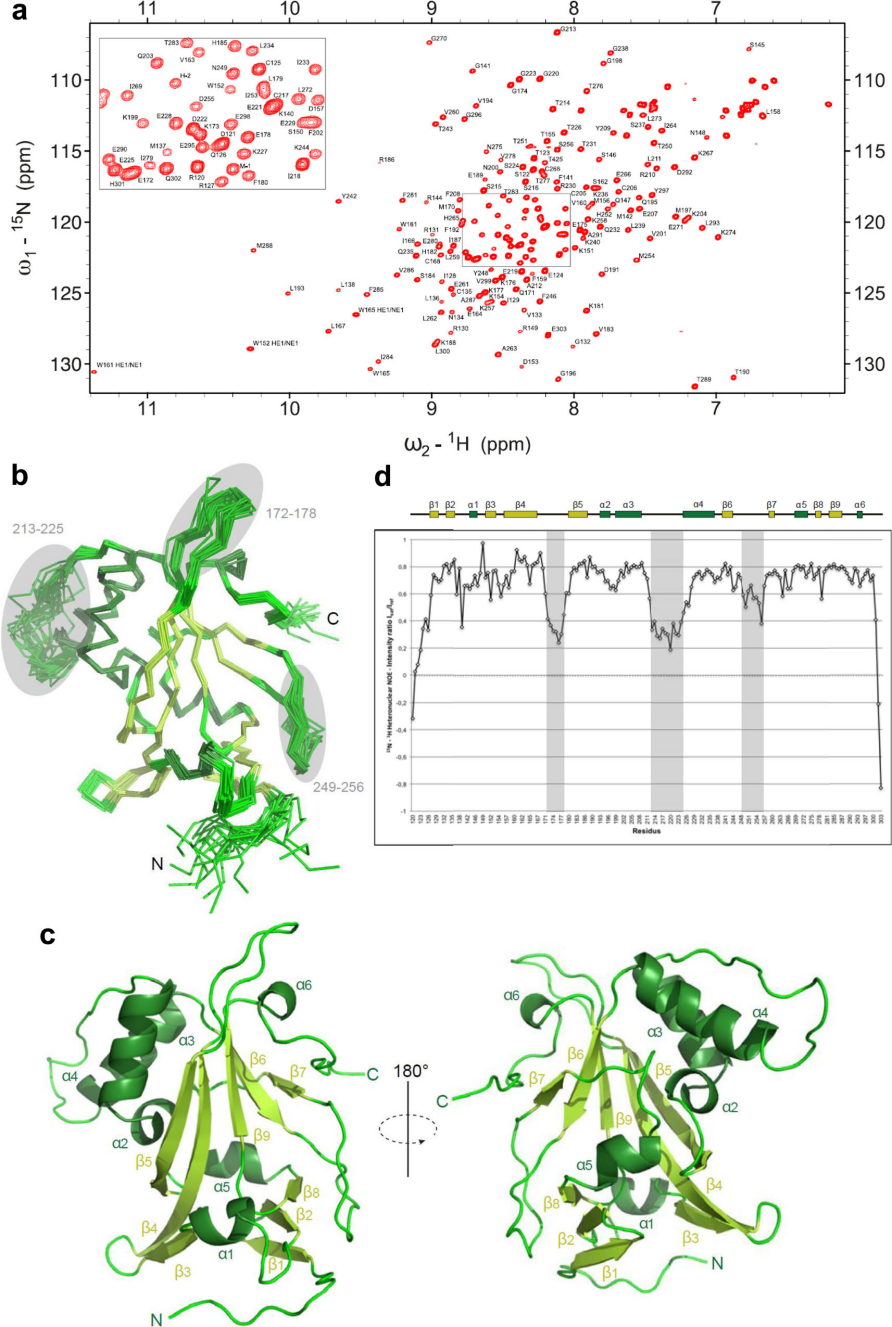
Solution 3D structure of Bcd1p_120-303_. **a** ^1^H-^15^N-HSQC spectra of Bcd1p_120-303_. The assigned peaks are labeled (ω = frequency). The box shows a zoom of the center of the spectra. **b** Ribbon representation of the 20 best NMR solutions for the 3D structure of Bcd1p_120-303_. Flexible loops are circled in gray and labelled. Secondary structure elements are α-helices (in dark green) and β-strands (in light green). N and C are the N-terminal and C-terminal extremities of the protein, respectively. **c** Two opposite views 180° apart in a cartoon representation of Bcd1p_120-303_ with secondary structures labeled and numbered. The color code is the same as in panel **b**. **d** NMR heteronuclear nOe. Residue numbers are indicated at the bottom. Secondary structure elements are represented at the top in the same colors as in panel **b**. The flexible internal regions are highlighted in gray, and reported in panel **b**.

Sequence comparison between the yeast Bcd1 protein and its human homologous ZNHIT6 showed that the central Bcd1p_120–303_ domain resembles the C-terminal region of ZNHIT6 (Supplementary Fig. 6). This observation prompted us to search for additional structural information on this domain in an unbound state. We solved the three-dimensional (3D) structure of Bcd1p_120–303_ using multi-dimensional NMR spectroscopy. This stable Bcd1p subfragment provided well-resolved NMR spectra (Fig. 5a), which enabled the unambiguous assignment of more than 90% of the ^1^H, ^13^C, and ^15^N resonances. NMR data provided a well-defined ensemble of 20 water-refined structures with respective backbone and heavy atom RMSD values of 0.74 ± 0.16 Å and 1.36 ± 0.20 Å for residues 123–301 (Fig. 5b; statistics are detailed in Table 1). The 120–303 domain is composed of nine β-strands and six α-helices, with strands β4–7 and β9 forming a central twisted β-sheet (Fig. 5c and Supplementary Fig. 7). The majority of the helices (α2, α3, α4, and α5) are packed on one side of the central sheet, while on the opposite side, only the small helix α6, the β4–β5 (172–178) and β6–β7 (249–256) loops, and the C-terminal region are present. The N-terminal region Bcd1p_120–149_, whose deletion prevents binding with Rtt106p (Supplementary Fig. 4 and Supplementary Fig. 5), comprises helix α1 and strands β1 and β2. Since deletion of this 120–149 region in Bcd1p results in soluble and folded subfragments (Supplementary Fig. 4 and Supplementary Fig. 5), it could be considered as an independent module, packed on the edge of Bcd1p_150–303_, mainly via a β-sheet involving strands β1, β2, and β8. With the exception of the N- and C-terminal tails of Bcd1p_120–303_, the analysis of the ^1^H–^15^N heteronuclear nOe ratios revealed three flexible regions (172–178, 213–225, and 249–256) related to large loops in the structure (Fig. 5b, d).

**Table 1:** NMR and refinement statistics for protein structures.

	Bcd1p_120–303_
NMR distance and dihedral constraints	
Distance constraints	
Total NOE	4186
Intraresidue	1115
Inter-residue	3071
Sequential (|*i* – *j*| = 1)	1087
Medium range (|*i* – *j*| ≤ 4)	694
Long range (|*i* – *j*| ≥ 5)	1290
Total dihedral angle restraints	280
ϕ	138
ψ	142
Total RDCs	111
Structure statistics	
Violations (mean and s.d.)	
Distance constraints (Å)	0.039 ± 0.036
Dihedral angle constraints (°)	0.889 ± 0.699
Max. distance constraint violation (Å)	0.24 ± 0.04
Max. dihedral angle violation (°)	2.79 ± 0.48
Deviations from idealized geometry	
Bond lengths (Å)	0.0114 ± 0.0003
Bond angles (°)	1.2036 ± 0.0305
Impropers (°)	1.3914 ± 0.0716
Average pairwise r.m.s. deviation^a^ (Å)	
Backbone	0.74 ± 0.16
Heavy	1.36 ± 0.20

^a^Pairwise r.m.s. deviation (residues 123–301) was calculated among 20 refined structures.

### General view of the crystal structure of the Bcd1p_120-303_:Rtt106p_65-301_ heterocomplex

**Fig. 6:**
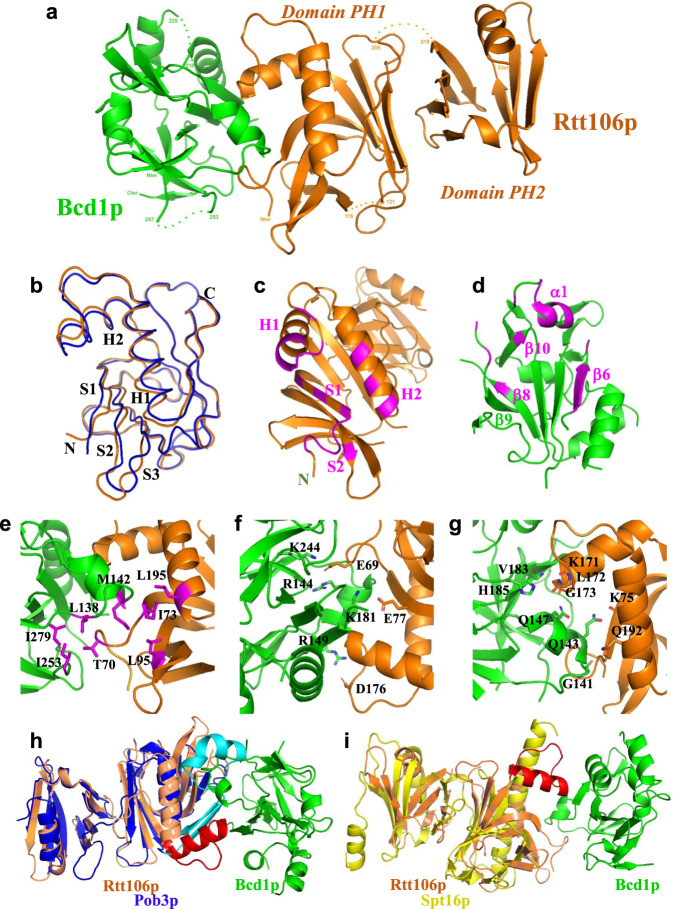
Bcd1p_120-303_ interacts with the PH1 domain of Rtt106p_65-301_. **a** Ribbon representation of the crystal structure of the complex between Rtt106p_65-301_ and Bcd1p_120-303_. Rtt106p is in orange and Bcd1p in green. **b** Superimposition of the PH1 domain of Rtt106p bound to Bcd1p (in orange) and in a free state (in blue, entry PDB code 3TW1^22^ [https://www.rcsb.org/structure/3TW1]). H = Helix, S = Strand, N = N-terminal extremity, C = C-terminal extremity. **c** Residues located at the molecular surface of Rtt106p that are buried upon Bcd1p binding are in magenta. **d** Residues located at the molecular surface of Bcd1p that are buried in the Rtt106p:Bcd1p interface are in magenta. **e–g** Hydrophobic contacts, salt-bridges, and hydrogen bonds at the Rtt106p:Bcd1p interface: **e** Hydrophobic cluster (magenta) at the interface of the heterodimer. **f** Charged residues located at the interface form ionic interactions between Rtt106p and Bcd1p. **g** Network of hydrogen bonds between Rtt106p and Bcd1p. **h, i** Comparison of the 3D structures of Pop3p (in blue, entry PDB code 4PQ0 [https://www.rcsb.org/structure/4PQ0]) and Spt16p (in yellow, entry PDB code 4IOY [https://www.rcsb.org/structure/4IOY]). The sequence spanning amino acids 162–182 in Rtt106p, which differs strongly from Pob3p and Spt16p, is in red.

To understand the mode of interaction of Bcd1p_120-303_ with Rtt106p_65-301_, we characterized the 3D structure of the complex using X-ray crystallography (Fig. 6). The final structure was refined against the dataset collected from a native crystal to an *R*_factor_ of 21.0% and an *R*_free_ of 29.7%, including all reflections between 20 and 2.79 Å resolution (Table 2). The asymmetric unit contains one Bcd1p_120-303_:Rtt106p_65-301_ heterodimer.

**Table 2:** X-ray data collection and refinement statistics.

	Bcd1p_120–303_:Rtt106p_65–301_
Data collection	
Space group	*P*2_1_
Cell dimensions	
* a*, *b*, *c* (Å)	56.71, 66.68, 65.12
α, β, γ (°)	90.00, 104.72, 90.00
Resolution (Å)^a^	50–2.79 (2.79–2.95)
*R*_sym_	4.7 (23.3)
*I* / σ*I*	27.4 (6.7)
Completeness (%)	99.1 (96.7)
Redundancy	6.7 (6.4)
Refinement	
Resolution (Å)	20–2.79
No. reflections	10,224
*R*_work_ / *R*_free_	0.210/0.297
No. atoms	
Protein	3007
Ligand/ion	—
Water	—
*B*-factors	
Protein	58.0
Ligand/ion	—
Water	—
R.m.s. deviations	
Bond lengths (Å)	0.014
Bond angles (°)	1.733

^a^Values in parentheses are for the highest-resolution shell.

The three loops in bound Bcd1p encompassing residues 173–177, 211–224, and 248–252, respectively, were not built due to the lack of density explained by their high flexibility in their native free state (Figs. 5d and 6a). Comparison of the crystal structure of Bcd1p_120–303_ bound to Rtt106p_65-301_ with the NMR structure of Bcd1p_120–303_ in a free state yielded a root-mean-square deviation (RMSD) of Cα positions of 1.13 Å. Indeed, the two 3D structures closely resembled one another and 3D superimposition revealed that no significant conformational modifications of Bcd1p_120–303_ occurred upon binding to Rtt106p_65–301_.

The overall structure of bound Rtt106p_65–301_ displayed the tandem PH domain architecture as previously described^22,26,46^ with a large intramolecular interface between the first (PH1) and second (PH2) domains (Fig. 6a and Supplementary Fig. 7). The loop that connects the β-strands S4 and S5 (residues 119–120) in the PH1 domain and the peptide link between the two PH domains (residues 206–217) were not present in the final crystal structure because of the lack of density and are possibly disordered in their native state. The overall fold of Rtt106p_65-301_ bound to Bcd1p_120-303_ resembles that of Rtt106p_65-301_ in free state^22^ (entry PDB code 3TW1 [https://www.rcsb.org/structure/3TW1]), with a 0.95 Å RMSD for 202 Cα atoms. However, several local conformational changes, with structural deviations that can reach 4 Å, occurred in the PH1 domain upon binding of Bcd1p_120–303_ (Fig. 6b and Supplementary Fig. 8). For instance, some structural modifications occurred in the N-terminal part of the PH1 domain and were identified in the C-terminal part of β-strand S1, in the N-terminal part of β-strand S2 and in the loops connecting β-strands S2 to S3, and S3 to S4. Two major conformational changes were observed in the C-terminal part of the PH1 domain that involve α-helices H1 and H2 as well as the loop connecting these two α-helices. We concluded that the conformational changes induced by complex formation mostly occurred in Rtt106p.

### Bcd1p_120–303_ interacts with the domain PH1 of Rtt106p

The crystal structure of the Bcd1p_120–303_:Rtt106p_65-301_ complex revealed that Rtt106p interacts with Bcd1p only via its PH1 domain (Fig. 6a). Most of the residues from Rtt106p that are buried upon interaction with Bcd1p are located in two distinct parts (Fig. 6c), including residues of the first S1 and S2 β-strands, solvent-exposed residues from the contiguous α-helices H1 and H2, and residues from the loop connecting these two α-helices. For Bcd1p, the interaction interface mainly involved the solvent-exposed faces of helix α1 and of strand β6 located on one edge of the central β-strand (Fig. 6d). This observation reinforces the major role we already highlighted for the region 120–149 of Bcd1p in holding its helix α1 (Supplementary Fig. 4 and Supplementary Fig. 5). In addition, the N-terminal parts of strands β8 and β10 of Bcd1p as well as the loop upstream from strand β9 were partially buried upon binding to Rtt106p_65–301_. A hydrophobic cluster formed at the heterodimer interface with side-chain interactions of four hydrophobic residues from each protein (Fig. 6e). Moreover, an ionic network involved positively charged residues from Bcd1p and negatively charged residues from Rtt106p (Fig. 6f). This observation is in accordance with the electrostatic potential mapped on the molecular surface of Bcd1p_120–303_ (Supplementary Fig. 9). Finally, many hydrogen bonds involving main chains atoms were also observed (Fig. 6g).

### Structural MS characterization in solution of Bcd1p_FL_:Rtt106p-M

**Fig. 7:**
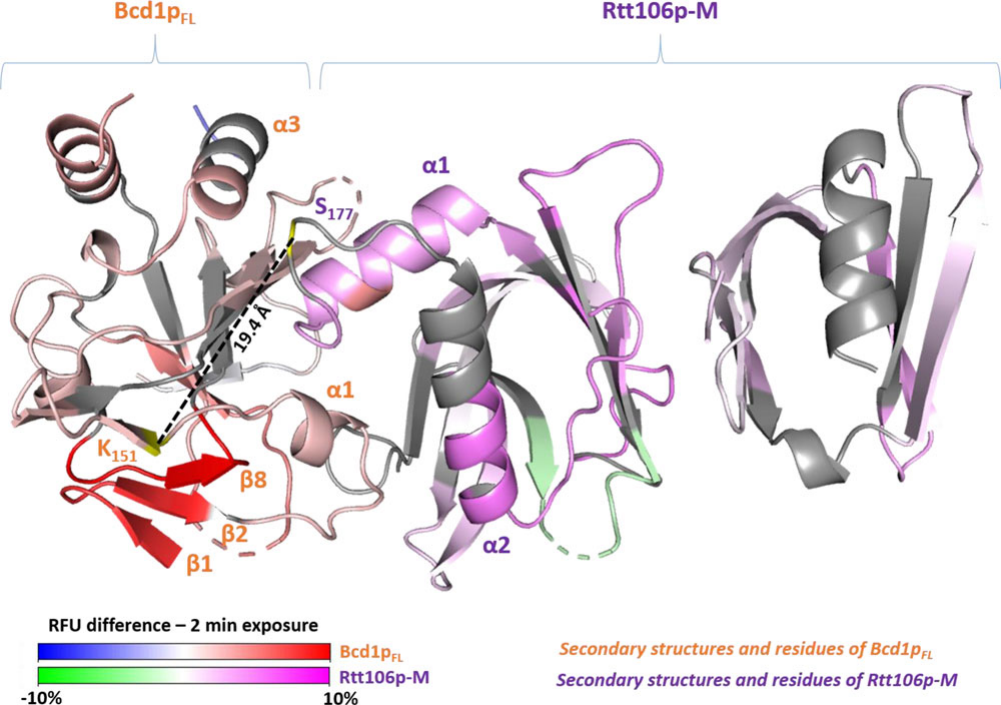
Summary of the XL-MS and HDX-MS experiments. Relative fractional uptake (RFU) differences export on the crystal structure of the complex between Rtt106p-M and Bcd1p_FL_ determined by hydrogen deuterium exchange mass spectrometry (HDX-MS)_._ Export is realized for 2 min deuteration experiments. Differences are color scaled on Bcd1p_FL_ from blue (deprotection) to red (protection) upon Rtt106p-M binding (−10% to 10% RFU difference range). Differences are color scaled on Rtt106p-M from green (deprotection) to magenta (protection) upon Bcd1p_FL_ binding (−10% to 10% RFU difference range). The Cα-Cα distances of intercross-linked peptides are represented with black dotted lines. Orange residues and secondary structures represent the most affected regions on Bcd1p_FL_ while the purple ones represent the most affected regions on Rtt106p-M.

We used alternative approaches to characterize complexes containing the full-length Bcd1 protein (Bcd1p_FL_) based on mass spectrometry. First, we used hydrogen deuterium exchange coupled with mass spectrometry (HDX-MS) to characterize the conformational dynamics of Bcd1_FL_ and Rtt106p-M proteins upon the complex formation. HDX enables identification of regions that either are protected from the solvent upon complex formation or undergo conformational changes resulting in differences in solvent accessibility^47^. We consequently compared the deuterium incorporations of the Bcd1p_FL_:Rtt106p-M complex to those of the isolated partners (Fig. 7). Concerning the impact of Rtt106p-M binding on Bcd1p_FL_, the N-terminal region of Bcd1p_1–125_ was not much affected upon Rtt106p-M binding (Supplementary Fig. 10a and Supplementary Fig. 11). Moreover, Bcd1p showed significant protection upon the complex formation for regions 125–202; 218–234, and 242–281, encompassing respectively, strands β1 to β5 and helices α1–α2; helix α4; helix α5 and strands β7 to β8 (Supplementary Fig. 7a, Supplementary Fig. 10a and Supplementary Fig. 11). While helix α1 was shown to be involved in the interaction (Fig. 6d), the β sheet composed of strands β1, β2, and β8 showed strong protection upon Rtt106p-M binding (Fig. 7, Supplementary Fig. 10a and Supplementary Fig. 11), highlighting a higher stability of this region. Interestingly, region 208–217 (not built in the crystal structure), spanning the C-terminal part of helix α3 and part of the loop α3-α4 showed higher deuterium uptake in presence of Rtt106p-M, highlighting a higher flexibility of this region upon the complex formation. The same analysis was performed on Rtt106p-M and highlighted several regions that were affected upon the complex formation (Fig. 7, Supplementary Fig. 10b and Supplementary Fig. 12). Firstly, regions 75–80 and 94–106 encompassing β-strands S1, the C-terminal part of S2 and S3 (Supplementary Fig. 7b) showed protection upon Bcd1p_FL_ binding. Furthermore, protection was also identified for regions 126–137; 148–175, and 188–233 spanning respectively β-strands S5, S7 and α-helix H1; the C-terminal part of α-helix H2 and β-strands S8 and S9. Interestingly, strands S7, S8, and S9 are at the interface of domains PH1 and PH2. The variations observed in these regions are probably due to the relative intramolecular flexibility between these two domains while strands S1, S2 and helices H1 and H2 were shown to be involved in the interaction with Bcd1p (Fig. 6c).

Next, we performed chemical cross-linking experiments followed by mass spectrometry (XL-MS) to identify potential intercross-linked peptides and assess amino-acid residues of proximity. The resulting peptide fragments were analyzed to look for intermolecular cross-links present as doublets peaks of heavy BS3-d4 and light BS3-d0 modified peptides. Interestingly, a total 11 intramolecular cross-linked peptides of Bcd1p_FL_ and one intermolecular cross-linked peptide involving K_151_ of Bcd1p_FL_ and S_177_ of Rtt106p-M were identified and validated (Fig. 7, Table 3 and Supplementary Table 2). Of note, no intra XL peptides were manually validated for Rtt106p-M, mostly because only the heavy BS3-d4 cross-link peptide was detected in the experiments. K_151_ of Bcd1p_FL_ and S_177_ of Rtt106p-M residues are located at the dimer interface of the complex: while Bcd1p K_151_ residue is part of the strand β3, S_177_ residue of Rtt106p is part of the loop between helices H1 and H2, and neighbor residues directly involved in the ionic and hydrogen bond networks (Fig. 7, Supplementary Fig. 7 and Fig. 6f, g). Thus, HDX and chemical XL-MS results showed remarkable agreement among themselves and with the previous structural characterization of the complex. Besides, HDX allowed to highlight conformational dynamics of the proteins and particularly for Bcd1p, showing a strong impact upon the complex formation for additive region, especially the β1–β2–β8 sheet, which showed a strong protection.

**Table 3:** Cross-linked sites observed with different protein:BS3 (d0/d4) ratios for the complex Bcd1p_FL_:Rtt106p-M.

Cross-linked proteins	Linkage sites	Ratio 1:50	Ratio 1:100	Distance^a^ Cα–Cα (Å)
Bcd1p_FL_–Bcd1p_FL_	K16–K62		√	N.C.
	K16–K88	√	√	N.C.
	K16–S106	√		N.C.
	K16–K109			N.C.
	K16–K151		√	N.C.
	K16–K258	√	√	N.C.
	K62–K80		√	N.C.
	K80–K151	√		N.C.
	K87–K151	√		N.C.
	K140–K151	√		15.4
	K188–K151	√	√	10.1
Rtt106p-M–Bcd1p_FL_	S177–K151	√	√	19.4

Identified cross-linked sites are represented for all tested ratios (technical duplicates).

^a^Cα–Cα distances are indicated according to the crystal structure (N.C. for “not crystallized” mention is indicated for cross-links involving at least one site that has not been crystallized).

### 3D model of a potential Bcd1p:Rtt106p:(H3:H4)_2_ complex

Deposition of newly synthesized H3:H4 complex on replicating DNA relies on the interaction of Rtt106p with histone H3:H4 tetramers and requires acetylation of H3 lysine 56^22^. To explore whether Bcd1p could interfere in the formation of the Rtt106p:(H3:H4)_2_ complex, we built a 3D model of the heterodimer Rtt106p_65–301_:Bcd1p_120–303_ bound to the histone tetramer (H3:H4)_2_, on the basis of the 3D model established for Rtt106p:(H3:H4)_2_^22^ and the present crystal structure of Rtt106p_65–301_:Bcd1p_120–303_ (Supplementary Fig. 13a). In this model, the binding of Bcd1p_120–303_ appears to be compatible with the structure of Rtt106p:(H3:H4)_2_ complex as histones H3:H4 and Bcd1p have distinct interaction regions on Rtt106p. We also performed ITC measurements with an acetylated peptide (H3K56ac) known to interact with the PH2 domain^22^. Our data showed similar interaction modes between H3K56ac and the free form of Rtt106p_65–301_ or the form bound to Bcd1p_120–303_ (Supplementary Fig. 13b). Affinities and thermodynamic values in both experiments were of the same order of magnitude. We concluded that Rtt106p_65–301_ can simultaneously interact with Bcd1p_120–303_ and the H3K56ac peptide.

## Discussion

Here, we report on a link between the machinery involved in box C/D snoRNP assembly and the machinery for chromatin assembly and remodeling. Genetic interaction mapping (GIM) analysis performed with the query mutation strain disrupted for the snoRNP assembly factor Rsa1p revealed an epistatic effect with several genes involved in chromatin remodeling, including histone chaperones (Fig. 1). We observed that one of these histone chaperones—Rtt106p—directly binds Bcd1p (Fig. 2), another RNP assembly factor that is crucial for box C/D snoRNP assembly and cell growth. The selective interaction of Rtt106p with Bcd1p relies on a structural motif absent in other structurally related histone chaperones Pob3p and Spt16p^22,26,39,40^ (Fig. 6h, i). No clear homolog of Rtt106p has been identified in human, but the Rtt106p binding domain (RBD, Fig. 2a) in Bcd1p is conserved in human and mouse^13^ (Supplementary Fig. 6a), and in other yeasts including pathogenic *Candida* species (Supplementary Fig. 6b). Our structural studies revealed the 3D structure of this interaction domain spanning amino acids 120–303 (Fig. 5b and Fig. 6a). Structural MS analysis and our X-ray structure of Bcd1p_120-303_:Rtt106p_65-301_ indicated that the region spanning amino acids 120–149 drives the interaction with the PH1 domain of Rtt106p. Using the Dali server ([http://ekhidna.biocenter.helsinki.fi/dali_server]), the wheel domain of the protein Cns1 was retrieved as the best 3D homolog of Bcd1p_120–303_. This result confirms recent predictions about the presence of a wheel domain in Bcd1p^13^. When aligned, these two homologs showed structural similarities, especially in the organization of the β-strands, despite low amino-acid sequence conservation (Supplementary Fig. 14). Interestingly, region 150–303 in Bcd1p, which encompasses the core β-sheet formed with strands β3–β7 and β9, preferentially aligns with the wheel domain of Cns1p, especially with strands β2 to β6. Cns1p has been described as a co-chaperone of Hsp90, assisting the folding of the elongation factor eEF2^48^. One can thus assume that Bcd1p adopts similar functions via its region 150–303, which is mainly free of interactions with Rtt106p.

In agreement with data resulting from the GIM analysis, it has already been suggested that Rsa1p is connected with chromatin dynamics. Rsa1p and its binding partner Hit1p were shown to contribute to condensin Brn1p accessibility to rDNA and to delay rDNA compaction during the cell cycle, thereby enabling the coordination of nucleolar segregation with mitotic exit^49^. The mechanism of action of Rsa1p:Hit1p in the control of rDNA compaction is not known, but the role of snoRNP assembly factors associated with chromatin regulation nevertheless appears to be conserved. Indeed, mitotic exit in Schwan cells involves ZNHIT3, the counterpart of Hit1p in human^50^. Since *rsa1*Δ leads to chromatin hypercondensation^49^, it is possible that mutations that affect actors of DNA condensation compensate for the negative effect of *rsa1*Δ and increase the fitness of mutant cells, as selected in the GIM approach. We thus favor the hypothesis of a suppressive effect when *rsa1*Δ is combined with the disruption of *RTT106* (*rtt106*Δ) or with the disruption of genes encoding components of the chromatin remodeling complexes HIR, INO80, and CAF-1.

At first glance, the characterization of a Bcd1p:Rtt106p complex suggested that Rtt106p could have a function in snoRNP assembly, and/or that Bcd1p regulates the activity of Rtt106p in the control of chromatin structure, and/or that the Bcd1p:Rtt106p complex contributes to another cellular mechanism. The present work did not reveal any strong involvement of Rtt106p in the function of Bcd1p in snoRNP biogenesis under standard laboratory culture conditions. Indeed, under these conditions, the disruption of *RTT106* (Supplementary Fig. 2b) had no major effect on the steady-state levels of snoRNAs. We previously identified the N-terminal region as necessary for Bcd1p function and it did not correspond to the region involved in Rtt106p interaction^12^. Nonetheless, we show that the histone chaperone Rtt106p is present on box C/D snoRNAs including pre-snoRNAs (Fig. 3a) and remarkably also at snoRNA gene loci (Fig. 3b). The specificity of the association of Rtt106p with pre-snoRNAs was confirmed by the observation that it requires the presence of Bcd1p and the Snu13p-interacting protein Rsa1^51^ (Fig. 3a). Hence, Rtt106p is likely recruited to snoRNAs by binding Bcd1p, which is either present in a pre-existing pre-snoRNA:Snu13p:Rsa1p:Bcd1p RNPs or in a protein-only complex before its loading to RNA. However, in both scenarios, binding could occur during preparation of the cell extract thereby explaining why no contribution of Rtt106p to the biogenesis of snoRNPs was observed. Nonetheless, it is possible that under specific growth conditions, association of Rtt106p with pre-snoRNP contributes to or regulates snoRNP biogenesis.

Can Bcd1p regulate Rtt106p activity? Rtt106p contributes to the formation of nucleosome on replicating DNA^22–26^ but is also linked to transcription activity^29^. During the replication-dependent process, new (H3:H4) dimers present in a nuclear heterotrimeric complex Asf1p:(H3:H4) are transferred to CAF-1 and Rtt106p for formation and deposition of (H3:H4)_2_ tetramers onto newly synthesized DNA^52^. Less information is available on the mode of action of Rtt106p for nucleosome disassembly associated with transcription initiation and nucleosome reassembly in the wake of RNA polymerase compared to the documented activities of FACT, Rtt109p, and Asf1p in such processes^45,53^. Rtt106p interacts functionally and genetically with various regulators of RNA polymerase II transcription^43,54–56^ and is physically associated with transcribed chromatin regions^29^. Our ChIP analysis confirmed enrichment of Rtt106p in the body of active genes including the U3 snoRNA locus (*SNR17A*, Fig. 3b, c). Remarkably, a co-immunoprecipitation assay revealed a robust association between Rtt106p and Rpb1p, the largest subunit of RNA polymerase II (Fig. 4a). This observation suggests that enrichment of Rtt106p to these loci is RNA polymerase II-dependent and thus identifies histone chaperone Rtt106p as an actor involved in chromatin control during transcription, as primarily proposed^29^. Whether Rtt106p provides H3:H4 for nucleosome assembly by traveling with elongating RNA polymerase II complexes or afterwards is still an open question. The identification of the Rtt106p:Rpb1p interaction favors the first hypothesis.

Most importantly, the present data reveal that Bcd1p reduces the association between Rtt106p and the RNA polymerase II as well as the enrichment of H3K56ac marks to several transcriptionally active genes (Fig. 4). Therefore, the data strongly suggest that, by direct interaction with Bcd1p, Rtt106p dissociates from the RNA polymerase machinery that allows its recruitment and function at actively transcribed genes.

We propose that the control of Rtt106p activity by Bcd1p is not performed on chromatin, at the site of transcription. Originally, it was suggested that Rtt106p must interact with DNA to deliver histones H3:H4 during replication^30^ and a conserved positively charged surface of Rtt106p was shown to be responsible for dsDNA binding^30,46^. First, in the crystal Bcd1p:Rtt106p complex (Fig. 6), the binding site for dsDNA at the surface of Rtt106p is still exposed to the solvent and a second positive patch continuing the one of Rtt106p is present on the surface of Bcd1p (Supplementary Fig. 15). In addition, the 3D model of the heterodimer Rtt106p_65–301_:Bcd1p_120–303_ bound to the histone tetramer (H3:H4)_2_ (Supplementary Fig. 13a) also predicts absence of direct interference of Bcd1p in Rtt106p activity for the nucleosome assembly. Second, we observed the same positive effect of Bcd1p depletion on Rtt106p enrichment at transcribed loci where Bcd1p was not enriched (Fig. 3b and Supplementary Fig. 2e). Indeed, the enrichment of Bcd1p to snoRNA loci observed in the ChIP experiment corresponds to the presence of Bcd1p on nascent pre-snoRNAs, as evidenced by the loss of enrichment upon RNase treatment^12^. Interestingly, we found that the association of Rtt106p at chromatin is partially sensitive to RNase treatment at both transcribed and nontranscribed loci (Supplementary Fig. 2e); the underlaying mechanism is unknown but we determined that it was independent from the effect generated by Bcd1p. It remains to be determined how the interaction of Bcd1p precludes the transcription-dependent recruitment of Rtt106p. We propose that Bcd1p could deplete Rtt106p from active loci by forming delocalized Bcd1p:Rtt106p complexes. Concerning the snoRNA loci, an attractive possibility would be that the interaction of Rtt106p with the nascent pre-snoRNPs containing Bcd1p could reduce the activity of Rtt106p during transcription at these loci. However, it is likely the free RNA-unbound form of the Rtt106p:Bcd1p complex that has a regulatory function and not the one associated with nascent pre-snoRNPs. Indeed, the modulation of Rtt106p association across the *SNR17A* gene encoding U3 is Rsa1p-independent and consequently does not rely on the formation of the pre-snoRNP (Fig. 3b).

In conclusion, the present data reinforce the proposal that, in addition to its activity during replication, the chaperone Rtt106p contributes to chromatin structure during transcription elongation^29^. We identified a new interaction interface in its PH1 domain that binds Bcd1p, an essential factor for C/D box assembly, and therefore for functional ribosome formation and cell proliferation^10–13^. The interaction between Rtt106p and Bcd1p we evidenced here may represent an important connection point to coordinate RNA polymerase II transcription activity and ribosome formation.

## Methods

### Reagents

Ammonium acetate (NH_4_Ac), 4-(2-Hydroxyethyl)piperazine-1-ethanesulfonic acid (HEPES), tris(2-carboxyethyl)phosphine (TCEP), dibasic potassium phosphate (K_2_HPO_4_), monobasic potassium phosphate (KH_2_PO_4_), guanidine hydrochloride (GuHCl), and hydrochloric acid (HCl) were purchased from Sigma (St. Louis, MO, USA). Deuterium oxide (D_2_O) and deuterium chloride were purchased from Euriso-top (Saarbrücken, Germany). Bis(sulfosuccinimidyl) suberate d0/d4 (BS3-d0/d4) and Zeba column were purchased from Thermo Scientific (Rockford, IL, USA). Vivaspin cutoff 5 kDa was purchased from Sartorius (Goettingen, Germany), Glu-fibrinogen peptide (GFP) from ERA (Golden, CO, USA), trypsin from Promega (Madison, WI, USA), and pepsin-immobilized cartridge from Applied Biosystems (Forster city, CA, USA). Protein samples were home produced. Oligonucleotides are listed in Supplementary Table 3.

### Plasmids and strains

Plasmids are listed in Supplementary Table 4. The fragments of proteins Bcd1 and Rtt106 used for biochemical and structural assays were overexpressed in *E. coli* BL21 pRARE2 grown in LB rich media and transformed with recombinant pnEA-3CH plasmid leading to expression of a 6xHis-tag at the N-terminal extremity of the protein followed by the PreScission protease cleavage site^57^. In addition, for the co-expression assays, cells were transformed by the recombinant plasmid pnCS^57^.

All yeast strains were generated in the *S. cerevisiae* BY4741 background unless otherwise indicated (Supplementary Table 5). Genes were deleted by one-step integration of KO cassettes, followed by PCR verification of the 5’ and 3’ ends of targeted gene. *GAL1* promoter or TAP tag was inserted by homologous recombination. Correct insertion at the locus was checked by PCR and sequencing.

### Yeast two-hybrid assays

pGBKT7 plasmids expressing the bait protein fused to the DNA binding domain of Gal4 and pACTII expressing the prey protein fused to the transcription activation domain of Gal4 were used to transform haploid yeast cells Y187 and Y190, respectively. The transformed cells were selected on single selective medium lacking tryptophan (Trp^–^) for pGBKT7 and leucine (Leu^–^) for pACTII. After mating, the diploid cells containing both plasmids were selected on double selective medium Leu^–^Trp^–^ and then plated on triple selective medium Leu^–^Trp^–^His^–^ in order to reveal expression of the reporter *HIS3* gene. Increase concentration of 3-amino-1, 2, 4-triazol (3-AT) (Sigma), a competitive inhibitor of the product of *HIS3*, was used to evaluate the strength of the interaction between the bait and the prey proteins. Growth was assessed after three days of incubation at 30 °C.

### Genetic interactions mapping (GIM)

The method is described in detail elsewhere^58,59^. Here we describe the main principles of the different steps of the method. The query strain *rsa1*Δ*::prMFα2Nat*^*R*^ was generated by changing the *KanMX4* marker to the prMFα2Nat^R^ marker in the knockout mutant *Matα* strain BY4742 *rsa1*Δ*::KanMX4*. The query strain was transformed with plasmid pG1D1 bearing hygromycin resistance and mated in mass with the pooled *MATa* yeast deletion library (Kan^R^) comprising 4885 *S. cerevisiae* mutants from the systematic collection of haploid deletion strains and 977 barcoded haploid DAmP (decreased abundance by mRNA perturbation) strains in which the function of essential genes was perturbed by the introduction of a drug resistance cassette downstream from the stop codon, leading to an extended 3’ UTR^32^. The diploids were selected for hygromycin and kanamycin resistance. After sporulation, the *Matα* double mutant haploids cells were directly selected for combined nourseothricin and kanamycin resistance in standard rich liquid medium (YPD) and grown at constant turbidity for 18 generations. The relative measured fitness of the double mutants log2(Q/R) was estimated from the values of the intensity of hybridization signal on custom glass slide oligonucleotide microarrays (custom Agilent, GEO GPL18088) for the query double mutant population (Q) compared with a reference double mutant population obtained in parallel (R). Hybridizations were performed with fragments obtained by amplification of the tags by PCR with Cy3 and Cy5 5’-end-labeled oligonucleotides.

### Protein co-immunoprecipitation (co-IP) and RNA immunoprecipitation (RIP) assays

Yeast cells were grown at 30 °C in YPD to A_600_ ~0.8–1. After centrifugation, cell pellets were resuspended in breaking buffer (HEPES-KOH 20 mM (pH 7.9); NaCl 150 mM; MgCl_2_ 3 mM; DTT 0.5 mM, TRITON-X-100 0.1%; Glycerol 10%; Antiproteases 1× (Thermo Scientific) and lysed by bead-beating. The lysate was then clarified twice by centrifugation at 6,000 × g for 5 min. The yeast cell extract was incubated, depending on the tagged protein, either with protein G magnetic beads (Invitrogen) coupled with anti-HA 3F10 antibodies (Roche) at dilution 1/40, or with IgG-Sepharose beads (GE Healthcare), or with ANTI FLAG-M2 agarose beads (Sigma) for 2 h at 4 °C. The beads were washed three times in breaking buffer. Proteins retained on beads were extracted by boiling in 1× Laemmli buffer (2% SDS, 10% glycerol, 5% 2-mercaptoethanol, 0.01% bromophenol blue and 60 mM Tris-HCl, pH 6.8), fractionated on 12.5% SDS-PAGE and analyzed by Western blotting according to standard procedures using rabbit commercial Peroxidase Anti-Peroxidase (PAP) at dilution 1/2000 or monoclonal anti-HA antibodies (Roche) at dilution 1/250 and ECL Prime Western blotting system (GE healthcare). Anti-Dps1p (provided by C. Allmang and G. Eriani, IBMC, Strasbourg, France) was used at 1/5000 dilution to reveal loading controls. For RIP experiments, RNAs retained on beads were extracted with phenol-chloroform-isoamyl alcohol and analyzed by RT-qPCR. cDNAs were generated using M-MLV Reverse Transcriptase (Invitrogen) and random hexamers or oligonucleotide primers specific of pre-snoRNAs. Quantitative PCR using iTaq Universal SYBRGreen premix (Biorad) were performed on the STEPONE apparatus using a relative quantification and a standard curve method.

### Chromatin immunoprecipitation (ChIP)

Yeast cells grown as described above to the mi-log phase were fixed with 1% formaldehyde (10 min at room temperature), quenched with 0.125 M glycine, and lysed by bead-beating in breaking buffer (see previous paragraph) and presence of 400 µL glass beads (Sigma). Chromatin was sonicated to an average length of 200–500 bp and incubated on a wheel at 4 °C during 60 min in solubilization buffer (50 mM HEPES-KOH (pH 7.5), 500 mM NaCl, 1 mM EDTA, 1% Triton X-100, 0.1% Na-deoxycholate, 0.1% SDS, Antiproteases 1× (Thermo Scientific)). FLAG-M2 Agarose FLAG-beads or protein G magnetic beads (Invitrogen) coupled with H3K56ac antibody (active motif) at dilution 1/50 for IP and 1/1000 for blotting were pre-cleared in breaking buffer containing 20 μg/mL BSA for 2 h. Protein-DNA complexes were captured on beads for 2 h at 4 °C, washed twice with low salt buffer (50 mM HEPES-KOH (pH 7.5), 50 mM NaCl, 1 mM EDTA, 0.1% Triton X-100, 0.01% Na-deoxycholate, 0.05% SDS), followed by washes with LiCl buffer (250 mM LiCl, 10 mM Tris-HCl (pH 8), 1 mM EDTA, 0.01% Igepal, 0.05% Na-deoxycholate) and IPP150 buffer (10 mM Tris-HCl (pH 8), 15 mM NaCl, 0.01% Igepal). After digestion with proteinase K (20 µg/µL in IPP50 buffer), reversal of the cross-links by overnight incubation at 70 °C and elution, DNA was purified and quantified by qPCR using gene-specific primers. The ratio of DNA in the immunoprecipitated material to DNA in the input chromatin was calculated and normalized to control reactions made with strains that do not express tagged proteins or with protein G beads coupled with IgG antibodies.

### Statistics and quantitative analyses

Data are reported as mean values plus standard error of the mean of a least three biological replicates. The significance level using a paired two-tailed Student’s *t*-test was set to *P* values *P < 0.05, **P < 0.01, and ***P < 0.001.

### Expression and purification of recombinant proteins

Co-expression tests were carried out using the procedure previously described^51^. Briefly, the *E. coli* BL21(DE3) cells supplemented with the pRARE2 plasmid were co-transformed with pnEA-3CH and pnCS plasmids carrying the sequences of interest. Growth was performed in 100 mL LB medium overnight at 20 °C after induction with 0.2 mM IPTG (Isopropyl β-D-1-thiogalactopyranoside) when OD_600_ reaches 0.7. Then, the cells were harvested by centrifugation, resuspended in 4 mL high salt buffer (25 mM HEPES pH 7.5, 400 mM NaCl, 10 mM Imidazole) and lysed by sonication. The lysate was centrifuged 30 min at 4 °C at 16,100 × *g*. 250 μL of 50% slurry beads (TALON Superflow, GE Healthcare) were added to the supernatant and incubated for 1 h at 4 °C. After centrifugation 5 min at 700 × *g*, the supernatant was discarded, and the beads were washed three times with 500 μL of high salt buffer. Finally, a sample of the beads was mixed with 2× blue denaturing loading buffer to be analyzed by SDS-PAGE.

For preparation of protein for NMR experiment, bacteria transformed with expression vectors were grown at 37 °C under agitation until 0.6 < OD_600nm_ < 1.0 and protein overexpression was induced with 0.3 mM of IPTG (Isopropyl β-D-1-thiogalactopyranoside) at 20 °C overnight. The protein Bcd1_120–303_ was produced with non-deuterated or 100% deuterated M9 minimum medium containing [^13^C]-glucose (2 g/L) and [^15^N]-NH_4_Cl (1 g/L) as nutrient sources. Cells were lysed by sonication in buffer containing 25 mM HEPES (pH 7.5), 300 mM NaCl, 10 mM Imidazole, and 0.5 mM TCEP (Tris(2-CarboxyEthyl)Phosphine). After binding on TALON resin (Ozyme), proteins were eluted using 300 mM of Imidazole and dialyzed at 4 °C in buffer 20 mM HEPES (pH 7.5), 300 mM NaCl, and 1 mM DTT with PreScission protease to remove the 6xHis-tag.

For crystallization assays, Bcd1_120–303_ and Rtt106_65–301_ proteins were overexpressed separately and then mixed at a ratio of 1.2:1. The complex between the two partners was isolated from Bcd1p monomer by gel filtration on HiLoad 16/60 Superdex 200 (GE Healthcare) using an AKTA FPLC system (GE Healthcare) in buffer containing 10 mM Tris (pH 7.5) and 150 mM NaCl. The complex was concentrated to ~10 mg/mL using a 10 kDa Amicon® device (Millipore).

Preparation of Bcd1p_120–303_ for analysis by NMR included a step of gel filtration using a HiLoad 16/60 Superdex 75 (GE Healthcare) in a buffer containing 10 mM phosphate (pH 6.4), 150 mM NaCl and 0.5 mM TCEP. The protein was concentrated to ~1 mM using Amicon® device (Millipore). Finally, 10% D_2_O (v/v) was added to the sample before the NMR experiments.

### 1D-SDS-PAGE followed by nanoLC-MS/MS

Gel bands of interest were excised and processed as follows for protein content identification.

### Protein preparation for liquid digestion

The gel pieces were successively washed with 50 μL of 25 mM NH_4_HCO_3_ and 50 μL of acetonitrile (three times), dehydrated with 100 μL of acetonitrile before reduction in the presence of 10 mM DTT in 25 mM NH_4_HCO_3_ (1 h at 57 °C), and alkylated in the presence of 55 mM iodoacetamide in 25 mM NH_4_HCO_3_. For tryptic digestion, the gel pieces were resuspended in 2 volumes of trypsin (12.5 ng/μL; Promega V5111) freshly diluted in 25 mM NH_4_HCO_3_ and incubated overnight at 37 °C. The digested peptides were then extracted from the gel in a buffer containing 34.9% H_2_O, 65% acetonitrile, and 0.1% HCOOH. The acetonitrile was removed by evaporation and peptides analyzed by nano LC-MS/MS.

### Chromatography conditions on NanoAcquity

The analysis was performed on a nanoACQUITY Ultra-Performance-LC (UPLC, Waters Corporation, Milford, USA). The samples were trapped on a 20 × 0.18 mm, 5 µm Symmetry C18 precolumn (Waters Corporation, Milford, USA), and the peptides were separated on a ACQUITY UPLC^®^ BEH130 C18 column (Waters Corporation, Milford, USA), 75 µm × 250 mm, 1.7 µm particle size. The solvent system consisted of 0.1% formic acid in water (solvent A) and 0.1% formic acid in acetonitrile (solvent B). Trapping was performed during 3 min at 5 µL/min with 97% of solvent A and 3% of solvent B. Elution was performed at a flow rate of 300 nL/min, using 3–40% gradient (solvent B) over 79 min followed by 80% (solvent B) over 10 min at 60 °C.

### MS and MS/MS conditions on TripleTOF 5600 mass spectrometer

The MS and MS/MS analyzes were performed on the TripleTOF 5600 an hybrid quadrupole orthogonal acceleration time-of-flight tandem mass spectrometer (ABSciex). The mass spectrometer was operated in positive mode, with the following settings: ion spray voltage floating (ISVF) 2300 V, curtain gas (CUR) 25 psi, interface heater temperature (IHT) 75 °C, ion source gas 1 (GS1) 2 psi, declustering potential (DP) 100 V. Information-dependent acquisition (IDA) mode was used with top 5 MS/MS scans. The MS scan had an accumulation time of 250 ms on *m/z* [400–1250] range and the MS/MS scans 100 ms *m/z* [150–1600] range in high sensitivity mode. Switching criteria were set to ions with charge state of 2–4 and an abundance threshold of more than 150 counts, exclusion time was set at 12 s. IDA rolling collision energy script was used for automatically adapting the CE. Mass calibration of the analyser was achieved using peptides from digested BSA. The complete system was fully controlled by AnalystTF 1.6 (AB Sciex).

### MS/MS data interpretation

The peak list has been searched against a SwissProt database (created 2020-12-15, containing *Escherichia coli* target sequences and the sequences of the recombinant proteins, using Mascot (version 2.6.2, Matrix science, London, England). The database, which contained sequences of human proteins including common contaminants (human keratins) and porcine trypsin, was created using an in-house database generation toolbox^60^. During database search, up to one missed cleavage by trypsin, one fixed modification carbamidomethylation of Cysteine (+57 Da) and one variable modification oxidation of Methionine (+16 Da), were considered. The peptide mass tolerance (tolerance of mass measurement for precursor ion) was set to 15 ppm and the MS/MS mass tolerance (tolerance of mass measurement for fragment ion) set to 0.05 Da. Proline pipeline was used to validate the identification results. The protein identification validation parameters were set as follows: a peptide with a minimal length of seven amino acid, and an ion score ≥25.

### NMR experiments and calculation of Bcd1p_120–303_ structure

The almost complete resonance assignment of ^13^C/^15^N-labeled Bcd1p_120–303_ was achieved using a standard approach based on 3D NMR spectra recorded at 303 K using 600 MHz and 950 MHz (TGIR, Gif-sur-Yvette) Bruker Avance III spectrometers, both equipped with cryoprobes. Initial NMR structures were calculated using the automated procedure of CYANA 3.97^61^ using dihedral angle restraints derived from TALOS-N^62^ and distance restraints derived from 3D ^1^H–^13^C and ^1^H-^15^N-NOESY-HSQC spectra. The nOe assignments were carefully checked after the final iteration. Using the AMPS-NMR web portal^63^ [http://pyenmr.cerm.unifi.it/access/index/amps-nmr], 100 CYANA structures were refined in water against ^1^H–^15^N RDC restraints measured with the help of Best-TROSY and semi Best-TROSY experiments recorded at 303 K and 600 MHz in pf1-containing NMR buffer. The 20 structures with the lowest energies were selected as the most representative. In these structures, 99.3% of residues were favored to generously allowed regions of Ramachandran space and 0.7% were outside. Dynamics of the backbone were evaluated through measurement of the ^1^H–^15^N heteronuclear nOe ratios recorded in the same conditions as the ones used for assignment. Figures were prepared with PyMOL 2.2.0.

### Isothermal Titration Calorimetry

Interaction experiments between various fragment of Bcd1p and Rtt106p were recorded at 293 K in buffer containing 10 mM NaPi (pH 7.5), 150 mM NaCl and 0.5 mM TCEP using an iTC200 microcalorimeter (GE Healthcare). Calorimetric data were analyzed with Origin7 software. Proteins were used at concentrations of 40 μM for the titrated and 400 μM for the titrant. Interaction experiments between Rtt106p_65–301_ (with or without Bcd1p_120–303_) and H3K56ac (IRRFQKacSTELL) peptide (Proteogenix) were performed at 200 μM for the protein(s) and 2 mM for the peptide as the titrant.

### Crystallization and collection of X-ray data

Crystals from the complex formed between Rtt106p_65–301_ and Bcd1p_120–303_ were grown by vapor diffusion in hanging drops. The drops were made at 293 K by mixing 2 μL of the protein solution at 10 mg/mL and 4 μL of a reservoir solution containing 10% (w/v) PEG 8 K, 20% (v/v) ethylene glycol, 10 mM 1,6-hexanediol, 10 mM 2-propanol, and 100 mM sodium HEPES at pH 7.5. These crystals belonged to space group *P*2_1_ with unit-cell parameters *a* = 56.7 Å, *b* = 66.7 Å, *c* = 65.1 Å, and *β* = 104.7° (Table 2). Assuming one heterodimer in the asymmetric unit, the packing density *V*_M_ was 2.46 Å^3^.Da^−1^ and the solvent content was 50.1%. Crystals were flash frozen in liquid nitrogen in the mother liquor with addition of 25% glycerol as cryoprotectant. A native dataset at 2.79 Å resolution was collected at 100 K on beamline ID29 at the European Synchrotron Radiation Facility (ESRF, Grenoble), with incident radiation at a wavelength of 1.033 Å and a crystal-to-detector distance of 405 mm. Diffraction spots were recorded on a Pilatus 6M-F detector with 0.1° oscillation and 0.04 s exposure per image. Data were indexed and scaled using XDS^64^ and indexed intensities were converted to structure factors using TRUNCATE in the CCP4 suite^65^ without any σ cutoff.

### Crystal structure determination

The crystal structure of the Rtt106p_65–301_:Bcd1p_120-303_ complex was solved by molecular replacement with the program PHASER^66^ using the crystal structure of Rtt106p^22^ (entry PDB code 3TW1 [https://www.rcsb.org/structure/3TW1]) and the NMR structure of Bcd1p_120-303_ (entry PDB code 6NZ2 [https://www.rcsb.org/structure/6NZ2]). A single solution was obtained with LLG = 508 and TFZ = 26.0. The model was built using COOT^67^, and the crystal structure was refined in the range 20–2.79 Å using REFMAC5^68^. Ten percent of the native data were selected for *R*_free_ calculations. The model was refined to the final *R*_factor_ of 21.0% and *R*_free_ of 29.7% (Table 2) and includes residues 66–118, 121–205, and 218–298 of Rtt106p, residues 128–172, 178–210, 225–247, and 253–301 of Bcd1p. Coordinates of the Rtt106p_65–301_:Bcd1p_120–303_ structure have been deposited in the Protein Data Bank (entry PDB code 6THL [https://www.rcsb.org/structure/6THL]). Over 92% of the residues were within the most favored regions in a Ramachandran plot, as defined by PROCHECK^69^. Figures were prepared using PyMOL 2.2.0 software.

### Nondenaturing Mass Spectrometry (MS)

For native MS experiments, individual partners and co-purified Bcd1p_FL_:Rtt106p_65–320_ complex were buffer exchanged against 150 mM ammonium acetate buffer, pH 7.5 using Zeba microcentrifuge gel filtration columns (2 cycles). Protein concentrations were determined by UV absorbance using a NanoDrop 2000 spectrophotometer (Thermo Fisher Scientific, France). Mass spectrometry experiments were carried out on a hybrid electrospray quadrupole time-of-flight mass spectrometer (Synapt G2 HDMS, Waters, Manchester, UK) equipped with an automated chip-based nanoelectrospray source (Triversa Nanomate, Advion Biosciences, Ithaca, USA) operating in the positive ion mode. Denatured MS analysis was performed on the Synapt G2 HDMS instrument with external calibration using the multiply charged ions produced by 2 µM horse heart myoglobin solution diluted in water/acetonitrile/formic acid (50 v/50 v/1 v) and standard interface tuning parameters of the mass spectrometer (Vc, 40 V; Pi, 2.1 mbar). For native MS experiments, external calibration was performed using singly charged ions produced by a 2 mg/mL solution of cesium iodide in 2-propanol/water (1 v/1 v). Instrumental parameters were carefully optimized to improve desolvation and ion transfer as well as to maintain noncovalent interactions. In particular, the pressure during the first pumping stage was increased to 6 mBar using a throttling valve and the sample cone voltage Vc was set to 120 and 200 V. Native MS data were interpreted using MassLynx 4.1 (Waters, Manchester, UK).

### Ion mobility—Mass spectrometry

Samples were prepared for IM-MS experiments as described above for nondenaturing MS experiments, and the analyses were conducted on the same above-mentioned mass spectrometer. IM parameters were carefully optimized in order to maintain noncovalent complex with the best desolvation possible without ion activation before entering the ion mobility cell. Instrument parameters were: capillary voltage, 1.7 kV; cone voltage, 100 V; trap bias, 44.5 V; trap CE, 4 V; transfer CE, 2 V; wave velocity 1053 m/s; wave height, 40 V; backing pressure, 6 mbar; He flow rate, 105 mL/min; N2 flow rate, 25 mL/min. The ion ^TW^CCS_N2_ was determined from external calibration with native ions of known ^DT^CCS_He_, i.e., β-lactoglobulin, transthyretin and bovine serum albumin^42^. IM-MS were acquired in triplicate using identical IM parameters, and the data were processed using MassLynx 4.1 (Waters, Manchester, UK). The crystallographic structure of Rtt106_65-320_ (entry PDB code 3GYP [https://www.rcsb.org/structure/3GYP]), was visualized using PyMOL 2.2.0 software. Theoretical CCS was calculated using Mobcal^70^ from the crystal structure.

### Partial proteolysis followed by nondenaturing mass spectrometry analysis

Proteolysis of the Bcd1p_FL_/His_6_Rtt106p-M complex was carried out by mixing 0.7 U of sequencing grade modified porcine trypsin (Promega, Madison, USA) with 160 µg of complex incubated for 4 h at 20 °C. The sample was then buffer exchanged in 200 mM ammonium acetate (NH_4_Ac), pH 8 buffer using Zeba spin desalting columns (Thermo Fisher Scientific, Waltham, MA USA). The concentration of the complex was determined by UV absorbance on a Nanodrop 2000 spectrophotometer (Thermo Fisher Scientific USA). Nanoelectrospray mass spectrometry analysis was performed on an LC-time-of-flight mass spectrometer (Micromass) coupled with an automated chip-based nanoESI source (Triversa Nanomate, Advion Biosciences, Ithaca, NY). The sample was infused at 5 μM by dilution in 200 mM NH_4_Ac, pH 8 buffer. Instrumental parameters for analysis were optimized by raising the backing pressure to 7 mbar and the cone voltage to 200 V. Data were treated using MassLynx 4.1 software (Waters, Manchester, UK).

### HDX-MS

Samples were prepared and injected with an automated HDX system including a CTC PAL robot (Leap Technologies, Zwingen, Switzerland), coupled to a nanoAcquity UPLC system with HDX technology (Waters, Manchester, UK). Individual proteins and complex were prepared in a 10 mM potassium phosphate, 150 mM NaCl, pH 7.5 buffer. After dilution, the samples were incubated at 20 °C for different deuteration times (0.5, 2, 10, 30, and 60 min) in 10 mM potassium phosphate, 150 mM NaCl, pD7.5 deuterated buffer. The exchange reaction was stopped by adding 1:1 (v/v) of 100 mM potassium phosphate, 100 mM TCEP, 2 M GdHCl, pH 2.3 quench buffer at 1 °C for 0.5 min. Quenched samples were then digested (100 pmoles injections) through a pepsin-immobilized cartridge (Enzymate pepsin column, 300 Å, 5 µm, 2.1 × 30 mm, Waters, Manchester, UK) in 0.1% aqueous formic acid solution and generated peptides were trapped on UPLC precolumn (ACQUITY UPLC BEH C18 VanGuard precolumn, 2.1 mm I.D. × 5 mm, 1.7 µM particle diameter, Waters, Manchester, UK) at 200 µl.min^−1^. Digested peptides were then separated on UPLC column (ACQUITY UPLC BEH C18, 1.0 mm I.D. × 100 mm, 1.7 µM particle diameter, Waters, Manchester, UK) at 0.1 °C with a gradient elution of solvent A (0.1% formic acid aqueous) and solvent B (acetonitrile with 0.1% formic acid) [2–40% B (7 min), 40–85% B (0.5 min), and 85% B (1 min)] at a flow rate of 40 µL.min^−1^. Mass spectrometry analyses were conducted on a Synapt G2 HDMS (Waters, Manchester, UK) with an electrospray ionization in positive polarity, initially calibrated and using a lock-mass correction with glufibrinogen peptide. Analyses were carried out in data-independent acquisition mode (MS^E^, Waters, Manchester, UK) with the following parameters: ESI voltage, 3.2 kV; cone voltage, 40 V; source temperature, 80 °C; desolvation gas nitrogen at 150 °C and 800 L.h^−1^; acquisition range, *m/z* 50–2000; scan time, 0.3 s; trap MS collision, 15 → 40 eV. MS^E^ data were processed using ProteinLynx Global Server 2.5.3 (Waters, Manchester, UK) with a custom protein sequence library, where peptide and fragment tolerances were set automatically by PLGS, with oxidized methionine as variable modification. Data were then processed with DynamX 3.0 (Waters, Manchester, UK) as follows: all experiments were carried out in triplicate and only peptides identified in all replicates were kept with a minimum fragment of 0.2 per amino acid, a minimum intensity of 10^3^, a length between 5 and 30 residues and a file threshold of 3. Deuterium uptakes for all identified peptides were manually checked and validated. Only one charge state was kept for each peptide and deuterium uptakes were not corrected for back-exchange, represented as relative. HDX-MS results were statistically validated using a mixed effects model (MEMHDX^71^), with a *P* value set to 0.01. HDX data were exported on crystal structure using PyMOL 2.2.0 software.

### XL-MS experiments

#### XL reaction

The chemical XL agent used was an equimolar mix of light (d0) and heavy (d4) BS3. The XL experiment was performed in stock protein buffer (10 mM HEPES, 150 mM NaCl, pH 7.5). For XL reactions (20 µL), 70 µM of complex was used in two ratios (1:50 and 1:100) of BS3-d0/d4 and one control with no cross-link reagent. The reaction occurred for 1 h at room temperature and was then quenched by adding 1.05 µL of bicarbonate ammonium solution at 400 mM. Samples were then deposited and separated on acrylamide 10% SDS-PAGE. XL reaction were performed in technical duplicates on two independent Bcd1p_FL_/Rtt106p-M purification batches.

#### In-gel digestion

Bands corresponding to the cross-linked proteins for each XL reagent concentration were cut and subjected to in-gel digestion using an automated protein digestion system, MassPrep Station (Waters, Manchester, UK). The gel plugs were first destained three times with 100 µL of 25 mM ammonium hydrogen carbonate (NH_4_HCO_3_)/acetonitrile (ACN) (50/50). They were then dehydrated with 50 µL ACN. The cysteine residues were reduced by adding 50 µL dithiothreitol (DTT) at 10 mM for 30 min at 60 °C followed by 30 min at room temperature, and alkylated by adding iodoacetamide (IAA) at 55 mM for 20 min. The bands were then washed by adding 50 µL of NH_4_HCO_3_ 25 mM and 50 µL ACN. After dehydration with ACN, the gel bands were stored at −20 °C until digestion. The proteins were cleaved by adding 25 µL of a modified porcine trypsin solution at 4 ng/µL in 25 mM NH_4_HCO_3_. Digestion was performed overnight at 37 °C. Tryptic peptides were extracted twice: the first time with 40 µL of 60% ACN in 0.1% Formic Acid (FA) for 1 h and the second time with 40 µL of 100% ACN for 1 h.

The collected extracts were pooled and excess ACN was evaporated under vacuum with a SpeedVac™ (Thermo Scientific, Waltham, USA). All the extracted peptides were pooled in one well per XL reagent concentration and again evaporated under vacuum to a final volume of 57 µL.

#### XL-MS analysis

Nanocapillary liquid chromatography-tandem mass spectrometry (nanoLC-MS/MS) was performed with a nanoLC-Orbitrap-MS system. The system was controlled by X-Calibur™ (v.3.0.63, Thermo Scientific, Waltham, USA). The nanoLC system was composed of ACQUITY UPLC® BEH130 C18 column (250 mm×75 µm with 1.7 µm particle size, Waters Corporation, Milford, USA) and a Symmetry C18 precolumn (20 mm × 180 µm with 5 µm particle size, Waters Corporation, Milford, USA). The solvent system consisted of 0.1% FA in water (solvent A) and 0.1% FA in ACN (solvent B). Samples (2 µL) were loaded into the enrichment column for 5 min at 5 µL/min with 99% of solvent A and 1% of solvent B. Peptides were eluted at a flow rate of 450 nL/min with a 1–35% linear gradient of solvent B in 79 min. Analyses were performed on a Q-Exactive + (Thermo Scientific, Waltham, USA) with the capillary voltage set to 1.6 kV at 250 °C. The system was operated in data-dependent-acquisition (DDA) mode with automatic switching between MS (50 ms/scan on m/z range [300-1800] with R = 70,000) and MS/MS (100 ms/scan on m/z range [200-2200] with R = 17,500) modes. Samples digested in-gel were analyzed using the following MSMS method: the 10 most abundant peptides (intensity threshold 1.5.10^5^), excluding mono and doubled charged ions, were selected on each MS spectrum for further isolation and HCD fragmentation (dynamic exclusion 30 sec). Mass data collected during analysis were processed and converted into.mgf files using MSConvert. The MS/MS data were interpreted using StavroX software v.3.6.6^72^. Spectra were searched with a mass tolerance of 5 ppm for MS, 10 ppm for MS/MS and using trypsin as enzyme. Lys and Arg were considered as protease cleavage sites with a maximum of three missed cleavages. Carbamidomethylation of cysteine was set as fixed and oxidation of methionine as variable modifications (max. mod. 2). Primary amino groups (Lys side chains and N-termini) as well as primary hydroxyl groups (Ser, Thr, and Tyr side chains) were considered as cross-linking sites. Cross-links composed of consecutive amino-acid sequences were not considered. Heavy and light BS3 cross-linked peptides were validated using an FDR cutoff of 5%. Each conjugation site was manually validated based on the presence of y-ion and b-ion and peak intensity observed on the MS/MS spectra. After validation at the MS/MS level, QualBrowser (Thermo Xcalibur 3.0.63) was used to validate the equimolar ratio between the heavy and light cross-linked peptides at the MS level. Only peptides present in the two technical replicates of each individual cross-linker ratio were considered as validated. The list of validated cross-links for the complex Bcd1p_FL_:Rtt106p-M is in Supplementary Table 2. Finally, PyMOL 2.2.0 software. was used to calculate the Cα-Cα distance of each validated linkage sites.

### Reporting summary

Further information on research design is available in the Nature Research Reporting Summary linked to this article.

## Supplementary information

Supplementary Information

Reporting Summary

## Data Availability

Genetic interaction mapping data that support the findings of this study have been deposited in NCBI GEO with the accession code GSE118550. The gene ontology resource [http://geneontology.org/] was used for information on protein sequences. Bcd1 protein sequences used during the current study are available from UniProt with the entry identifiers in *Saccharomyces cerevisiae* ([https://www.uniprot.org/uniprot/P38772], *Homo sapiens* [https:// www.uniprot.org/uniprot/Q9NWK9], *Mus musculus* [https://www.uniprot.org/uniprot/Q3UFB2], *Schizosaccharomyces pombe* [https://www.uniprot.org/uniprot/O74906], and from GenBank with the accession numbers in *Talaromyces islandicus* [https://www.ncbi.nlm.nih.gov/protein/816194281], *Candida glabrata* [https://www.ncbi.nlm.nih.gov/protein/961789889], *Candida albicans* [https://www.ncbi.nlm.nih.gov/protein/723165737], *Pichia kudriavzevii* [https://www.ncbi.nlm.nih.gov/protein/1402408442], *Yarrowia lipolytica* [https://www.ncbi.nlm.nih.gov/protein/1078658162]. 3D data that support the findings of this study have been deposited in the Biological Magnetic Resonance Data Bank (access code 30570) and Protein Data Bank (entry code 6NZ2 [10.2210/pdb6NZ2/pdb]) for NMR data. X-ray data have been deposited in the Protein Data Bank (entry code 6THL [10.2210/pdb6THL/pdb). The mass spectrometry and proteomic data that support the findings of this study have been deposited to the ProteomeXchange Consortium via the PRIDE [https://www.ebi.ac.uk/pride/archive/] partner repository with the dataset identifier PXD023434. This submission includes all HDX-MS raw data files, Cluster and State data files (csv output files from DynamX), H/D plots ppt file, XL-MS raw data files, an Excel file summarizing MS information from all validated XL peptides, a FASTA file of the sequences used in structural MS experiments, Gel bands analyses raw data files, FASTA file used for Mascot research and excel file summarizing MS information of all peptides and proteins identified. Source data are provided with this paper.

## Source data

Source Data
