# Supplementary material for: The box C/D snoRNP assembly factor Bcd1 interacts with the histone chaperone Rtt106 and controls its transcription dependent activity
Source: Nat Commun. 2021 Mar 25;12:1859. doi: 10.1038/s41467-021-22077-4 (PMC7994586; doi:10.1038/s41467-021-22077-4)
Supplement: Supplementary file 1 — Supplementary Information [file 41467_2021_22077_MOESM1_ESM.pdf]

The box C/D snoRNP assembly factor Bcd1 interacts with the histone chaperone Rtt106 and controls its transcription dependent activity

Benoît Bragantini[1], Christophe Charron[1], Maxime Bourguet[2], Arnaud Paul[1], Decebal Tiotiu[1], Benjamin Rothé[1], Hélène Marty[1], Guillaume Terral[2], Steve Hessmann[2], Laurence Decourty[3], Marie-Eve Chagot[1], Jean-Marc Strub[2], Séverine Massenet[1], Edouard Bertrand[4], Marc Quinternet[5], Cosmin Saveanu[3], Sarah Cianférani[2], Stéphane Labialle[1*], Xavier Manival[1*], Bruno Charpentier[1*]

[1] Université de Lorraine, CNRS, IMoPA, Nancy F-54000, France

[2] Laboratoire de Spectrométrie de Masse BioOrganique, Université de Strasbourg, CNRS IPHC UMR 7178, F-67000 Strasbourg, France

[3] Génétique des Interactions Macromoléculaires, Département de Génomes et Génétique, Institut Pasteur (UMR3525-CNRS), F-75015 Paris, France

[4] IGH, CNRS, Université de Montpellier, F-34293 Montpellier, France

[5] Université de Lorraine, CNRS, INSERM, IBSLor, Nancy F-54000, France

These authors contributed equally: Benoît Bragantini, Christophe Charron, Maxime Bourguet, Arnaud Paul

These authors jointly supervised this work: Bruno Charpentier, Stéphane Labialle, Xavier Manival

* Correspondence
bruno.charpentier@univ-lorraine.fr, Tel : +33 3 72 74 66 27; Fax: +33 3 72 74 65 45
stephane.labialle@univ-lorraine.fr, Tel : +33 3 72 74 66 51
xavier.manival@univ-lorraine.fr, Tel : +33 3 72 74 66 91

Present Address:

Bragantini Benoît, Department of Biochemistry and Molecular Biology, Mayo Clinic, Rochester, Minnesota, 55905, USA

Rothé Benjamin, Ecole polytechnique fédérale de Lausanne (EPFL) SV ISREC, Station 19, CH-1015 Lausanne, Switzerland

Supplementary Introduction

The PAQosome was found in association with sub-complexes of RNA polymerase II[1] (RNAPII) and participates in its assembly in cytoplasm[2]. It is also involved in the stabilization and assembly of phosphatidylinositol 3-kinase-related kinases (PIKKS)[3-6], the U4 and U5 small nuclear RNPs[7-9], RNA polymerases I and III[2,10] and the TSC complex, a tumor suppressor and important regulator of mTOR[8,9].

Supplementary Figures

a

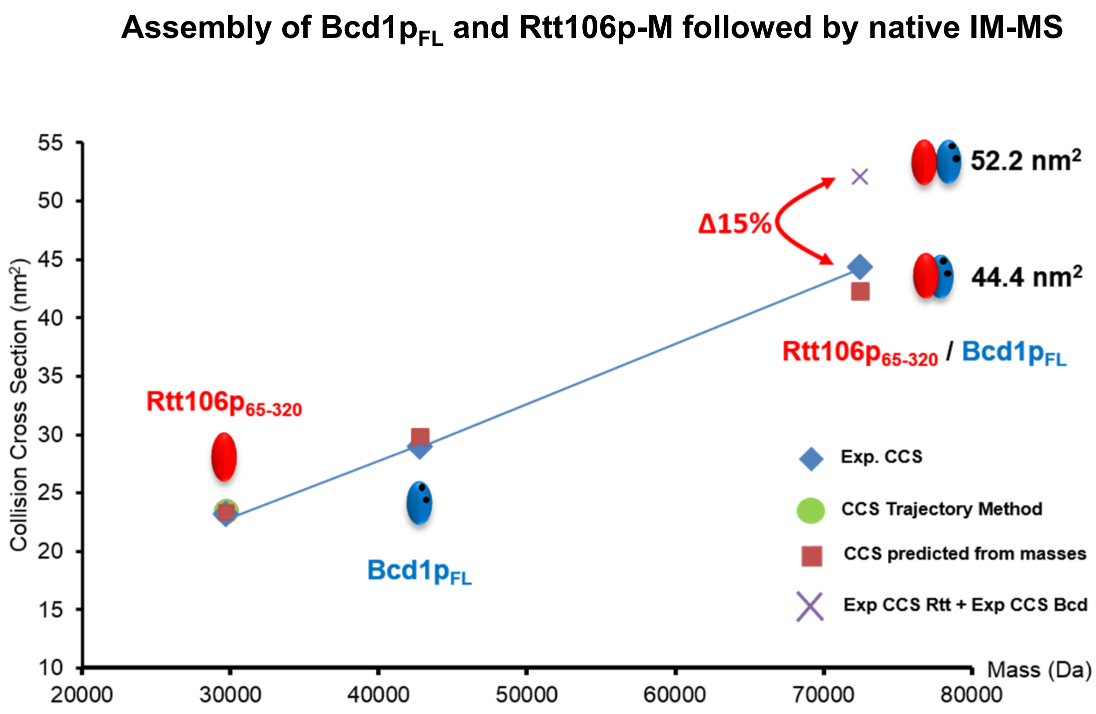

b

Protein	Experimental CCS (nm^2)	CCS predicted from masses (nm^2)	CCS Trajectory Method (nm^2)
**$Bcd1p_{FL}$**	**29.0 ± 1.5**	**29.8**	**-**
**$Rtt106p_{65\text{-}320}$**	**23.2 ± 1.9**	**23.4**	**23.5**
**$\sum CCS_{exp}$**	**52.2 ± 3.4**	**53.2**	**-**
**$Bcd1p_{FL}$/ $Rtt106p_{65\text{-}320}$**	**44.4 ± 1.4**	**42.3**	**-**

-15%

Fig. 1 Assembly of Bcd1p and Rtt106p-M followed by native IM-MS. a The native Ion Mobility-Mass Spectrometry (IM-MS) parameters were fine tuned in order to measure the arrival time distributions (ATDs) of each most abundant charge state for individual partners and complex in the same IM cycle and to avoid ion activation in the IM cell (see Methods section). Theoretical Collision Cross Sections (CSS) were calculated with Mobcal while Predicted CCS from masses were estimated from Ruotolo et al. (2008)[11] : $\Omega = 2.435 \times Mw^{2/3}$. **b** Measured CCSs obtained for each individual subunit and for the $Bcd1p_{FL}$:Rtt106p-M complex were then compared to CCS values deduced either from the mass or from 3D structures when available. A significant difference of -15 % in CCS was observed between the juxtaposed model and the measured IM-MS CCS, arguing strongly in favor of a close imbrication of $Bcd1p_{FL}$ and Rtt106p-M upon complex formation.

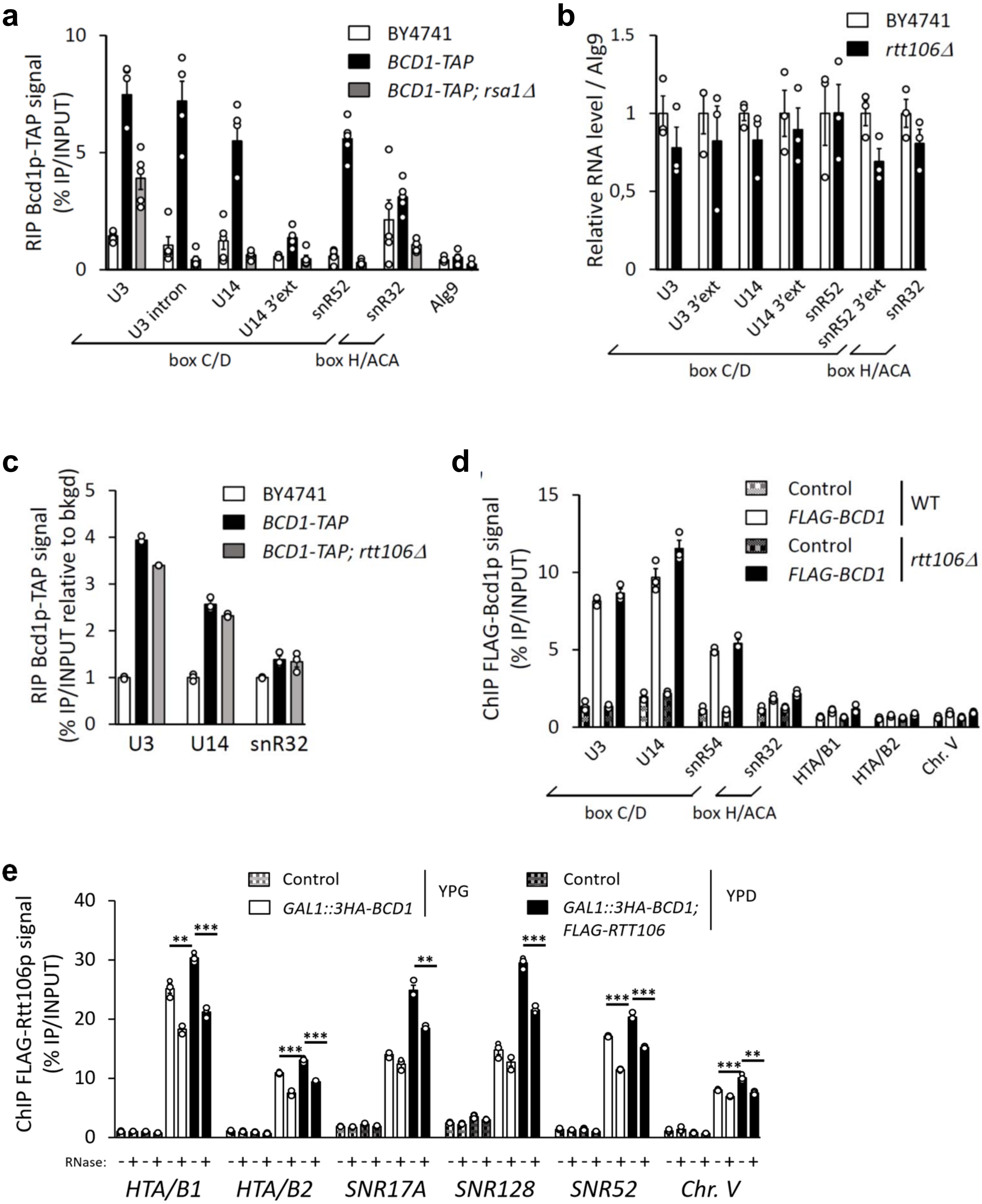

a
RIP Bcd1p-TAP signal (% IP/INPUT)
10
5
0
BY4741
BCD1-TAP
BCD1-TAP; rsa1Δ
U3
U3 intron
U14
U14 3'ext
snR52
snR32
Alg9
box C/D
box H/ACA
b
Relative RNA level / Alg9
1.5
1
0,5
0
BY4741
rtt106Δ
U3
U3 3'ext
U14
U14 3'ext
snR52
snR52 3'ext
snR32
box C/D
box H/ACA
c
RIP Bcd1p-TAP signal (% IP/INPUT relative to bkgd)
5
4
3
2
1
0
BY4741
BCD1-TAP
BCD1-TAP; rtt106Δ
U3
U14
snR32
d
ChIP FLAG-Bcd1p signal (% IP/INPUT)
15
10
5
0
Control
FLAG-BCD1
WT
Control
FLAG-BCD1
rtt106Δ
U3
U14
snR54
snR32
HTA/B1
HTA/B2
Chr. V
box C/D
box H/ACA
e
ChIP FLAG-Rtt106p signal (% IP/INPUT)
40
30
20
10
0
Control
GAL1::3HA-BCD1
YPG
Control
GAL1::3HA-BCD1; FLAG-RTT106
YPD
**
***
***
***
**
***
***
***
***
**
RNase: -+-+-+-+
HTA/B1
HTA/B2
SNR17A
SNR128
SNR52
Chr. V

f

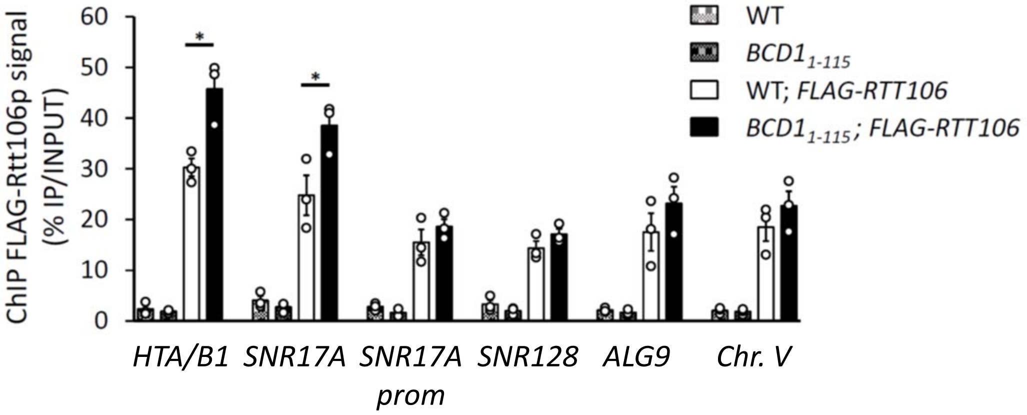

Fig. 2 Rtt106p does not contribute to association of Bcd1p to snoRNAs loci nor to the steady state level of snoRNAs. a and c Association of Bcd1p with RNAs. RNA immunoprecipitation (RIP) assays were performed on extracts prepared from WT *BCD1-TAP* cells and cells disrupted of the *RSA1* (*rsa1Δ*) or *RTT106* (*rtt106Δ*) ORFs. The cells were cultivated in the presence of galactose (YPG) or shifted to a glucose containing medium (YPD) for 6 h before preparation of the extract. IP was performed using IgG-sepharose beads. Data are mean values plus standard error of the mean of four to five (panel A) and three (panel C) biological replicates. **b** Quantification of snoRNAs. RT-qPCR analyses were performed on total RNAs extracted from WT BY4741 strain and mutant KO for the *RTT106* ORF (*rtt106Δ*). The *ALG9* gene was used as an endogenous control. Relative RNA expression of WT strain was set to one. Data are mean values plus standard error of the mean of three biological replicates. **d** Chromatin immunoprecipitation (ChIP) assays were performed on yeast *FLAG-BCD1* cells with (*rtt106Δ*) or without the disruption of the *RTT106* ORF. Cells were cultivated in YPG or shifted to YPD medium before ChIP assays. Cells expressing non-tagged Bcd1p were used as controls and IP was performed using anti-FLAG antibody. Data are mean values plus standard error of the mean of three biological replicates. **e** ChIP assays performed on yeast *GAL1::3HA-BCD1* transformed by empty or *FLAG-RTT106* expression vector. The BY4741 strain was used as control. Cells were cultivated in YPG or shifted to YPD medium before ChIP assays. Before incubation with anti-FLAG agarose beads, the cell lysate was incubated (+) or not (-) with RNases A/T1 (Paul et al., 2019). Data are mean values plus standard error of the mean of three biological replicates. Two-tailed t-tests (**P* < 0.05, ***P* < 0.01, ****P* < 0.001). RNase + versus RNase - in YPG: ** = 0.002 for *HTA/B1*, *** = 0.0003 for *HTA/B2*, *** = 2.94 E-6 for *SNR52*, *** = 0.0005 for *Chr. V*. RNase + versus RNase - in YPD: *** = 0.0002 for *HTA/B1*, *** = 0.0002 for *HTA/B2*, ** = 0.002 for *SNR17A* (U3), *** = 0.0003 for *SNR128* (U14), *** = 0.0002 for *SNR52*, ** = 0.003 for *Chr. V*. **f** ChIP assays performed on WT or $BCD1_{1\text{-}115}$ yeast strains. Cells were cultivated in YPG or shifted to YPD medium before ChIP assays. Cells expressing non-tagged Rtt106p were used as controls and IP was performed using anti-FLAG antibody. Data are mean values plus standard error of the mean of three biological replicates.

Two-tailed t-tests (**P* < 0.05, ***P*< 0.01, ****P* < 0.001). WT; *FLAG-RTT106* versus $BCD1_{1\text{-}115}$; *FLAG-RTT106*: * = 0.018 for *HTA/B1*, * = 0.047 for *SNR17A* (U3).

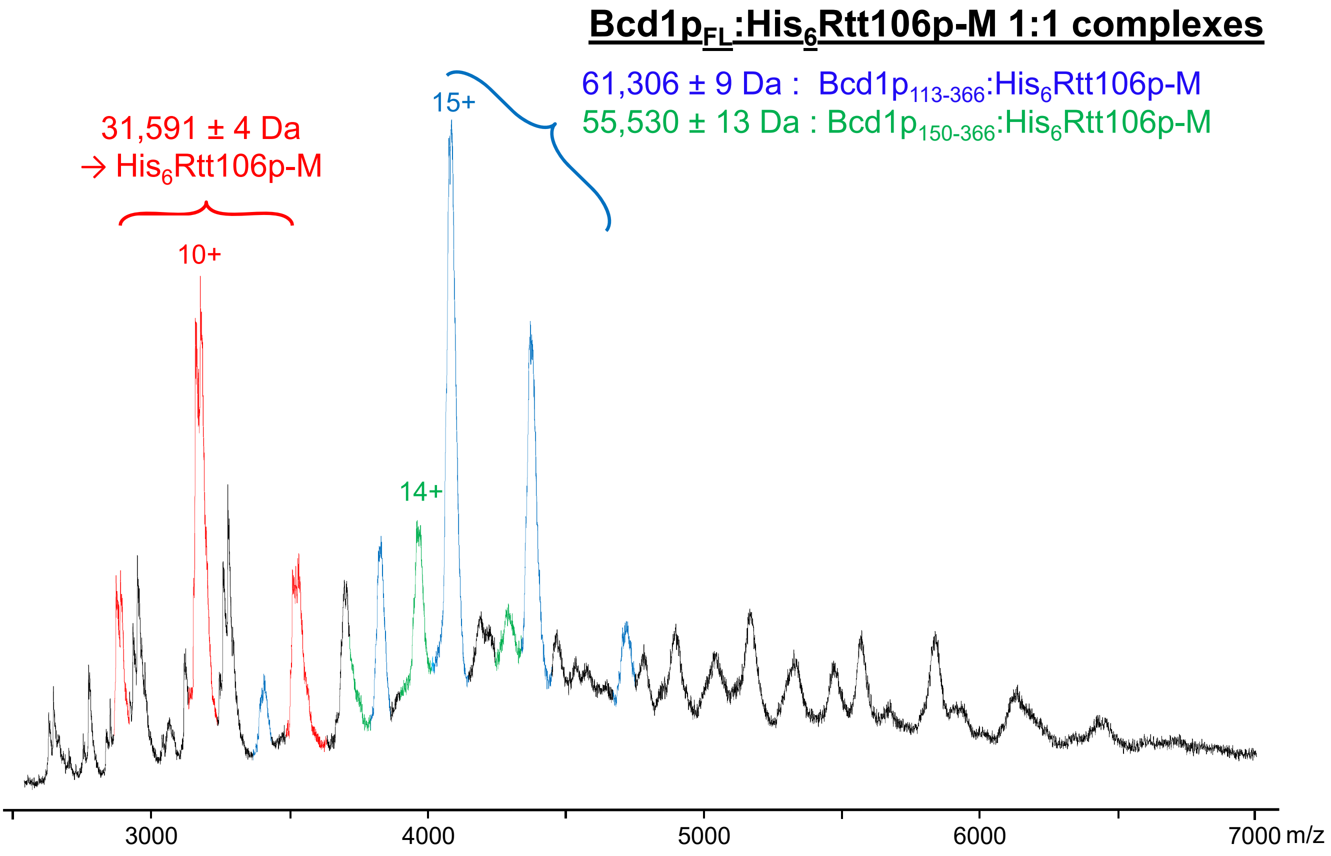

Fig. 3 Limited proteolysis followed by non-denaturing mass spectrometry analysis of Bcd1p$_{FL}$:His$_6$Rtt106p-M. After 4 h of tryptic digestion, we observed that His$_6$Rtt106p–M was almost completely resistant to enzymatic digestion, only one C-terminal amino acid was removed (red trace). In the other part, two other species corresponding to two 1:1 Bcd1p:His$_6$Rtt106p–M complexes were detected: the major species corresponds to truncated Bcd1p$_{113-366}$ in interaction with His$_6$Rtt106p–M (blue trace), while a minor species corresponding to a smaller fragment of Bcd1p$_{150-366}$ still in interaction with His$_6$Rtt106p–M (green trace) was also detected (all identified fragments were validated by LC-MS/MS, data not shown). Molecular weight is shown in Dalton.

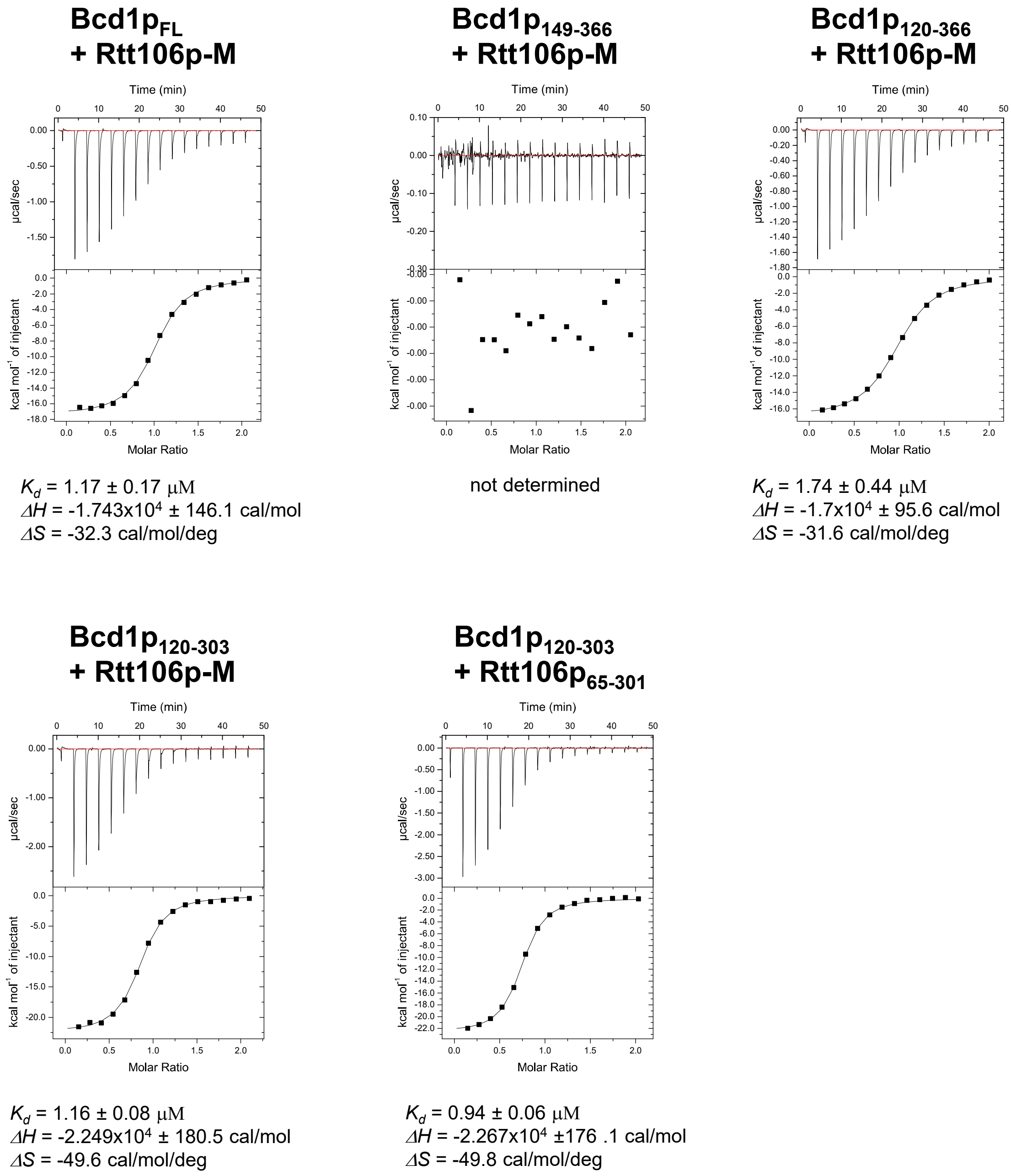

Fig. 4 Interaction analysis between Bcd1p and Rtt106p fragments by isothermal titration calorimetry (ITC). ITC results between (from left to right) Bcd1p$_{FL}$ with Rtt106p-M, Bcd1p$_{149-366}$ with Rtt106p-M, Bcd1p$_{120-366}$ with Rtt106p-M, Bcd1p$_{120-303}$ with Rtt106p-M, and Bcd1p$_{120-303}$ with Rtt106p$_{65-301}$ recorded at 293 K in buffer containing 10 mM NaPi at pH 7.5, 150 mM NaCl and 0.5 mM TCEP. The calculated dissociation constant (*Kd*), and the variations in enthalpy (*ΔH*) and entropy (*ΔS*) are indicated.

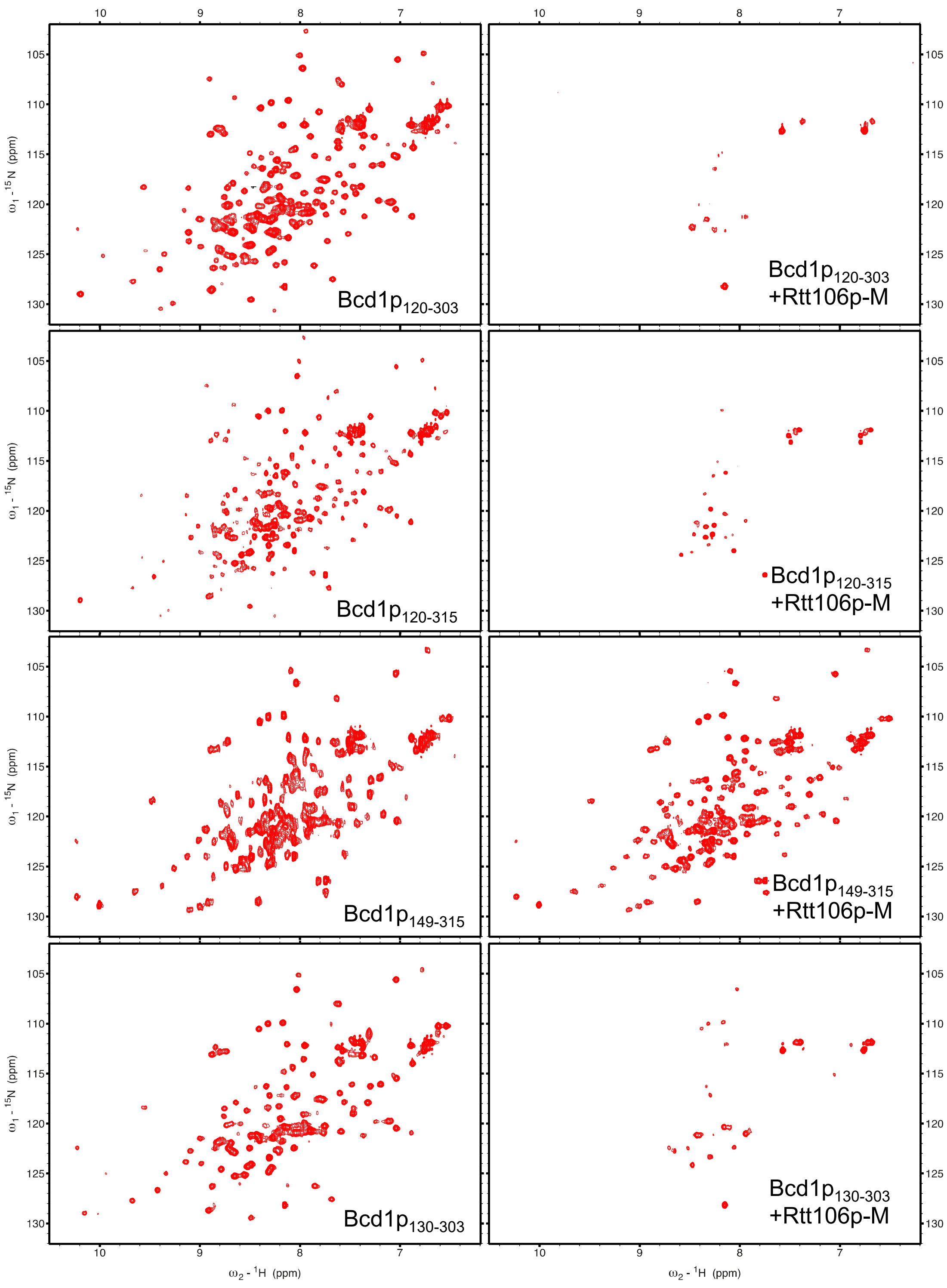

Fig. 5 Interaction analysis between Bcd1p and Rtt106p fragments by NMR titrations. NMR ^{1}H-^{15}N-HSQC of Bcd1p fragments alone (on left) or with Rtt106p-M (on the right). Bcd1p fragments tested are (from the top to the bottom) Bcd1p$_{120\text{-}303}$, Bcd1p$_{120\text{-}315}$, Bcd1p$_{149\text{-}315}$ and Bcd1p$_{130\text{-}303}$. Spectra were recorded at 293 K with protein concentration of 200 μM in 10 mM NaPi buffer (pH 6.4), 150 mM NaCl and 1 mM DTT.

a

```
BCD1_SACCE      ------------------------------------------------------------ 0
BCD1_HUMAN      MEFAAENEGKSGGGLHSVAEGVRLSPEPGREGVRDLAGAEEFGGGEEGTGLTGIKEIGDG 60
BCD1_MOUSE      MESAAEKEGTPGGGSQRVAEGARPRPAAGGEGARDLDGSPEAGDGEERNGLAGTKTTE-- 58

BCD1_SACCE      ------------------------------------------------------------ 0
BCD1_HUMAN      EEGSGQRPEEIPMDLTVVKQEIIDWPGTEG-RLAGQWVEQEVEDRPEVKDENAGVLEVKQ 119
BCD1_MOUSE      ------DAEEIKMDLAVVKQEVVDWSDLDSGVADSQWVKQEVEGGPEVKDE-KGVLEVKQ 111

BCD1_SACCE      ------------------------------------------------------------ 0
BCD1_HUMAN      ETDSSLVVKEAKVGEPEVKEEK--VKEEVMDWSEVKEEKDNLEIKQEEKFVGQCIKEELM 177
BCD1_MOUSE      EADSSLVVKEEEVDEPEVKEEKVKVKEEVTDWEEVKEEDLTI---KQELFVGQNVKEEQV 168

BCD1_SACCE      --------------------------------------MAVLCGVCGIKEFKYKCPRCLV 22
BCD1_HUMAN      HGECVKEEKDFLKKEIVDDTKVKEEPPINHPVGCKRKLAMSRCETCGTEEAKYRCPRCMR 237
BCD1_MOUSE      MDAAPIKEEGSLKSEAMEDAKVKEEPQMNPRVGSKRKLALSRCETCGTEEAKYRCPRCMR 228
                                                          * .** :* **:****:

BCD1_SACCE      QTCSLECSKKHKTRDNCSGQTHDPKEYISSEALKQADDDKHERNAYVQRDYNYLTQLKRM 82
BCD1_HUMAN      YSCSLPCVKKHKAELTCNGVRDK-TAYISIQQFTE---------MNLLSDYRFLEDVART 287
BCD1_MOUSE      FSCSLPCVKKHKADLTCSGVRDK-TAYVSLQQFTE---------MNLLSDYRFLEDVART 278
                 :*** * ****:  .*.*  .. . *:* : :.:           :  **.:* :: *

BCD1_SACCE      VHVQKMDARMKNKRVLGPVGGHNSNFKKRRYDIDEDDRDSTECQRIIRRGVNCLMLPKGM 142
BCD1_HUMAN      ADHISRDAFLK-----RPISNKYMYF---------------MKNRARRQGINLKLLPNGF 327
BCD1_MOUSE      ADKVSRDTFLK-----RPKRKKYLFF---------------MKNRARKQGIYLRLLPNGF 318
                ..  . *: :*       *   :   *                 :*  ::*:   :**:*:

BCD1_SACCE      QRSSQNRSKWDKTMDLFVWSVEWILCPMQEKGEKKELFKHVSHRIKETDFLVQGMGKNVF 202
BCD1_HUMAN      TKRKENSTFFDKKKQQFCWHVKLQFPQSQAEYIEKRVPDDKTINEILKPYIDPEKSDPVI 387
BCD1_MOUSE      SKRKENSTVFDHRKQQFCWHVKLQFPQSQAEYIEKRVPDDKTINEILKPYIDPEESDPVI 378
                 : .:* : :*:  : * * *:  :   * :  :*.: .. : .   . ::    .. *:

BCD1_SACCE      QKCCEFYRLAGTSSCIEGEDGSETKEERTQILQKSGLKFYTKTFPYNTTHIMDSKKLVEL 262
BCD1_HUMAN      RQRLKAYI--------RSQTGVQIL-MKIEYMQQNLVR-YYELDP--------------- 422
BCD1_MOUSE      RQRLKAYA--------QSQTGVQIL-MRVENMQQNMIR-YHELDP--------------- 413
                ::  : *         ..: * :    : : :*:. :: * : *

BCD1_SACCE      AIHEKCIGELLKNTTVIEFPTIFVAMTEADLPEGYEVLHQEPRPLEHTSTLNKFIDNARE 322
BCD1_HUMAN      ---YKSLLDNLRNKVIIEYPTLHVVLKGSNN--DMKVLHQVKSEST------KNVGNEN- 470
BCD1_MOUSE      ---YKSLSDNLKDKVIIEYPTLHVVLRGSSN--DKQLL-QVKSESA------QKLGNGN- 460
                    *.: : *::..:**:**:.*.:  :.   . ::* *            : :.* .

BCD1_SACCE      EEDAEEDSQPTEEPVQKETQDASDSDSDSDDDYNPGLSMDFLTA 366
BCD1_HUMAN      -------------------------------------------- 470
BCD1_MOUSE      -------------------------------------------- 460
```

b

```
BCD1_SCHPO    ----------------------------------MSENRLGICSTCQKNASKYRCPRCDS 26
BCD1_TALIS    ---------------------------------MSEPLLSELCTICHTTPPKYTCPRCAI 27
BCD1_SACCE    --------------------------------------MAVLCGVCGIKEFKYKCPRCLV 22
BCD1_CANGL    ---------------------------------------MGLCEVCNVEEFKYKCPRCFK 21
BCD1_CANAL    ---------------------------------MDFGEENTVCTICHENKSKYTCPACEI 27
BCD1_CANKR    ----------------------------------------MHCEICKKIEHKYVCPRCKL 20
BCD1_YARLI    MH--------ELKRFTI-QKKEKNHTHTHTHTHTQTTTHSMSCSICQQ-ESKYRCPACSA 50
                                                        *  *     ** ** *

BCD1_SCHPO    RFCCLECNLEHKRLTKCSGERDPAT-----FVPKSKLV----------NHLNSDFNFLSG 71
BCD1_TALIS    RTCSLACARRHKNWSQCSGVRDPAA-----YATRAELQ--------TASALDRDFNFITG 74
BCD1_SACCE    QTCSLECSKKHKTRDNCSGQTHDPK----EYISSEALKQADDDKHERNAYVQRDYNYLTQ 78
BCD1_CANGL    KTCSLACSKQHKADESCSGKSHDPT----AYIPRTDIKEADDENHESNILVQRDYNYLIN 77
BCD1_CANAL    KTCSLQCYNKHKYERDCTGKVDSNK-----YLNRSELAS-------DPVHLNRDYNFLNN 75
BCD1_CANKR    RTCSLPCFKQHKIDKKCSGLSDISLGTRDTYIDKK-QL--------DSNDVQRDYNFLLK 71
BCD1_YARLI    RTCSLACSKQHKASEKCSGLPDPTK-----YLNREALF--------TDTSVNRDYRFLKR 97
                *.* *  .**    *.*  .        :                   :  *:.::

BCD1_SCHPO    VERLINRKENGSHQVS-------------NRAERNRLQ--------------LKR----- 99
BCD1_TALIS    VERGLERAEREARARGFVDLDHEYMQDPKNKRKRKRRDGSKEEAAGGGGAGGSKKLVKGE 134
BCD1_SACCE    LKRMVHVQKMDARMKNKRVLGPVGGHNSNFKKRRYDIDEDD-----RDST--ECQ----- 126
BCD1_CANGL    MRREVEVQIDDSKRKNKRILREIYDPYMANKR-------QR-----GNSD--NSS----- 118
BCD1_CANAL    VDRKIHVGKEDIVKSAKNVFKRTRNQSRAPGNKRHK--RND-----EDNT--DKRIMAVK 126
BCD1_CANKR    VNRSLDLGKRRKS--EMKILKTVGNRRN--GNFS------------NNGI--NSK----- 108
BCD1_YARLI    LERDIVVRKQDGE--SMPIFKRARYNQN-----R------------KDGK--DGS----- 131
              : *

BCD1_SCHPO    ---------SLERAGINIKFAPPSNKKRRLNRTHYDKKSHLIKWSIEWCLHESSTSK--- 147
BCD1_TALIS    LAFLR----AAQDAGVKVERSPRGMTRNKENKSRFHPKHKCLSWTVDWIHTAGRSRV--- 187
BCD1_SACCE    ---------RIIRRGVNCLMLPKGMQRSSQNRSKWDKTMDLFVWSVEWILCPMQEKGEKK 177
BCD1_CANGL    ---------RLIRRGVSCLMLPKGMQRSLQNKSKWDNSLNQFTWTIEWVLCGDG------ 163
BCD1_CANAL    KVFLNEPTISIKRENTLIVSLPIGMSRSNTNKTGFDKKLNSFVWTIEWIVLNDQGEQ--- 183
BCD1_CANKR    ---------WVPTRGVKVKKVPLGMERGKLNKSG--GKGNNWAWTIEWLLIDENKKV--- 154
BCD1_YARLI    ---------VMERNGVKIHKVAQGMGRQKRNHSRWDPNIKQFCWTVEWVNVDTNETM--- 179
                            .        .  :   * :      .   * :.

BCD1_SCHPO    ----DLTDEASENTIITHSHPESEPLEKIFRKLVEENSEMNSQ----------------- 186
BCD1_TALIS    -VQNYLETI-----TI----G-----Q-AYDRAYPPLPDEATS----------------- 214
BCD1_SACCE    ELFKHVSHRIKETDFLVQGMG-----KNVFQKCCEFYRLAGTSSCIEGEDGS-------- 224
BCD1_CANGL    DTVTHLTHRAKENESVVEGIS-----KIVFNKIQTFYKIENGDGD--ETQAV-------- 208
BCD1_CANAL    -MTKFISYRLKESLILQD--------------SVPMNIINNKLNQ--------------- 213
BCD1_CANKR    -IDRYVKYKSGESSILRT--------------IVPRAWICGGDEY--------------- 184
BCD1_YARLI    -TVDKVNPV---QSLLEC--------------FQKRGSEKGGNGDNKGERKVEKNDTEQT 221
                   :         :

BCD1_SCHPO    ------------VS---KA-NIHAPDDMQFMIKSRKSRSK-----GFIYKKIEPSNSLSS 225
BCD1_TALIS    -----------TPKEENKTEPFPPHRNIYIYIRRQ-------ADSHIVLAPLHPEAKLAD 256
BCD1_SACCE    ------------ETKEERTQIL-QKSGLKFYTKTFPYNTTHIMDSKKLVELAIHEKCIGE 271
BCD1_CANGL    ------------LSREDRIALI-KDYNLEFYIKWFPYNTTEMSDSRNLIRIDAVNSTLGD 255
BCD1_CANAL    --------------------PV-EKSHLKFYLHDVIHKNKMI-------R-LNSEEKISD 244
BCD1_CANKR    ----------------------------TILFKDLE--SG------VFEV-VDKDAKLSE 207
BCD1_YARLI    EMKDEHSKQKDGDKKDEEKEVV-PLTPRSFYMKRIKSKNT------SPIL-LDPSKPLSE 273
                                           :  :                        : .

BCD1_SCHPO    CLRNSFVFEVPTIHVFTSTTQ-VHTE----------SS----YETSSSEQSDDSSSSSSC 270
BCD1_TALIS    VLRGRTVYEYPRLYVKSESPADIESGAVDETHVLEETWLR-NNPTGIEAQDDDESDWTSS 315
BCD1_SACCE    LLKNTTVIEFPTIFVAMTEAD-LPEGYE----VLHQEP---RPLEHTSTL----NKF--- 316
BCD1_CANGL    VFKNRTVIEFPTIYITKSVQD-LPKGFK----VMIEEK---GSAPGSEEN----SRV--- 300
BCD1_CANAL    VLKDKIVLEYPTIYVTANDEC-LQDRIIDEFQLADEED---DDATGSSTD----ESSSSD 296
BCD1_CANKR    VLLNKLVIEFPTMYIFKGDSS-IITREK--V----LDD---DLSSG-------------- 243
BCD1_YARLI    NLKDKSVVEYPTIYYTNEEIV-ASDSDSDSD----SDS---DNDSG-------------- 311
               : .  : * * :.

BCD1_SCHPO    --ISDS----------EESSSDDELNELSNEKKANSPTQNVDTSS---KLSSVKF-QNED 314
BCD1_TALIS    EGSSEE--SEDDDESETGSDSDSESESQVS-DVKE-------------ETA--------- 350
BCD1_SACCE    -----I---DN-AREEEDAEEDSQPTEEPV-Q----------------KETQDAS-DSDS 349
BCD1_CANGL    -----NVAGSGSSSDTETDDSDAEPEEESS-KQDNTQNKNVVVENVSEATAIVDK-DTSD 353
BCD1_CANAL    DGDSDSDTSSVGDSSDEDNDSDSAPEETSS-KLPPFSQTFFETRS--DSKPIIEEIGSSE 353
BCD1_CANKR    --SSDSDAS-SDSSSDSSSDSDTPPEESSS-KPLET------------------------ 275
BCD1_YARLI    --SDDSDDS-DDSDDDSDDDSDSAPEEESA-RQPEMPDIVKTL-A--DS-AVNDMAGLED 363

BCD1_SCHPO    DEDRRKNSDGSEASYSLPPLFGSYFQKQGQY     345
BCD1_TALIS    -------------------------------     350
BCD1_SACCE    DSD-DDYNPGLSMDF----LTA---------     366
BCD1_CANGL    EED-DDYNPGVSLDF----LMS---------     370
BCD1_CANAL    VVE-EP-------------------------     358
BCD1_CANKR    -------------------------------     275
BCD1_YARLI    AFE-KAQEEGKQ-------------------     374
```

Fig. 6 Highlights of the conserved fragment 120-303 of Bcd1p (in bold). a Sequence alignment of Bcd1p$_{120\text{-}303}$ from *Saccharomyces cerevisiae* (top), ZNHIT6 (also called BCD1) from *Homo sapiens* and from *Mus musculus*. Amino-acid alignments built with Clustal Omega using sequences of yeast (Bcd1p, UniProt entry P38772 [https://www.uniprot.org/uniprot/P38772]), human (ZNHIT6, UniProt entry Q9NWK9 [https://www.uniprot.org/uniprot/Q9NWK9]), and mouse (ZNHIT6, UniProt entry Q3UFB2 [https://www.uniprot.org/uniprot/Q3UFB2]) BCD1 proteins[12]. The consensus symbols stand for "*" for strictly conserved groups, ":" for groups of very similar properties and "." for groups of weakly similar properties. The amino acids involved in hydrophobic, electrostatic interactions and hydrogen bonding are shown in bold and dark blue, red, and orange, respectively. **b** Sequence alignment of Bcd1p$_{FL}$ from different yeast species. SCHPO, *Schizosaccharomyces pombe* (top, UniProt:O74906 [https://www.uniprot.org/uniprot/O74906]); TALIS, *Talaromyces islandicus* (GenBank:CRG83776.1 [https://www.ncbi.nlm.nih.gov/protein/816194281]); SACCE, *Saccharomyces cerevisiae* (UniProt:P38772 [https://www.uniprot.org/uniprot/P38772]); CANGL, *Candida glabrata* (GenBank:KTB14807.1 [https://www.ncbi.nlm.nih.gov/protein/961789889]); CANAL, *Candida albicans* (GenBank:KHC39748.1 [https://www.ncbi.nlm.nih.gov/protein/723165737]); CANKR, *Pichia kudriavzevii* (GenBank:AWU76967.1 [https://www.ncbi.nlm.nih.gov/protein/1402408442]); YARLI, *Yarrowia lipolytica* (GenBank:AOW05274.1 [https://www.ncbi.nlm.nih.gov/protein/1078658162]). The RBD sequence of *S. cerevisiae* is in bold. The color code used for the residues is the same as in **a**.

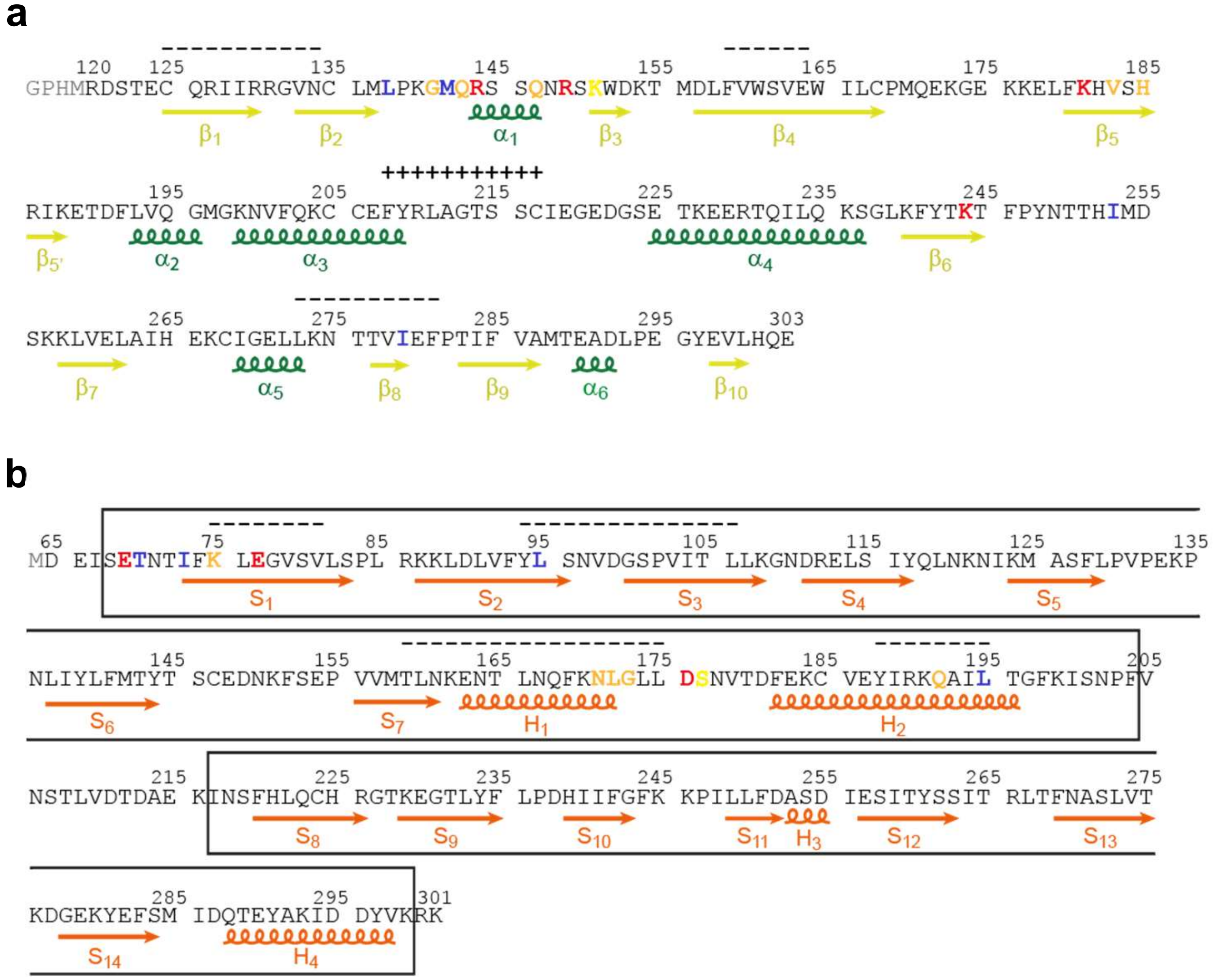

Fig. 7 Sequences of Bcd1p$_{120\text{-}303}$ and Rtt106p$_{65\text{-}301}$ with the summary of NMR, X-ray, cross-linking MS and HDX-MS results. a Sequence of Bcd1p$_{120\text{-}303}$. Secondary structure elements from NMR free structure are indicated below. The β_{10} strand is only present in the crystallographic structure. The first four residues (GPHM, in gray and not numbered) correspond to residues of the remaining 6xHistidines tag after cleavage by PreScission protease. The sequence numbering starts at residue R_{120} corresponding to the wild type. The cross–linked amino acid with Rtt106p is shown in bold and yellow, and the amino acids involved in hydrophobic and electrostatic interactions, and hydrogen bonding are shown in bold and dark blue, red, and orange, respectively. (-) less exposed residue to solvent after fixation of Rtt106p and (+) residue more exposed to solvent after fixation of Rtt106p (HDX-MS data from Supplementary figure 10). **b** Sequence of protein Rtt106p$_{65\text{-}301}$ with secondary structure elements from X-ray complex structure below (S=Strand; H=Helix). Amino acids interacting with Bcd1p are indicated with the same legend as in (A). The first frame represents the PH1 domain (68-204) and the second the PH2 domain (217-299). (-) less exposed residue to solvent after fixation of Bcd1p (HDX-MS data from Supplementary figure 10).

a

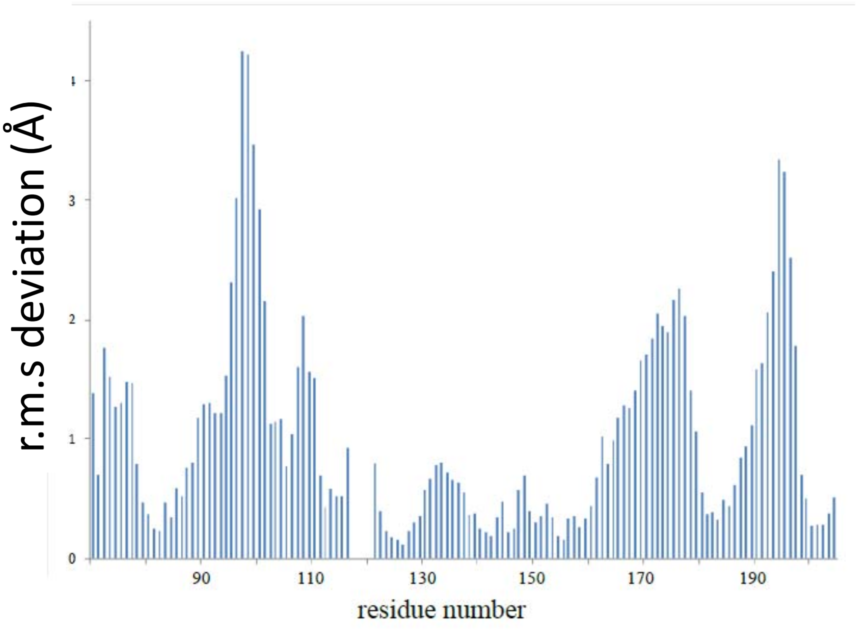

b

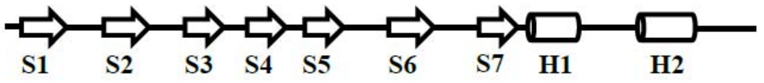

Fig. 8 Conformational changes of the PH1 domain of Rtt106p upon Bcd1p binding. a Values of R.M.S. deviations calculated by least squares minimized superimposition of PH1 domains. **b** Secondary structure elements of the PH1 domain of Rtt106p are assigned according to the Protein Data Bank (PDB) file entry. The seven β-strands are labeled S1 to S7 and the two α-helices are labeled H1 and H2. Note that structural modifications occur in β-strands S1 and S2, in the loops S2-S3 and S3-S4, and in the C-terminal region of the PH1 domain including α-helices H1 and H2 as well as the loop connecting these two α-helices.

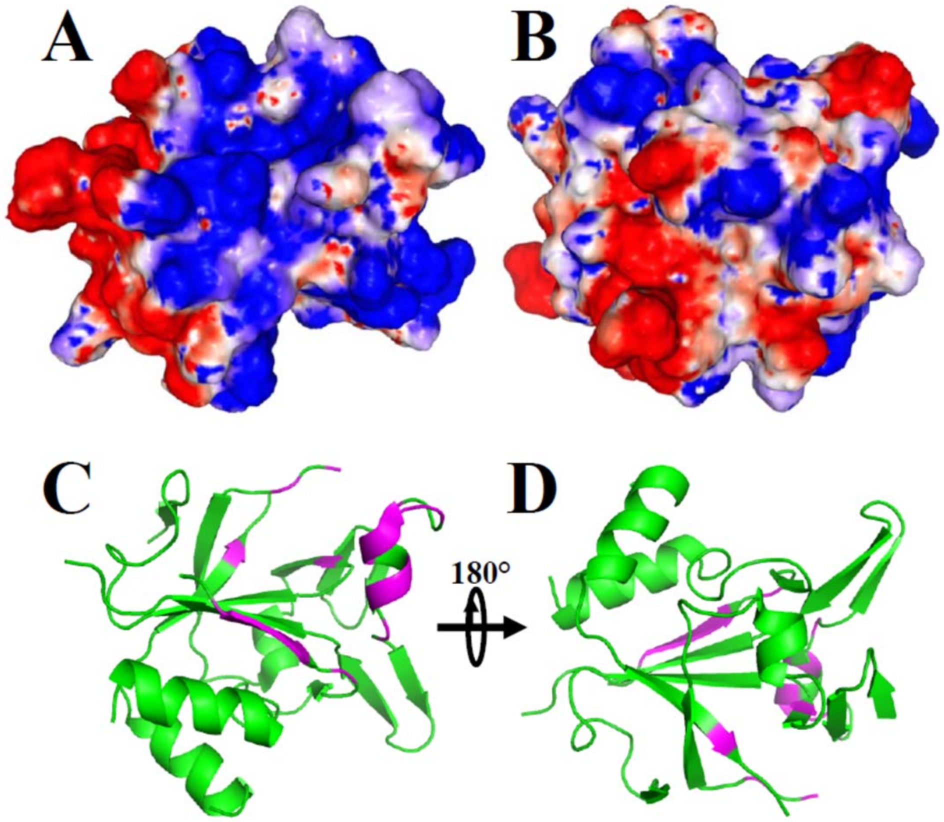

Fig. 9 Electrostatic properties of Bcd1p$_{120\text{-}303}$ (entry PDB code 6NZ2 [https://www.rcsb.org/structure/6NZ2]. **a-b** Electrostatic potentials mapped on the molecular surface of Bcd1p$_{120\text{-}303}$ and viewed in two opposite directions. Blue, white and red regions correspond to positive, neutral and negative electrostatic potentials, respectively (+1 kcal/(mole) in blue to -1 kcal/(mole) in red). **c-d** Opposite views (180 degrees apart) of ribbon representation at Bcd1p$_{120\text{-}303}$. Part of the structure that is buried upon binding of Rtt106p is colored magenta.

Panel **c** has the same orientation as panel **a** and panel **d** has the same orientation as panel **b**.

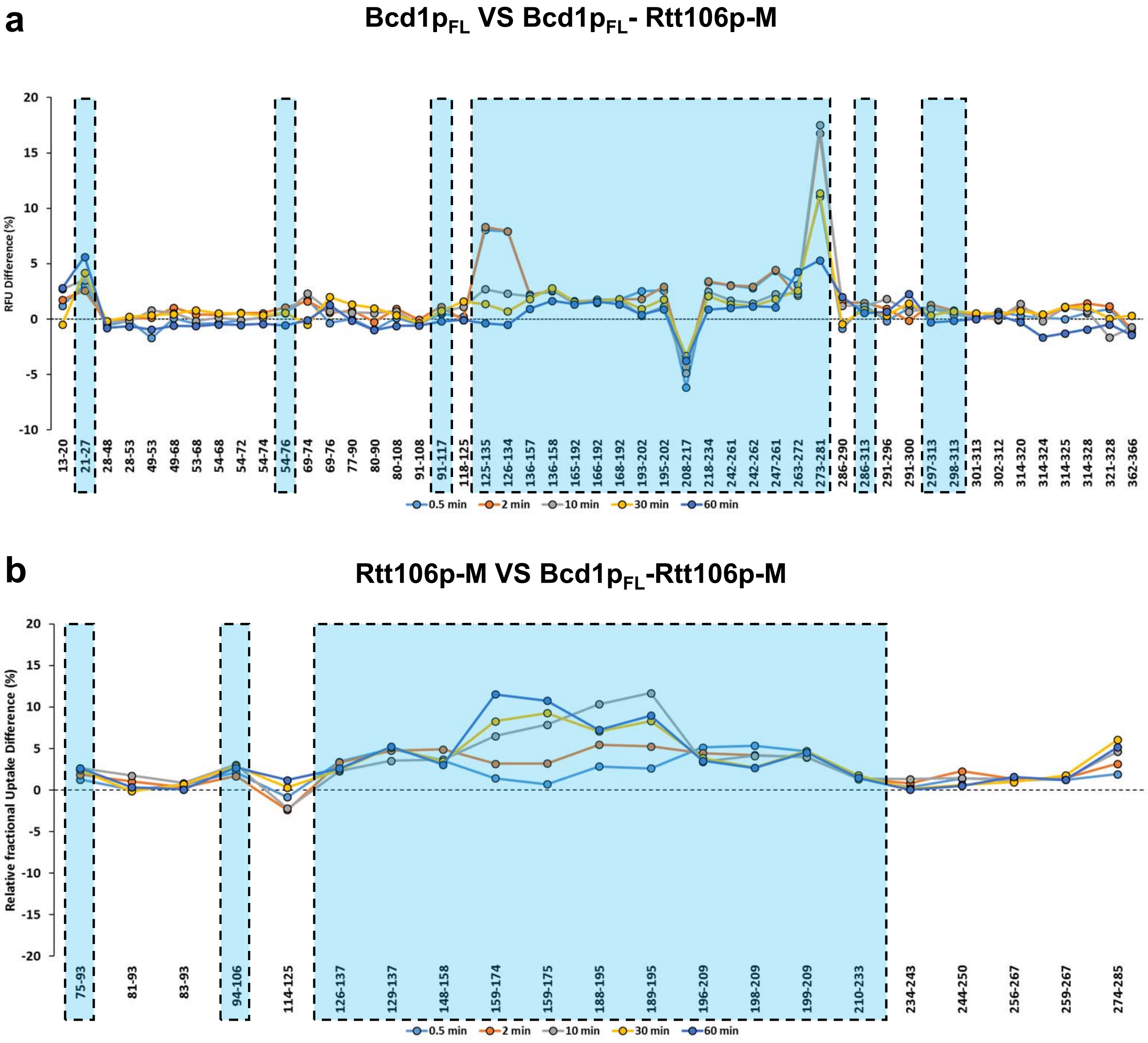

Fig. 10 Overview of structural mass spectrometry characterization of Bcd1p$_{FL}$:Rtt106p-M complex including HDX-MS. a Relative fractional uptake difference plot of Bcd1p$_{FL}$ vs Bcd1p$_{FL}$:Rtt106p-M HDX experiments. Each point represents the relative fractional uptake of each Bcd1p$_{FL}$ identified peptides from Bcd1p$_{FL}$:Rtt106p-M state subtracted from the relative fractional uptake of Bcd1p$_{FL}$ peptides from Bcd1p$_{FL}$ alone state (light blue circle shows the difference at 0.5 min exposure time, orange at 2 min of exposure, gray at 10 min of exposure, yellow at 30 min of exposure and dark blue at 60 min of exposure). Framed blue peptides represent the peptides whose magnitude of the difference was statistically significant in Wald tests, with a significance threshold set to 0.01 (MEMHDX software). **b** Relative fractional uptake plot of the difference in Rtt106p-M and Bcd1p$_{FL}$:Rtt106p-M HDX experiments. Each point represents the relative fractional uptake of each Rtt106p-M peptide identified from Bcd1p$_{FL}$:Rtt106p-M state subtracted from the relative fractional uptake of Rtt106p-M peptides from Rtt106p-M free state (the color code is the same as in **a**).

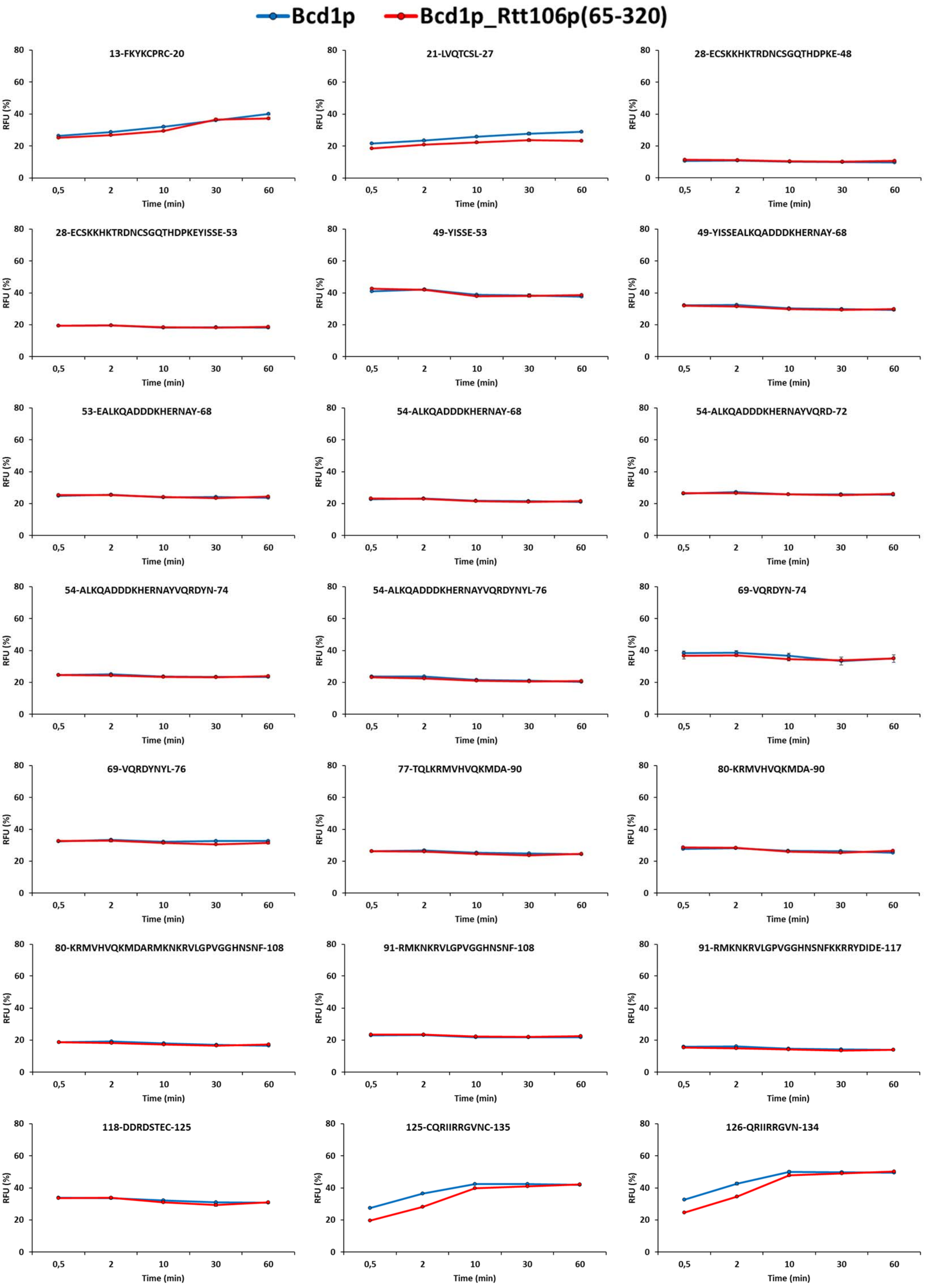
Bcd1p
Bcd1p_Rtt106p(65-320)
13-FKYKCPRC-20
21-LVQTCSL-27
28-ECSKKHKTRDNCSGQTHDPKE-48
28-ECSKKHKTRDNCSGQTHDPKEYISSE-53
49-YISSE-53
49-YISSEALKQADDDKHERNAY-68
53-EALKQADDDKHERNAY-68
54-ALKQADDDKHERNAY-68
54-ALKQADDDKHERNAYVQRD-72
54-ALKQADDDKHERNAYVQRDYN-74
54-ALKQADDDKHERNAYVQRDYNYL-76
69-VQRDYN-74
69-VQRDYNYL-76
77-TQLKRMVHVQKMDA-90
80-KRMVHVQKMDA-90
80-KRMVHVQKMDARMKNKRVLGPVGGHNSNF-108
91-RMKNKRVLGPVGGHNSNF-108
91-RMKNKRVLGPVGGHNSNFKKRRYDIDE-117
118-DDRDSTEC-125
125-CQRIIRRGVNC-135
126-QRIIRRGVN-134
RFU (%)
Time (min)
0
20
40
60
80
0,5
2
10
30
60

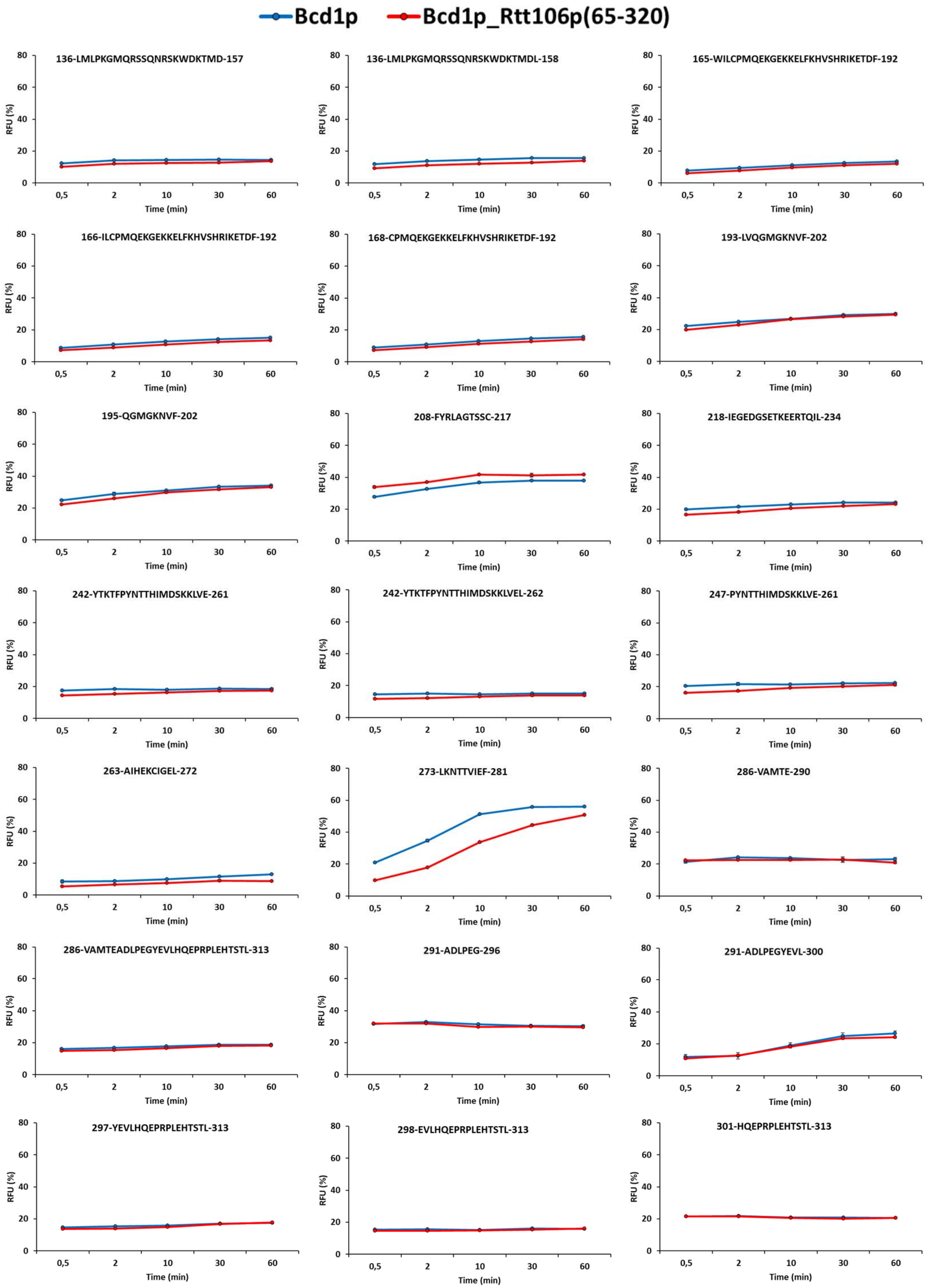
Bcd1p
Bcd1p_Rtt106p(65-320)
136-LMLPKGMQRSSQNRSKWDKTMD-157
136-LMLPKGMQRSSQNRSKWDKTMDL-158
165-WILCPMQEKGEKKELFKHVSHRIKETDF-192
166-ILCPMQEKGEKKELFKHVSHRIKETDF-192
168-CPMQEKGEKKELFKHVSHRIKETDF-192
193-LVQGMGKNVF-202
195-QGMGKNVF-202
208-FYRLAGTSSC-217
218-IEGEDGSETKEERTQIL-234
242-YTKTFPYNTTHIMDSKKLVE-261
242-YTKTFPYNTTHIMDSKKLVEL-262
247-PYNTTHIMDSKKLVE-261
263-AIHEKCIGEL-272
273-LKNTTVIEF-281
286-VAMTE-290
286-VAMTEADLPEGYEVLHQEPRPLEHTSTL-313
291-ADLPEG-296
291-ADLPEGYEVL-300
297-YEVLHQEPRPLEHTSTL-313
298-EVLHQEPRPLEHTSTL-313
301-HQEPRPLEHTSTL-313
RFU (%)
Time (min)
0
20
40
60
80
0,5
2
10
30
60

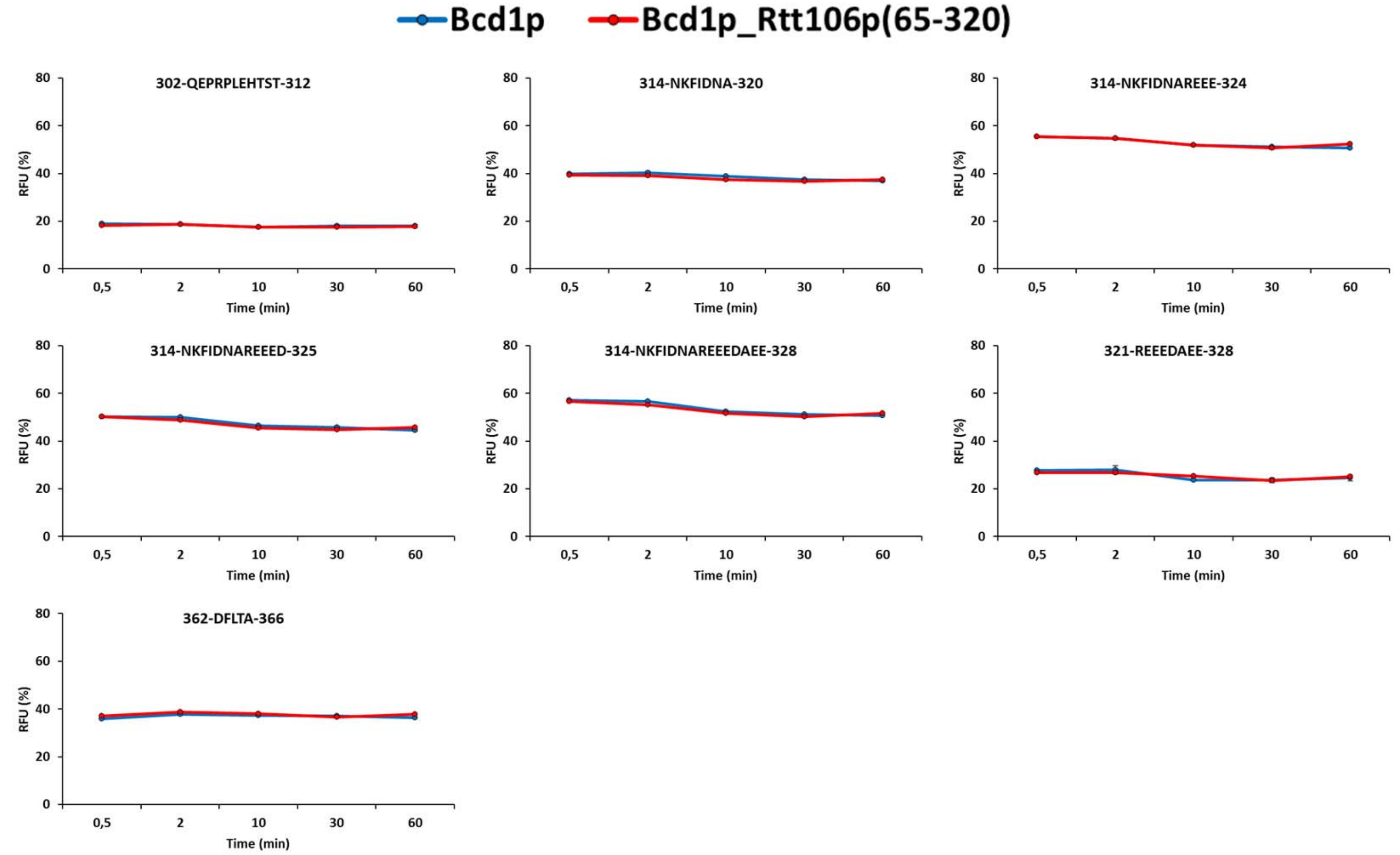

Fig. 11 HDX uptake plots of all identified and validated peptides of Bcd1p$_{FL}$ in Bcd1p$_{FL}$ alone (blue curve) and Bcd1p$_{FL}$:Rtt106p-M (red curve) states.

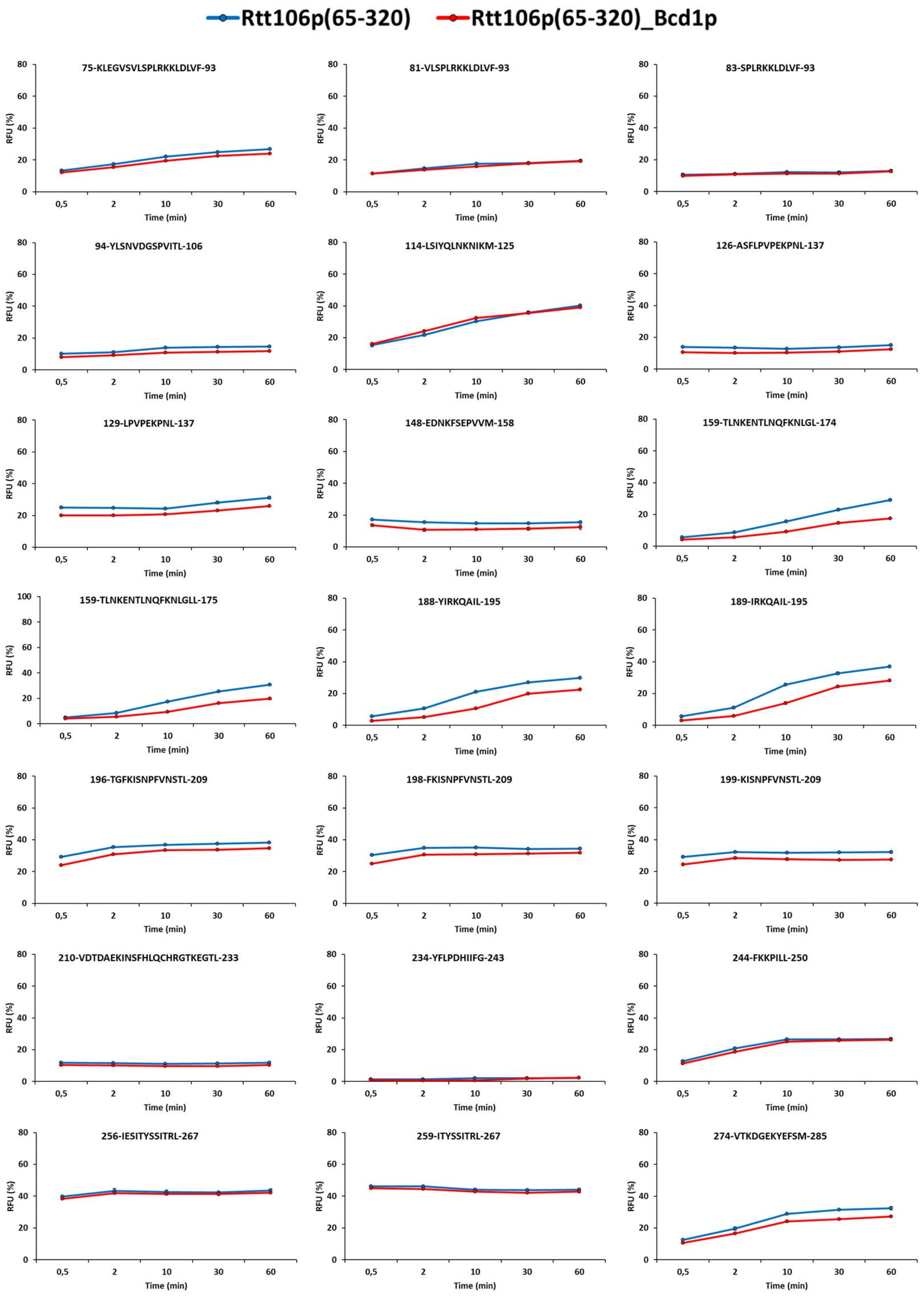
Rtt106p(65-320)
Rtt106p(65-320)_Bcd1p
75-KLEGVSVLSPLRKKLDLVF-93
81-VLSPLRKKLDLVF-93
83-SPLRKKLDLVF-93
94-YLSNVDGSPVITL-106
114-LSIYQLNKNIKM-125
126-ASFLPVPEKPNL-137
129-LPVPEKPNL-137
148-EDNKFSEPVVM-158
159-TLNKENTLNQFKNLGL-174
159-TLNKENTLNQFKNLGLL-175
188-YIRKQAIL-195
189-IRKQAIL-195
196-TGFKISNPFVNSTL-209
198-FKISNPFVNSTL-209
199-KISNPFVNSTL-209
210-VDTDAEKINSFHLQCHRGTKEGTL-233
234-YFLPDHIIFG-243
244-FKKPILL-250
256-IESITYSSITRL-267
259-ITYSSITRL-267
274-VTKDGEKYEFSM-285
RFU (%)
Time (min)
0,5
2
10
30
60

Fig. 12 HDX uptake plots of all identified and validated peptides of Rtt106p-M in Rtt106p-M alone (blue curve) and Bcd1p$_{FL}$:Rtt106p-M (red curve) states.

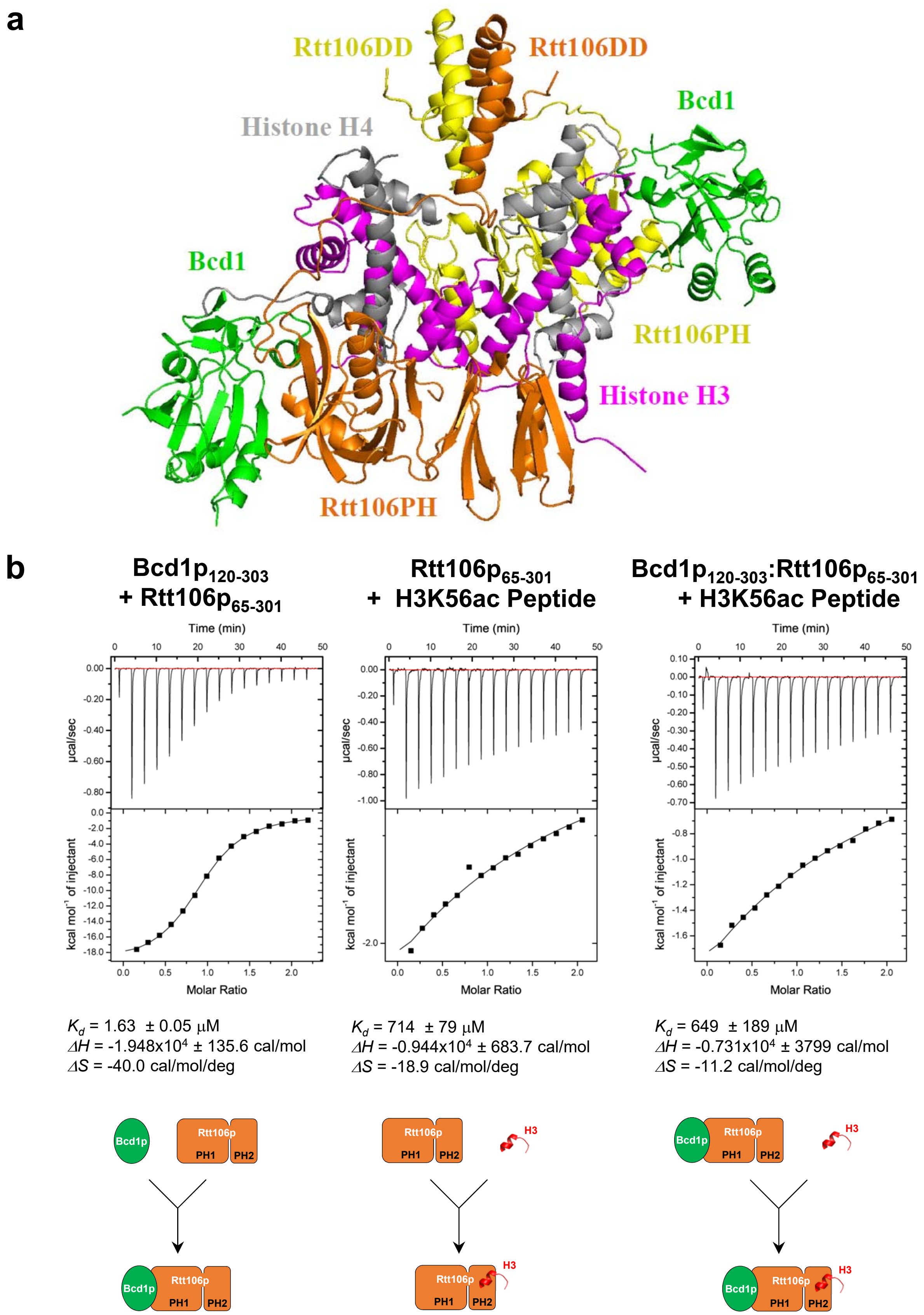

Fig. 13 a Structural model of the heterodimer Bcd1p:Rtt106p in complex with the $(H3{:}H4)_2$ tetramer. The model was built on the basis of the 3D model of the complex Rtt106p:$(H3{:}H4)_2$[13] and the present crystal structure of $Bcd1p_{120\text{-}303}$:$Rtt106p_{65\text{-}301}$ (our work). **b** Interaction analysis between $Rtt106p_{65\text{-}301}$ and H3K56ac peptide with or without $Bcd1p_{120\text{-}303}$ From the left to the right: ITC results between $Rtt106p_{65\text{-}301}$ and $Bcd1p_{120\text{-}303}$ (control), $Rtt106p_{65\text{-}301}$ and H3K56ac peptide and between the $Bcd1p_{120\text{-}303}$:$Rtt106p_{65\text{-}301}$ complex and H3K56ac peptide. These experiments were recorded at 293 K in buffer containing 10 mM NaPi at pH 7.5, 150 mM NaCl and 0.5 mM TCEP. The calculated affinities and thermodynamic parameters are indicated. Schematic representations of the experiments are shown at the bottom of each experiment.

a

```
Cns1        --GSENKK--------IMLESAMTLRNITNIKTHSPVELLNEGKIRLEDPMDFESQLIYP
Bcd1        RDSTECQRIIRRGVNCLMLPKGMQRS--------------SQNRSKWDKTMDLF--VWSV
              .:* ::        :** ..*                 .:.: : :. **:   :
Cns1        ALIMYPTQDEFDFVGEVSELTTVQELVDLVL------------------------EGPQE
Bcd1        EWILCPMQEKGEKKELFKHVSHRIKETDFLVQGMGKNVFQKCCEFYRLAGTSSCIEGEDG
              *: * *:: :    ...::   : .*:::                        ** :
Cns1        RFKKEGKENFTPKKVL-----------------------VFMETKAGGLIKAGKKLTFHD
Bcd1        SETKEERTQILQKSGLKFYTKTFPYNTTHIMDSKKLVELAIHEKCIGELLKNTTVIEFPT
              .** : ::  *. *                       .: *.  * *:*  . : *
Cns1        ILKKE-SPDVPLFDNALKIYIVPKVESEGWISKWDKQKALERRSV
Bcd1        IFVAMTEADL-----------------PEGYEVLHQE---------
            *:    . *:                 **:    ::
```

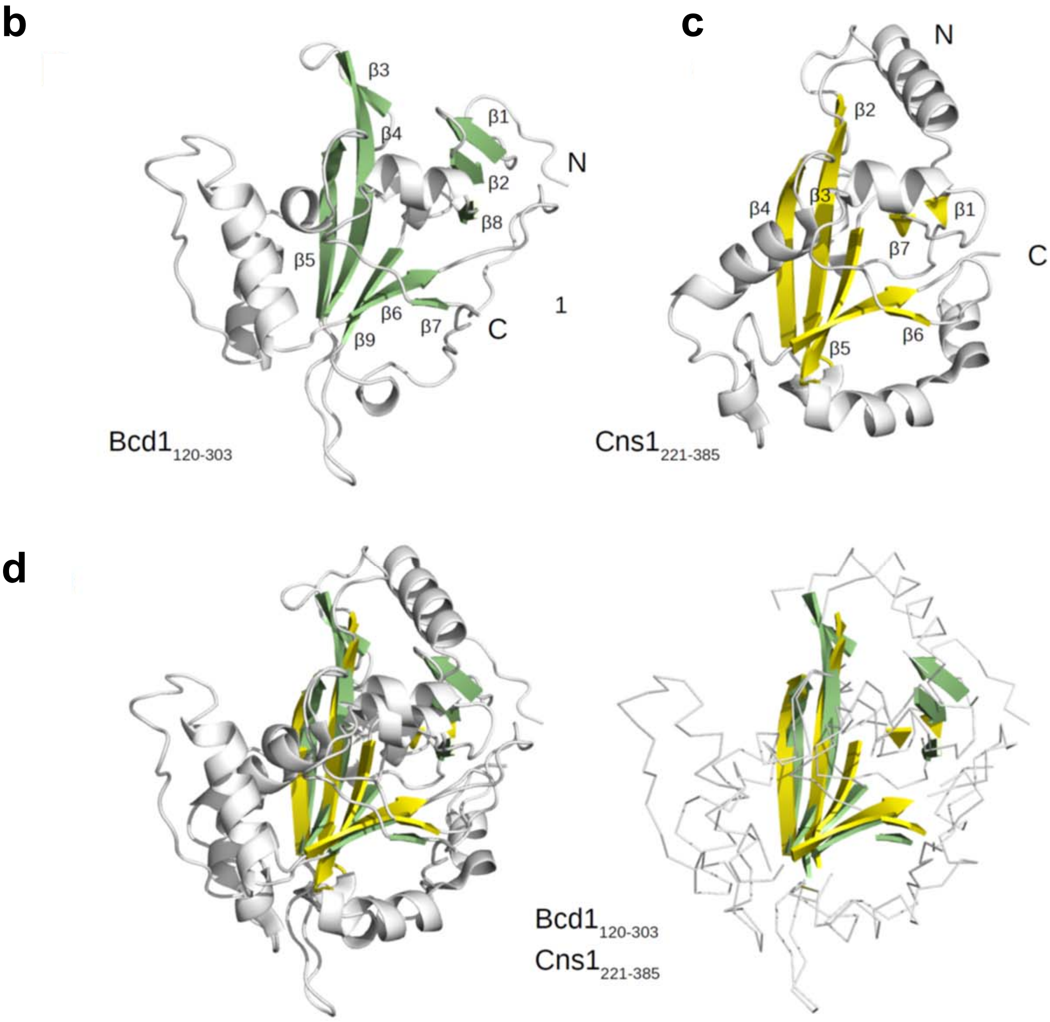

Fig. 14 Structural comparison of the wheel domains from Bcd1p and Cns1p. a Sequence alignment of Bcd1p$_{120\text{-}303}$ and Cns1p$_{221\text{-}385}$. Perfect matches, strongly similar and weakly similar residues are respectively indicated with an asterisk, a double dot and a dot. **b** Cartoon representation of the best NMR structure of Bcd1p$_{120\text{-}130}$ (entry PDB code 6NZ2 [https://www.rcsb.org/structure/6NZ2]). **c** Cartoon representation of the X-ray structure of Cns1p$_{221\text{-}385}$ (entry PDB code 6HFM [https://www.rcsb.org/structure/6HFM]). **d** 3D superimposition of Bcd1p$_{120\text{-}303}$ and Cns1p$_{221\text{-}385}$. On the right, structures are shown in cartoon mode. For more clarity, loops and helices in Bcd1p and Cns1p are shown in ribbon mode (on the left), whereas β-strands are displayed in cartoon mode.

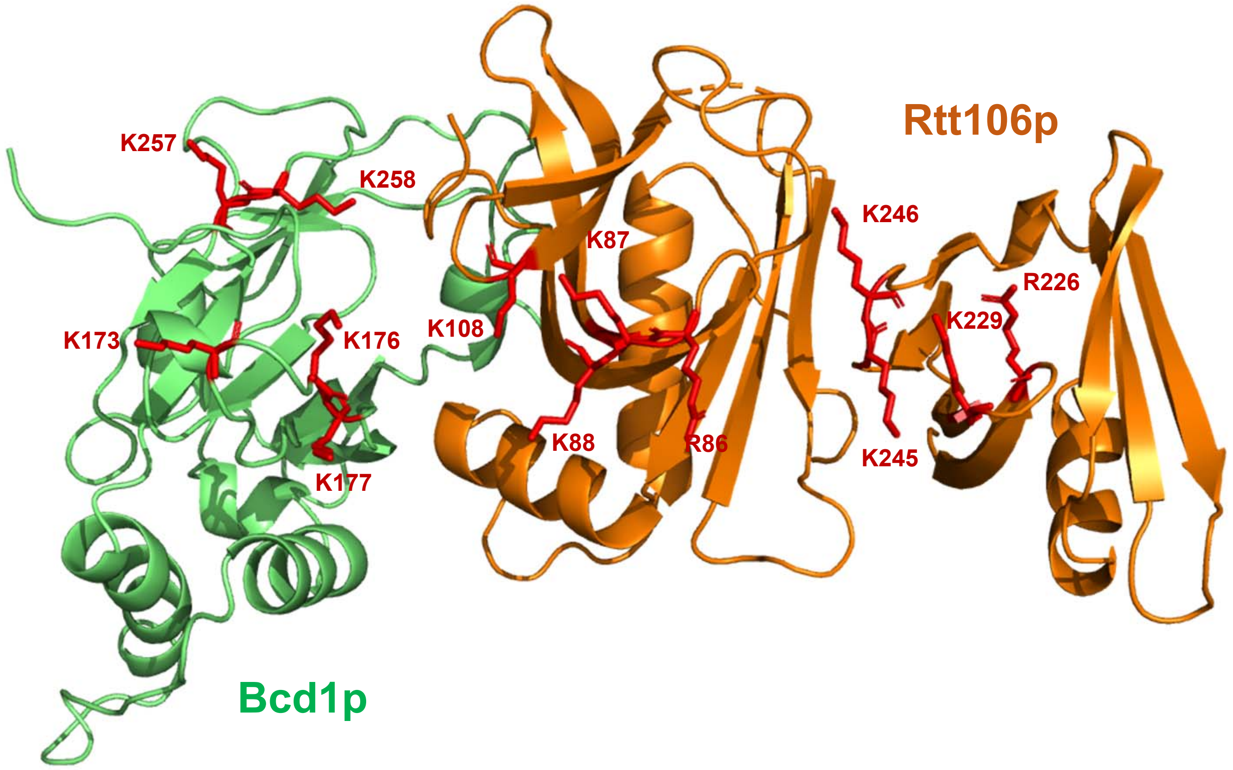

Fig. 15 The binding site for dsDNA at the surface of Rtt106p in the Rtt106p$_{65\text{-}301}$:Bcd1p$_{120\text{-}303}$ complex (entry PDB code 6THL [https://www.rcsb.org/structure/6THL]). Several conserved positively charged residues (in red) at the surface of Rtt106p form a positively charged ridge region that is responsible for dsDNA binding[14]. This positive patch continues on the Bcd1p molecular surface.

Supplementary Tables

Supplementary Table 1. Mass spectrometry analysis of gel bands from SDS-PAGE

Band	Protein	Sequence coverage	Number of unique peptides	Spectral count
1A	Bcd1_FL	78	52	2710
	His-Rtt106_(65-320)	51	13	50
1B	His-Rtt106_(65-320)	83	52	3667
	Bcd1_FL	55	31	158
1C	His_Bcd1_FL	68	52	2683
	Rtt106_(65-320)	84	21	143
1D	Rtt106_(65-320)	97	57	4036
	His_Bcd1_FL	54	35	178
1E	Bcd1_FL	69	43	1408
	His-Rtt106_FL	64	31	46
1F	His-Rtt106_FL	71	65	560
	Bcd1_FL	55	24	102
1G	His_Bcd1_FL	67	54	2524
	Rtt106_FL	74	56	939
1H	Rtt106_FL	78	68	3465
	His_Bcd1_FL	49	33	386

Supplementary Table 2. Results of the cross-linking experiments for the complex Bcd1p$_{FL}$:Rtt106p-M

Replicate	Ratio	Heavy/ Light	Cross–linked proteins	Cross–linked sites	Peptide 1	Peptide 2	m/z	Charge	M+H+	Calculated Mass	Deviation (ppm)	Retention time (sec)
	1:50	H	Bcd1p-Bcd1p	K16-K88	^{15}[YKBPR]^{19}	^{82}[MVHVQKMDAR]^{91}	693,692	3	2079,062	2079,059	1,5	1144,49
	1:50	L	Bcd1p-Bcd1p	K16-K88	^{15}[YKBPR]^{19}	^{82}[MVHVQKMDAR]^{91}	692,349	3	2075,033	2075,034	-0,7	1151.10
	1:50	H	Bcd1p-Bcd1p	K16-S106	^{15}[YKBPR]^{19}	^{97}[VLGPVGGHNSNFK]^{109}	548,291	4	2190,145	2190,142	1,3	1301.30
	1:50	L	Bcd1p-Bcd1p	K16-S106	^{15}[YKBPR]^{19}	^{97}[VLGPVGGHNSNFK]^{109}	729,377	3	2186,117	2186,117	-0,2	1310,32
	1:50	H	Bcd1p-Bcd1p	K16-K258	^{15}[YKBPR]^{19}	^{258}[KLVELAIHEK]^{267}	682,059	3	2044,164	2044,156	4,1	1674,91
	1:50	L	Bcd1p-Bcd1p	K16-K258	^{15}[YKBPR]^{19}	^{258}[KLVELAIHEK]^{267}	408,832	5	2040,131	2040,131	0,4	1678,35
	1:50	H	Bcd1p-Bcd1p	K80-K151	^{150}[SKWDK]^{154}	^{72}[DYNYLTQLKR]^{81}	706.710	3	2118,116	2118,116	-0,3	2538,07
	1:50	L	Bcd1p-Bcd1p	K80-K151	^{150}[SKWDK]^{154}	^{72}[DYNYLTQLKR]^{81}	705,368	3	2114,089	2114,091	-1.0	2550.45
	1:50	H	Bcd1p-Bcd1p	K87-K151	^{150}[SKWDK]^{154}	^{82}[MVHVQKMDAR]^{91}	673,685	3	2019,043	2019,045	-1.0	1500,29
	1:50	L	Bcd1p-Bcd1p	K87-K151	^{150}[SKWDK]^{154}	^{82}[MVHVQKMDAR]^{91}	672,344	3	2015,019	2015.020	-0,1	1507,08
	1:50	H	Bcd1p-Bcd1p	K140-K151	^{150}[SKWDK]^{154}	^{132}[GVNBLMLPKGMQR]^{144}	770,068	3	2308,188	2308,191	-1.0	2422,19
	1:50	L	Bcd1p-Bcd1p	K140-K151	^{150}[SKWDK]^{154}	^{132}[GVNBLMLPKGMQR]^{144}	768,727	3	2304,165	2304,166	-0,5	2433.80
	1:50	H	Bcd1p-Bcd1p	K151-K188	^{150}[SKWDK]^{154}	^{187}[IKETDFLVQGMGK]^{199}	568,308	4	2270,209	2270,204	2,4	2496.24
	1:50	L	Bcd1p-Bcd1p	K151-K188	^{150}[SKWDK]^{154}	^{187}[IKETDFLVQGMGK]^{199}	756,064	3	2266,177	2266,178	-0,3	2509,23
1	1:50	H	Rtt106p-Bcd1p	S177-K151	^{171}[NLGLLDSNVTDFEK]^{184}	^{150}[SKWDK]^{154}	790.411	3	2369,218	2369,217	0,2	3028,83
	1:50	L	Rtt106p-Bcd1p	S177-K151	^{171}[NLGLLDSNVTDFEK]^{184}	^{150}[SKWDK]^{154}	789,070	3	2365,194	2365,192	0,9	3038,08
	1:100	H	Bcd1p-Bcd1p	K16-K62	^{15}[YKBPR]^{19}	^{57}[QADDDKHER]^{65}	659,984	3	1977,937	1977,939	-0,9	556,97
	1:100	L	Bcd1p-Bcd1p	K16-K62	^{15}[YKBPR]^{19}	^{57}[QADDDKHER]^{65}	658,643	3	1973,913	1973,913	-0,1	560,22
	1:100	H	Bcd1p-Bcd1p	K16-K88	^{15}[YKBPR]^{19}	^{82}[MVHVQKMDAR]^{91}	693,692	3	2079,061	2079,060	0,5	1139,98
	1:100	L	Bcd1p-Bcd1p	K16-K88	^{15}[YKBPR]^{19}	^{82}[MVHVQKMDAR]^{91}	692.350	3	2075,034	2075,034	0,1	1147,61
	1:100	H	Bcd1p-Bcd1p	K16-K151	^{15}[YKBPR]^{19}	^{150}[SKWDK]^{154}	509,937	3	1527,796	1527,793	2,3	966,86
	1:100	L	Bcd1p-Bcd1p	K16-K151	^{15}[YKBPR]^{19}	^{150}[SKWDK]^{154}	508,594	3	1523,768	1523,768	0,6	968,52
	1:100	H	Bcd1p-Bcd1p	K16-K258	^{15}[YKBPR]^{19}	^{258}[KLVELAIHEK]^{267}	409,637	5	2044.158	2044,156	1,1	1649,36
	1:100	L	Bcd1p-Bcd1p	K16-K258	^{15}[YKBPR]^{19}	^{258}[KLVELAIHEK]^{267}	408,832	5	2040,132	2040,131	0,6	1657,68
	1:100	H	Bcd1p-Bcd1p	K62-T77/K80	^{57}[QADDDKHER]^{65}	^{72}[DYNYLTQLKR]^{81}	856,759	3	2568,262	2568,263	-0,2	1876,36
	1:100	L	Bcd1p-Bcd1p	K62-T77/K80	^{57}[QADDDKHER]^{65}	^{72}[DYNYLTQLKR]^{81}	855,417	3	2564,236	2564,238	-0,8	1893,61
	1:100	H	Bcd1p-Bcd1p	K151-K188	^{150}[SKWDK]^{154}	^{187}[IKETDFLVQGmGK]^{199}	762,737	3	2286,198	2286,199	-0,5	2159,75
	1:100	L	Bcd1p-Bcd1p	K151-K188	^{150}[SKWDK]^{154}	^{187}[IKETDFLVQGmGK]^{199}	761,396	3	2282.174	2282,174	0,4	2174,69
	1:100	H	Rtt106p-Bcd1p	S177-K151	^{171}[NLGLLDSNVTDFEK]^{184}	^{150}[SKWDK]^{154}	790,410	3	2369,217	2369,217	-0,2	3035,31
	1:100	L	Rtt106p-Bcd1p	S177-K151	^{171}[NLGLLDSNVTDFEK]^{184}	^{150}[SKWDK]^{154}	789.069	3	2365,193	2365,192	0,5	3046,35
	1:50	H	Bcd1p-Bcd1p	K16-K88	^{15}[YKBPR]^{19}	^{82}[mVHVQKMDAR]^{91}	699,023	3	2095,055	2095,055	-0,01	915,29
	1:50	L	Bcd1p-Bcd1p	K16-K88	^{15}[YKBPR]^{19}	^{82}[mVHVQKMDAR]^{91}	697,681	3	2091,029	2091,030	-0,1	920,91
	1:50	H	Bcd1p-Bcd1p	K16-S106	^{15}[YKBPR]^{19}	^{97}[VLGPVGGHNSNFK]^{109}	548,292	4	2190,146	2190,143	1,4	1207,99
	1:50	L	Bcd1p-Bcd1p	K16-S106	^{15}[YKBPR]^{19}	^{97}[VLGPVGGHNSNFK]^{109}	547,285	4	2186,120	2186,118	0,9	1217,83
	1:50	H	Bcd1p-Bcd1p	K16-K258	^{15}[YKBPR]^{19}	^{258}[KLVELAIHEK]^{267}	409,637	5	2044,157	2044,156	0,4	1549,04
	1:50	L	Bcd1p-Bcd1p	K16-K258	^{15}[YKBPR]^{19}	^{258}[KLVELAIHEK]^{267}	408,832	5	2040,131	2040,131	0,1	1554,43
	1:50	H	Bcd1p-Bcd1p	K80-K151	^{150}[SKWDK]^{154}	^{72}[DYNYLTQLKR]^{81}	706,711	3	2118,118	2118,117	0,5	2442,31
	1:50	L	Bcd1p-Bcd1p	K80-K151	^{150}[SKWDK]^{154}	^{72}[DYNYLTQLKR]^{81}	705,369	3	2114,093	2114,092	0,7	2454,23
	1:50	H	Bcd1p-Bcd1p	K87-K151	^{150}[SKWDK]^{154}	^{82}[MVHVQKMDAR]^{91}	673,687	3	2019,045	2019,045	0,01	1399,26
	1:50	L	Bcd1p-Bcd1p	K87-K151	^{150}[SKWDK]^{154}	^{82}[MVHVQKMDAR]^{91}	672,346	3	2015,023	2015,020	1,3	1406,37
	1:50	H	Bcd1p-Bcd1p	K140-K151	^{150}[SKWDK]^{154}	^{132}[GVNBLMLPKGMQR]^{144}	577,806	4	2308,201	2308,191	4,2	2285,52
	1:50	L	Bcd1p-Bcd1p	K140-K151	^{150}[SKWDK]^{154}	^{132}[GVNBLMLPKGMQR]^{144}	576,799	4	2304,172	2304,166	2,7	2297,39
	1:50	H	Bcd1p-Bcd1p	K151-K188	^{150}[SKWDK]^{154}	^{187}[IKETDFLVQGMGK]^{199}	757,406	3	2270,203	2270,204	-0,3	2422,43
	1:50	L	Bcd1p-Bcd1p	K151-K188	^{150}[SKWDK]^{154}	^{187}[IKETDFLVQGMGK]^{199}	756,063	3	2266,174	2266,179	-2,1	2439,90
2	1:50	H	Rtt106p-Bcd1p	S177-K151	^{171}[NLGLLDSNVTDFEK]^{184}	^{150}[SKWDK]^{154}	790,412	3	2369,221	2369,217	1,4	2894,23
	1:50	L	Rtt106p-Bcd1p	S177-K151	^{171}[NLGLLDSNVTDFEK]^{184}	^{150}[SKWDK]^{154}	789,069	3	2365,193	2365,192	0,4	2903,69
	1:100	H	Bcd1p-Bcd1p	K16-K62	^{15}[YKBPR]^{19}	^{57}[QADDDKHER]^{65}	659,984	3	1977,939	1977,939	0,03	401,83
	1:100	L	Bcd1p-Bcd1p	K16-K62	^{15}[YKBPR]^{19}	^{57}[QADDDKHER]^{65}	494,234	4	1973,914	1973,913	0,5	401,76
	1:100	H	Bcd1p-Bcd1p	K16-K88	^{15}[YKBPR]^{19}	^{82}[MVHVQKmDAR]^{91}	699,023	3	2095,054	2095,055	-0,4	839,91
	1:100	L	Bcd1p-Bcd1p	K16-K88	^{15}[YKBPR]^{19}	^{82}[MVHVQKmDAR]^{91}	697,681	3	2091,028	2091,030	-1,0	930,05
	1:100	H	Bcd1p-Bcd1p	K16-K151	^{15}[YKBPR]^{19}	^{150}[SKWDK]^{154}	382,703	4	1527,792	1527,793	-0,7	914,83
	1:100	L	Bcd1p-Bcd1p	K16-K151	^{15}[YKBPR]^{19}	^{150}[SKWDK]^{154}	381,697	4	1523,767	1523,768	-0,5	924,37
	1:100	H	Bcd1p-Bcd1p	K16-K258	^{15}[YKBPR]^{19}	^{258}[KLVELAIHEK]^{267}	682,058	3	2044,160	2044,156	2,0	1559,56
	1:100	L	Bcd1p-Bcd1p	K16-K258	^{15}[YKBPR]^{19}	^{258}[KLVELAIHEK]^{267}	408,832	5	2040,133	2040,131	0,7	1565,29
	1:100	H	Bcd1p-Bcd1p	K62-T77/K80	^{57}[QADDDKHER]^{65}	^{72}[DYNYLTQLKR]^{81}	856,760	3	2568,265	2568,263	0,8	1790,24
	1:100	L	Bcd1p-Bcd1p	K62-T77/K80	^{57}[QADDDKHER]^{65}	^{72}[DYNYLTQLKR]^{81}	855,417	3	2564,238	2564,238	0,1	1803,40
	1:100	H	Bcd1p-Bcd1p	K151-K188	^{150}[SKWDK]^{154}	^{187}[IKETDFLVQGMGK]^{199}	757,406	3	2270,205	2270,204	0,3	2376,95
	1:100	L	Bcd1p-Bcd1p	K151-K188	^{150}[SKWDK]^{154}	^{187}[IKETDFLVQGMGK]^{199}	756,065	3	2266,181	2266,179	0,8	2391,04
	1:100	H	Rtt106p-Bcd1p	S177-K151	^{171}[NLGLLDSNVTDFEK]^{184}	^{150}[SKWDK]^{154}	790,412	3	2369,223	2369,217	2,3	2902,24
	1:100	L	Rtt106p-Bcd1p	S177-K151	^{171}[NLGLLDSNVTDFEK]^{184}	^{150}[SKWDK]^{154}	789,070	3	2365,196	2365,192	1,7	2907,60

Supplementary Table 3. Oligonucleotides used in this study

Oligonucleotide	Sequence	Reference
U3 -800nts^{for}	AACAGTTGTCCAACAAGTGC	This study
U3 -800nts^{rev}	AATGGCTGAATCCCATAGAC	This study
U3 -400nts^{for}	CCATGGATGGGTAAAGTCAT	This study
U3 -400nts^{rev}	TCAGAATTTGTTCTGCCTGG	This study
U3 prom^{for}	CACATACAGCGCCTTAAGG	This study
U3 prom^{rev}	GAAGTACGTCGACACAGAT	This study
U3 intron^{for}	AATATACCCCAAACATTTTACCC	This study
U3 intron^{rev}	TGTAGAATGTGTTAGTCAAAAGCTG	This study
U3 exon 2^{for}	GAGCCACTGAATCCAAC	[15]
U3 exon 2^{rev}	GTACATAGGATGGGTCA	[15]
U3 term^{for}	GCTTTGCCGTTGCATTTGT	This study
U3 term^{rev}	CTTACACACCAACTAATCCA	This study
U3 +400nts^{for}	CCTATTGTAGGAATTGTGGC	This study
U3 +400nts^{rev}	CTTTCGTCAATGAAGTGACC	This study
U3 +800nts^{for}	CCATTACAGATCATTGGCTC	This study
U3 +800nts^{rev}	GCGTTGATCACAGTCTGTTT	This study
U14^{for}	TCACGGTGATGAAAGACTGG	[15]
U14^{rev}	AAGAGCGGTCACCGAGAGTA	[15]
U14 3'ext^{rev}	GATACTACAGTATACGATCACTC	[15]
snR52^{for}	TGAATGACATTAGCGTGAACAA	[15]
snR52^{rev}	TTCAGAAGGAAGGCAACATAAG	[15]
snR54^{for}	CCGAAAAAGGTGCGAATATG	[15]
snR54^{rev}	AATGCGCTTTTTAATCATCCA	[15]
snR32^{for}	TGCAGGGAATGACTAAAAGCC	[15]
snR32^{rev}	TCAGAAATGATCAACACCAGGC	[15]
Chr.V^{for}	GCAATCAACATCTGAAGAAAAGAAAGTAGT	[15]
Chr.V^{rev}	CATAATCTGCGTAAAAATGGCGTAAAT	[15]
snR54^{for}	CCGAAAAAGGTGCGAATATG	This study
snR54^{rev}	AATGCGCTTTTTAATCATCCA	This study
Alg9^{for}	TGTCACGGATAGTGGCTTTG	[16]
Alg9^{rev}	TACCATTCACGTCCCGTACA	[16]
HTB1 downstream ^{for}	CGAAACTTCAGAGCATTGGC	[17]
HTB1 downstream ^{rev}	GGGTTCAATCTCCAAGGCAT	[17]
HTA/B1 prom ^{for}	ATAGTTAACGACCCAACCGCGT	[17]
HTA/B1 prom ^{rev}	ACGGGCGTTTCTTCAACAACGA	[17]
HTA1 downstream ^{for}	AGGTTCATTGGGCACTGTTG	[17]
HTA1 downstream ^{rev}	ACAGTTCTCCGTGACAGGAT	[17]
HTA/B2 prom ^{for}	AATGGTAGCACGTCGCGTTT	[17]
HTA/B2 prom ^{rev}	TGACGGCAAGTGTCTCACTGTT	[17]
HMR a1 ^{for}	TGGATGATATTTGTAGTATGGCGGA	[17]
HMR a1 ^{rev}	TCCCTTTGGGCTCTTCTCTT	[17]
KanMx ^{for}	ATTGACCACACCTCTACCGGGACATGGAGGCCCA GAATACCCTCCTTGACAGTCTTGACGTGCGC	This study
KanMx ^{rev}	GCGCACGTCAAGACTGTCAAGGAGGGTATTCTG GGCCTCCATGTCCCGGTAGAGGTGTGGTCAAT	This study
Bcd1 1-115 ^{for}	GTGGGCGGCCACAACTCTAATTTCAAGAAGAGAA GATACGATATATGAGCGAATTTCTTATGATTTAT	This study
Bcd1 1-115 ^{rev}	CCTAATTATCCTCTGACACTCCGTGCTATCACGAT CATCCTCATCCAGTATAGCGACCAGCATTC	This study

Supplementary Table 4. Plasmids used in this study

Plasmid	Characteristics	Reference
S. cerevisiae		
p416GPD	GPD promoter, *URA3*, Amp^r	[18]
p416GPD::FLAG-Bcd1p	FLAG-Bcd1p	This study
p413TEF	TEF promoter, *HIS3*, Amp^r	[18]
p413TEF::FLAG-Rtt106p	FLAG-Rtt106p	This study
pGBKT7::Gal4-BD-Bcd1p	ADH promoter, *TRP1*, Kan^r, BD-Bcd1p	This study
pACT2::GAL4-AD	ADH promoter, *LEU2*, Amp^r	This study
pACT2::GAL4-AD-Rtt106p	AD-Rtt106p	This study
pACT2::GAL4-AD-Rtt106-Mp	AD-Rtt106-Mp	This study
pACT2::GAL4-AD-Spt16-Mp	AD-Spt16-Mp	This study
pACT2::GAL4-AD-Pop3p-Mp	AD-Pop3-Mp	This study
pFA6a-KanMX6-pGAL1-3HA	Kan^r - Amp^r	This study
E. coli		
pnCS::Bcd1p	Bcd1p	This study
pnCS ::Rtt106-Mp	Rtt106-Mp	This study
pnCS ::Rtt106p	Rtt106p	This study
pnEA-3cH::HIS-Bcd1p	HIS-Bcd1p	This study
pnEA-3cH::HIS-Rtt106-Mp	HIS-Rtt106-Mp	This study
pnEA-3cH::HIS-Rtt106p	HIS-Rtt106p	This study

Supplementary Table 5. Yeast strains used in this study

Strain	Background	Genotype	Reference
BY4741	S288C	*MATa ; his3Δ1; leu2Δ0; met15Δ0; ura3Δ0*	[19]
rtt106Δ	S288C	*MATa ; his3Δ1; leu2Δ0; met15Δ0; ura3Δ0; ynl206cΔ::kanMX4*	This study
BCD1-TAP	S288C	*MATa ; ade2 ; arg4 ; leu2–3,112 ; trp1–289 ; ura3–52; HIS3::TAP-YHR040w*	[20]
BCD1-TAP; rsa1Δ	S288C	*MATa ; his3Δ1; leu2Δ0; met15Δ0; ura3Δ0 ; HIS3::TAP-YHR040w ; YPL193w::LEU2*	This study
BCD1-TAP; pih1Δ	S288C	*MATa ; his3Δ1; leu2Δ0; met15Δ0; ura3Δ0 ; HIS3::TAP-YHR040w ; YHR034c::LEU2*	This study
BCD1-TAP; rtt106Δ	S288C	*MATa ; ade2 ; arg4 ; leu2–3,112 ; trp1–289 ; ura3–52; HIS3::TAP-YHR040w; ynl206cΔ::kanMX4*	This study
GAL1::3HA-BCD1	S288C	*MATa ; his3Δ1; leu2Δ0; met15Δ0; ura3Δ0 ; pGAL1-3HA::kanMX6-YHR040w*	This study
$BCD1_{1\text{-}115}$	S288C	*MATa ; his3Δ1; leu2Δ0; met15Δ0; ura3Δ0 ; kanMX6-YHR040w 1-115*	This study
RTT106-TAP	S288C	*MATa ; ade2 ; arg4 ; leu2–3,112 ; trp1–289 ; ura3–52; HIS3::TAP-YNL206C*	This study
RTT106-TAP; GAL1::3HA-BCD1	S288C	*MATa ; ade2 ; arg4 ; leu2–3,112 ; trp1–289 ; ura3–52; HIS3::TAP-YNL206C ; pGAL1-3HA::kanMX6-YHR040w*	This study
RTT106-TAP; GAL1::3HA-BCD1; rsa1Δ	S288C	*MATa ; ade2 ; arg4 ; leu2–3,112 ; trp1–289 ; ura3–52; HIS3::TAP-YNL206C ; pGAL1-3HA::kanMX6-YHR040w ; YPL193w::LEU2*	This study
Y187		*MATα, gal4Δ, gal80Δ, ade2-101, his3-200, leu2-3, 112, lys2-801, trp1-901, ura3-52, URA3::Gal1UASGAL1TATA-lacZ, LYS2::GAL1UASHisTATA-HIS3*	
Y190		*MATa gal4Δ, gal80Δ, ade2-101, his3-200, leu2-3, 112, lys2-801, trp1-901, ura3-52, URA3::Gal1UASGAL1TATA-lacZ, LYS2::GAL1UASHisTATA-HIS3*	

Supplementary References

1. Jeronimo, C. *et al.* Systematic analysis of the protein interaction network for the human transcription machinery reveals the identity of the 7SK capping enzyme. *Molecular cell* **27**, 262-274, doi:10.1016/j.molcel.2007.06.027 (2007).
2. Boulon, S. *et al.* HSP90 and its R2TP/Prefoldin-like cochaperone are involved in the cytoplasmic assembly of RNA polymerase II. *Molecular cell* **39**, 912-924, doi:10.1016/j.molcel.2010.08.023 (2010).
3. Horejsi, Z. *et al.* Phosphorylation-dependent PIH1D1 interactions define substrate specificity of the R2TP cochaperone complex. *Cell reports* **7**, 19-26, doi:10.1016/j.celrep.2014.03.013 (2014).
4. Horejsi, Z. *et al.* CK2 phospho-dependent binding of R2TP complex to TEL2 is essential for mTOR and SMG1 stability. *Molecular cell* **39**, 839-850, doi:10.1016/j.molcel.2010.08.037 (2010).
5. Izumi, N., Yamashita, A., Hirano, H. & Ohno, S. Heat shock protein 90 regulates phosphatidylinositol 3-kinase-related protein kinase family proteins together with the RUVBL1/2 and Tel2-containing co-factor complex. *Cancer science* **103**, 50-57, doi:10.1111/j.1349-7006.2011.02112.x (2012).
6. Takai, H., Xie, Y., de Lange, T. & Pavletich, N. P. Tel2 structure and function in the Hsp90-dependent maturation of mTOR and ATR complexes. *Genes & development* **24**, 2019-2030, doi:10.1101/gad.1956410 (2010).
7. Bizarro, J. *et al.* NUFIP and the HSP90/R2TP chaperone bind the SMN complex and facilitate assembly of U4-specific proteins. *Nucleic acids research* **43**, 8973-8989, doi:10.1093/nar/gkv809 (2015).
8. Cloutier, P. *et al.* R2TP/Prefoldin-like component RUVBL1/RUVBL2 directly interacts with ZNHIT2 to regulate assembly of U5 small nuclear ribonucleoprotein. *Nature communications* **8**, 15615, doi:10.1038/ncomms15615 (2017).
9. Malinova, A. *et al.* Assembly of the U5 snRNP component PRPF8 is controlled by the HSP90/R2TP chaperones. *The Journal of cell biology* **216**, 1579-1596, doi:10.1083/jcb.201701165 (2017).
10. Cloutier, P. *et al.* High-resolution mapping of the protein interaction network for the human transcription machinery and affinity purification of RNA polymerase II-associated complexes. *Methods* **48**, 381-386, doi:10.1016/j.ymeth.2009.05.005 (2009).
11. Ruotolo, B. T., Benesch, J. L., Sandercock, A. M., Hyung, S. J. & Robinson, C. V. Ion mobility-mass spectrometry analysis of large protein complexes. *Nature protocols* **3**, 1139-1152, doi:10.1038/nprot.2008.78 (2008).
12. Sievers, F. *et al.* Fast, scalable generation of high-quality protein multiple sequence alignments using Clustal Omega. *Molecular systems biology* **7**, 539, doi:10.1038/msb.2011.75 (2011).
13. Su, D. *et al.* Structural basis for recognition of H3K56-acetylated histone H3-H4 by the chaperone Rtt106. *Nature* **483**, 104-107, doi:10.1038/nature10861 (2012).
14. Liu, Y. *et al.* Structural analysis of Rtt106p reveals a DNA binding role required for heterochromatin silencing. *The Journal of biological chemistry* **285**, 4251-4262, doi:10.1074/jbc.M109.055996 (2010).
15. Paul, A. *et al.* Bcd1p controls RNA loading of the core protein Nop58 during C/D box snoRNP biogenesis. *Rna*, doi:10.1261/rna.067967.118 (2019).
16. Teste, M. A., Duquenne, M., Francois, J. M. & Parrou, J. L. Validation of reference genes for quantitative expression analysis by real-time RT-PCR in Saccharomyces cerevisiae. *BMC Mol Biol* **10**, 99, doi:10.1186/1471-2199-10-99 (2009).
17. Zunder, R. M. & Rine, J. Direct interplay among histones, histone chaperones, and a chromatin boundary protein in the control of histone gene expression. *Molecular and cellular biology* **32**, 4337-4349, doi:10.1128/MCB.00871-12 (2012).

18. Mumberg, D., Muller, R. & Funk, M. Yeast vectors for the controlled expression of heterologous proteins in different genetic backgrounds. *Gene* **156**, 119-122, doi:10.1016/0378-1119(95)00037-7 (1995).
19. Brachmann, C. B. *et al.* Designer deletion strains derived from Saccharomyces cerevisiae S288C: a useful set of strains and plasmids for PCR-mediated gene disruption and other applications. *Yeast* **14**, 115-132, doi:10.1002/(SICI)1097-0061(19980130)14:2<115::AID-YEA204>3.0.CO;2-2 (1998).
20. Gavin, A. C. *et al.* Functional organization of the yeast proteome by systematic analysis of protein complexes. *Nature* **415**, 141-147, doi:10.1038/415141a (2002).
