# Supplementary material for: The box C/D snoRNP assembly factor Bcd1 interacts with the histone chaperone Rtt106 and controls its transcription dependent activity
Source: Nat Commun. 2021 Mar 25;12:1859. doi: 10.1038/s41467-021-22077-4 (PMC7994586; doi:10.1038/s41467-021-22077-4)
Supplement: Supplementary file 2 — Reporting Summary [file 41467_2021_22077_MOESM2_ESM.pdf]

nature research

Corresponding author(s): Bruno CHARPENTIER
Last updated by author(s): Feb 24, 2021

Reporting Summary

Nature Research wishes to improve the reproducibility of the work that we publish. This form provides structure for consistency and transparency in reporting. For further information on Nature Research policies, see Authors & Referees and the Editorial Policy Checklist.

Statistics

For all statistical analyses, confirm that the following items are present in the figure legend, table legend, main text, or Methods section.

n/a	Confirmed	
☐	☒	The exact sample size (*n*) for each experimental group/condition, given as a discrete number and unit of measurement
☐	☒	A statement on whether measurements were taken from distinct samples or whether the same sample was measured repeatedly
☐	☒	The statistical test(s) used AND whether they are one- or two-sided<br>*Only common tests should be described solely by name; describe more complex techniques in the Methods section.*
☒	☐	A description of all covariates tested
☒	☐	A description of any assumptions or corrections, such as tests of normality and adjustment for multiple comparisons
☐	☒	A full description of the statistical parameters including central tendency (e.g. means) or other basic estimates (e.g. regression coefficient) AND variation (e.g. standard deviation) or associated estimates of uncertainty (e.g. confidence intervals)
☐	☒	For null hypothesis testing, the test statistic (e.g. *F*, *t*, *r*) with confidence intervals, effect sizes, degrees of freedom and *P* value noted<br>*Give P values as exact values whenever suitable.*
☒	☐	For Bayesian analysis, information on the choice of priors and Markov chain Monte Carlo settings
☒	☐	For hierarchical and complex designs, identification of the appropriate level for tests and full reporting of outcomes
☒	☐	Estimates of effect sizes (e.g. Cohen's *d*, Pearson's *r*), indicating how they were calculated

Our web collection on statistics for biologists contains articles on many of the points above.

Software and code

Policy information about availability of computer code

Data collection	TopSpin 3.2; CYANA 3.97; TALOS-N; AMPS-NMR; XDS; PHASER; COOT; CCP4 suite; REFMACS; ProteinLynx Global Serveur 2.5.3; MSConvert.<br>Collection of X-ray data: XDS; TRUNCATE in the CCP4 suite.
Data analysis	GIM data analysis: g:Profiler [https://biit.cs.ut.ee/gprofiler/gost].<br>Mass spectrometry data analysis: Mascot 2.6.2; PyMOL 2.2.0 [https//pymol.org/2/]; MassLynx 4.1; Mobcal; MEMHDX [http://memhdx.c3bi.pasteur.fr/]; StavroX software v.3.6.6; Qualbrowser Thermo X-Calibur 3.0.63; DynamX 3.0; Fusion-Capt Advance Solo 4; AnalystTF 1.6 [https://sciex.com/content/SCIEX/na/us/en/products/software/analyst-890 software.html]; Proline pipeline [http://www.profiproteomics.fr/proline/]; MSConvert.<br>ITC data analysis: Origin7 software.<br>NMR calculations: AMPS-NMR [http://pyenmr.cerm.unifi.it/access/index/amps-nmr]; PyMOL 2.2.0 [https//pymol.org/2/].<br>Crystal structure determination: PROCHECK; PyMOL 2.2.0 [https//pymol.org/2/].<br>3D structure analysis: Dali server http://ekhidna.biocenter.helsinki.fi/dali_server].<br>Quantification of wertern blots: Fusion-Capt Advance Solo 4.

For manuscripts utilizing custom algorithms or software that are central to the research but not yet described in published literature, software must be made available to editors/reviewers. We strongly encourage code deposition in a community repository (e.g. GitHub). See the Nature Research guidelines for submitting code & software for further information.

Data

Policy information about availability of data

All manuscripts must include a data availability statement. This statement should provide the following information, where applicable:

- Accession codes, unique identifiers, or web links for publicly available datasets
- A list of figures that have associated raw data
- A description of any restrictions on data availability

- GIM data that support the findings of this study have been deposited in NCBI GEO with the accession code GSE118550 [https://www.ncbi.nlm.nih.gov/geo/query/acc.cgi?acc=GSE118550].
- NMR data have been deposited in the Biological Magnetic Resonance Data Bank (access code 30570 [https://bmrb.io/data_library/summary/index.php?bmrbId=30570]) and Protein Data Bank (entry code 6NZ2 [https://www.rcsb.org/structure/6NZ2]).
- X ray data have been deposited in the Protein Data Bank (entry code 6THL [https://www.rcsb.org/structure/6THL]).
- The mass spectrometry and proteomic data that support the findings of this study have been deposited to the ProteomeXchange Consortium via the PRIDE [https://www.ebi.ac.uk/pride/archive/] partner repository with the dataset identifier PXD023434. This submission includes all HDX-MS raw data files, Cluster and State data files (csv output files from DynamX), H/D plots ppt file, XL-MS raw data files, a Excel file summarizing MS information from all validated XL peptides, a FASTA file of the sequences used in structural MS experiments, Gel bands analyses raw data files, FASTA file used for Mascot research and excel file summarizing MS information of all peptides and proteins identified.

The authors declare that all other data supporting the findings of this study are available within the paper and its supplementary information files.
Figure 1 has associated raw data deposited in NCBI GEO (see GIM data above) and in the Source data file.
Graphs shown in Figures 3, 4 and Supplementary Figure 2 have associated raw data, which are collected in the Source data file.
The 3D structures shown in Figures 5 & 6 are deposited in PDB (see above).

Field-specific reporting

Please select the one below that is the best fit for your research. If you are not sure, read the appropriate sections before making your selection.

☒ Life sciences ☐ Behavioural & social sciences ☐ Ecological, evolutionary & environmental sciences

For a reference copy of the document with all sections, see nature.com/documents/nr-reporting-summary-flat.pdf

Life sciences study design

All studies must disclose on these points even when the disclosure is negative.

Sample size	A minimum of 3 independent replicates was used for RT-qPCR, ChIP-qPCR, RIP, Western-blot and co-IP data, following conventional experimental design in the field.
Data exclusions	No data were excluded from the analyses.
Replication	RT-qPCR, ChIP-qPCR, RIP, Western-blot and co-IP results were consistently replicated at least 3 times using independent populations of wild-type or genetically-engineered, syngenic yeast cells.
Randomization	Randomization was not used since there are no experimental groups.
Blinding	Blinding was not necessary because the results are quantitative and did not require subjective judgment or interpretation, and was not possible due to limitation in people involved in each team of the consortium. Most experimental processes starting from strain generation to sample collection and analyses were done by one person at a time.

Reporting for specific materials, systems and methods

We require information from authors about some types of materials, experimental systems and methods used in many studies. Here, indicate whether each material, system or method listed is relevant to your study. If you are not sure if a list item applies to your research, read the appropriate section before selecting a response.

Materials & experimental systems

n/a	Involved in the study	
☐	☒	Antibodies
☒	☐	Eukaryotic cell lines
☒	☐	Palaeontology
☒	☐	Animals and other organisms
☒	☐	Human research participants
☒	☐	Clinical data

Methods

n/a	Involved in the study	
☒	☐	ChIP-seq
☒	☐	Flow cytometry
☒	☐	MRI-based neuroimaging

Antibodies

Antibodies used

- Rabbit commercial Peroxidase Anti-Peroxidase (PAP) was purchased from Sigma (Catalog Number P1291; Lot number: 016M4768V; Dilution 1/2000).
- Monoclonal anti-HA High Affinity was purchased from Roche (Catalog Number 11867423001; Clone 3F10; Lot number: 42155800; Dilution 1/40).
- Anti-HA-Peroxidase, High Affinity was purchased from Sigma Aldrich (Catalog number: 12 013 819 001; Clone BMG-3F10; Lot number: 34071100; Dilution 1/250).
- Anti-Dps1p serum was provided by C. Allmang and G. Eriani (IBMC, Strasbourg, France) and used at 1/5000 dilution.
- Anti FLAG-M2 agarose beads were purchased from Sigma (Catalog Number: A2220, Lot number: 060M6081).
- IgG-Sepharose beads were purchased from GE Healthcare (Catalog Number GE17-0969-01; Lot number: 10255966).
- Anti-DDDDK tag (Binds to FLAG® tag sequence) antibody was purchased from Abcam (Catalog number: ab1162; Lot number: GR3225365-2; Dilution 1/1000).
- Anti Histone H3 acetyl Lys56 (H3K56Ac) antibody was purchased from Active Motif (Catalog number: 39281; Lot numbers: 14013003 and 18619005; Dilution 1/50 for IP and 1/1000 for blotting).
- Secondary Antibody: Goat anti-Rabbit IgG (H+L) Cross-Adsorbed Secondary Antibody, HRP was purchased from Invitrogen (Catalog number: A16104; Lot number: 46-183-082415; Dilution 1/20000).

Validation

Information about Anti H5K56Ac antibody:
https://www.activemotif.com/catalog/details/39281/histone-h3-acetyl-lys56-antibody-pab

Information about anti-HA 3F10 antibody:
https://www.sigmaaldrich.com/catalog/product/roche/12158167001?lang=fr&region=FR

Information about PAP: https://www.sigmaaldrich.com/catalog/product/sigma/p1291?lang=fr&region=FR

Blotting with anti-Dps1p led to a single band at the correct apparent size (Fig. S15-4).

Information about Pierce™ Anti-HA Magnetic Beads: https://www.thermofisher.com/order/catalog/product/88836#/88836

Information about Protein G magnetic beads: https://www.thermofisher.com/order/catalog/product/10003D#/10003D

Information about IgG-Sepharose beads: https://www.sigmaaldrich.com/catalog/product/sigma/ge17096901?lang=fr&region=FR

Information about Anti FLAG-M2 agarose beads
https://www.sigmaaldrich.com/catalog/product/sigma/a2220?lang=fr&region=FR
